# Solvent-triggered reconfiguration of optical physical unclonable functions

**DOI:** 10.1038/s41467-026-74463-5

**Published:** 2026-06-20

**Authors:** Ji Hoon Kim, Jihee Kim, Jung Gun Bae, Junsik Choi, Seongsu Cho, Daeyeon Lee, Hyunsik Yoon, Won Bo Lee

**Affiliations:** 1 Department of Chemical and Biological Engineering, Institute of Chemical Processes, Seoul National University, Seoul, Republic of Korea; 2 Department of Chemical and Biomolecular Engineering, Seoul National University of Science and Technology, Seoul, Republic of Korea; 3 Department of Chemical and Biomolecular Engineering, University of Pennsylvania, Philadelphia, PA USA

**Keywords:** Polymers, Mechanical engineering, Polymers

## Abstract

Optical physical unclonable functions provide artificial fingerprints through randomized light–matter interactions, but are limited by static architectures that lack adaptive defense capabilities. Although reconfigurable optical physical unclonable functions based on phase-change materials have been proposed to overcome this constraint, their reliance on light or heat makes them susceptible to unintended environmental activation. Here, we propose a solvent-triggered reconfiguration strategy for optical physical unclonable functions based on polymeric microcube arrays confined within square microwells while retaining translational and rotational degrees of freedom. A volatile solvent induces swelling that establishes wall–cube contact; evaporation-driven detachment drives non-deterministic rearrangement into new spatial configurations, regenerating the optical fingerprint. A machine-learning-based authentication framework provides robust identification of encoded physical configurations. The resulting system exhibits remarkable stability under various environmental and mechanical stresses, while exposure to volatile solvents serves as an effective trigger for reconfiguration, offering a robust pathway to decouple the intrinsic trade-off between environmental stability and reconfigurability.

## Abstract (web summary)

Researchers report a reconfigurable optical anti-counterfeiting tag whose microscopic fingerprint can be rewritten on demand by a droplet of volatile solvent, enabling renewable security without heat, light, or special equipment.

## Introduction

Counterfeiting poses a major threat, extending beyond economic losses to directly endangering lives through tampered medical products^1^. While conventional anti-counterfeiting labels such as QR codes and holograms have been widely adopted, their reliance on deterministic, physically cloneable designs renders them susceptible to forgery^2^. A promising alternative is the physical unclonable function (PUF), which acts as an artificial fingerprint derived from non-deterministic fabrication processes^3^. Among the various types of PUFs^4–9^, optical PUFs (o-PUFs)^10–13^ stand out for their high-dimensional readout, which substantially enhances robustness against forgery compared with single scalar responses such as a voltage or resistance^14^. o-PUFs feature random micro- and nanostructures that interact with light either through resonant photoluminescence^15^ in quantum dots^16,17^, perovskite nanocrystals^17–19^, and other fluorescent taggants^20–22^, or through non-resonant physical optics such as scattering^23^, diffraction^24^, and structural color in disordered photonic media^25,26^. By establishing a set of evaluation metrics for o-PUFs^27^, diverse approaches have been refined through rigorous benchmarking across different o-PUF architectures. Despite these advances, most existing o-PUFs are static architectures and thus remain fundamentally constrained in deploying active defenses after compromise^28,29^. Given that o-PUF tags are physically attached to products, they remain vulnerable to replication attempts. While precise duplication of o-PUFs remains infeasible, advances in nanofabrication have enabled the production of functional clones that pass verification within the tolerance thresholds of field authentication^30^.

This vulnerability has motivated the development of reconfigurable optical PUFs (r-PUFs). Existing r-PUF approaches achieve reconfiguration through responsive media such as liquid crystals, inorganic phase-transition materials, or magneto-optical systems, allowing the encoded spatial configurations to be updated using light^31–34^ heat^29,35–40^, or magnetic fields^9^, as summarized in Table 1. However, reconciling stability with practical reconfigurability remains challenging across these approaches. Light- and heat-based stimuli are inherently coupled to environmental exposure; some liquid crystal-based approaches exhibit low activation thresholds susceptible to unintended reconfiguration^31,39^, while inorganic phase-transition materials typically require harsh conditions incompatible with sensitive goods such as pharmaceuticals^36–38^. Magnetic-based approaches can avoid this environmental coupling but rely on specialized setups for magnetic field generation, constraining field deployability in practical clinical environments^9^.

**Table 1:** Summary of state-of-the-art reconfigurable optical physical unclonable functions

Materials	Encoding capacity	States (per site)	Reconfiguration Stimuli	Stability	Remarks
Smectic LC^35^	2^5700^ [432 × 144 *μm*^*2*^]	8	Thermal (212 °C)	Ambient 7 d; Thermal (100°C 3 min) Humidity (70% RH)	Electrical-optical hybrid
${{Ge}}_{2}{{Sb}}_{2}{{Te}}_{5}$ nanocrystal^36^	2^4147200^ [1 *cm*^*2*^]	2	Thermal (255 °C)	–	–
*VO*_*2*_ nanocrystal^37^	2^655360^	2	Thermal (75 °C)	–	Reversible
Supersaturated sodium acetate solution^38^	2^541^ [28 × 28 μ*m*^2^]	2	Thermal (58 °C)	–	–
PDMS-LC composite^31^	2^1750^ [1 *cm*^2^]	≥10	Optical (60 mW cm^-2^, 470 nm)	–	Reversible
Cholesteric LC^39^	2^184^ [640 × 640 μ*m*^*2*^]	2	Thermal (35 °C)	Thermal (100°C, polymerized)	–
Diamond Microparticles^40^	16^2500^ [100 × 100 μ*m*^*2*^]	16	Thermal (600 °C)	Chemical (1 M NaOH, 1 M HCl, supersaturated NaCl) Thermal (400 °C, 24 h) Light (365 nm & 532 nm, 2 h) Mechanical abrasion	Air oxidation
Graphene-doped LCE^32^	–	2	Optical (30 mW, 980 nm)	Light (<10 mW, 532 nm)	–
Dye-doped PDLC^34^	–	2	Optical (4−25 mW, 450 nm)	–	–
${{Fe}}_{70}{{Ga}}_{30}$ dot array^9^	2^478^ [81 × 60 μ*m*^2^]	3^8^	Magnetic (25 mT)	Thermal (100 °C), UV (385 nm, 40 min)	–
Sintered AuNP array^33^	2^348^ [20 μ*m*^2^]	2	Optical (532 nm)	Thermal (150 °C, 1 h), Chemical (hexane, THF, IPA, water)	–
This work	6^220^ [1.4 *mm*^*2*^];	6	Solvent-triggered (acetone)	Light (15 mW cm^-2^, 365 nm, 24 h) Freezing (-20 °C, 24 h) Thermal (100 °C, 24 h) Humidity (75% RH, 24 h) Mechanical (bending, stretching, peeling, vortexing)	

**Fig. 1:**
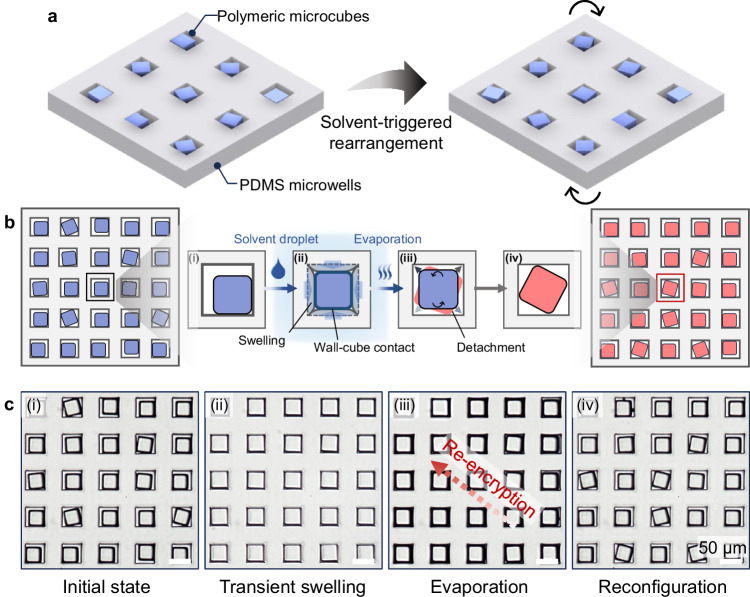
Solvent-triggered reconfiguration of microcubes confined within square microwells. **a** Schematic illustration of microcube arrays under microwell confinement and their solvent-triggered rotational and translational rearrangement. **b** Schematic illustration of the reconfiguration mechanism. Upon exposure to a volatile solvent, polydimethylsiloxane (PDMS) microwell swelling generates compressive stress that drives center-shifting of the microcubes and conformal contact with the lateral walls. As the solvent evaporates, asymmetric detachment from the lateral walls causes the microcube to reconfigure into a new stable orientation. **c** Time-sequenced optical microscopy images showing the reconfiguration process.

In this work, we propose overcoming this trade-off by enabling code reconfiguration with a trace amount of volatile solvent. We design a system in which polymeric microcubes, slightly smaller than the microwell dimensions, are laterally confined within an array of polydimethylsiloxane (PDMS) square microwells (Fig. 1a). This dimensional mismatch introduces free volume, providing the microcubes with translational and rotational degrees of freedom. Upon exposure to a volatile solvent, swelling of both the PDMS microwells and the microcubes reduces the available free volume, leading to wall–cube contact followed by detachment during solvent evaporation (Fig. 1b). This process rearranges the cubes into new configurations within the microwells (Fig. 1c), thereby generating a new optical fingerprint from the same physical tag. This on-demand, solvent-triggered reconfiguration enables a class of r-PUFs that facilitate active defense strategies by operating via a stimulus orthogonal to ambient environmental factors.

## Results

### In situ synthesis of microcubes via oxygen-inhibited polymerization

**Fig. 2:**
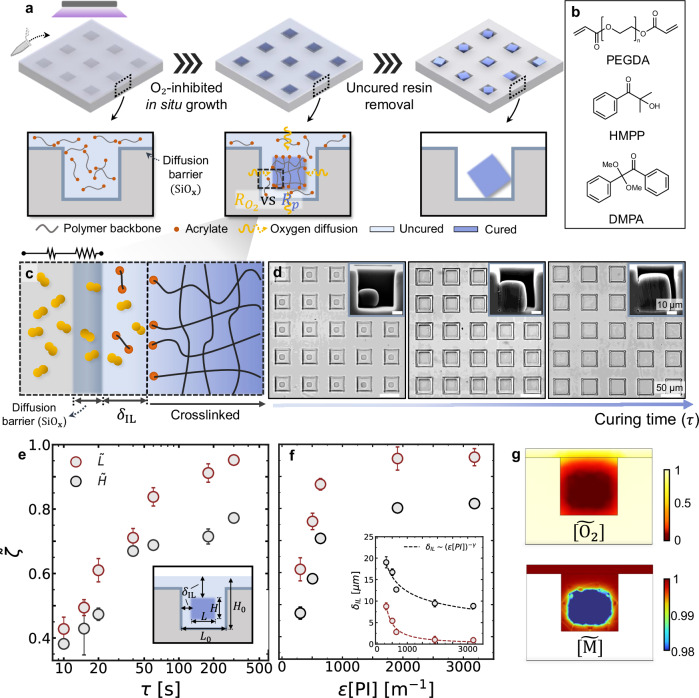
Control of microcube dimensions via oxygen-inhibited in situ polymerization. **a** Schematic illustration of the experimental process, showing the formation of a microcube array through oxygen-inhibited photopolymerization followed by removal of uncured resin through solvent washing. The competition between the oxygen supply rate (${R}_{{O}_{2}}$) and the polymerization rate (${R}_{{{\rm{p}}}}$) governs microcube growth. **b** Precursor materials used in this study: poly(ethylene glycol) diacrylate (PEGDA) and two photoinitiators, 2-hydroxy−2-methylpropiophenone (HMPP) and 2,2-dimethoxy-2-phenylacetophenone (DMPA). **c** Magnified schematic view of the plasma-treated polydimethylsiloxane (PDMS) wall. The silica-like layer formed by plasma treatment acts as a diffusion barrier that modulates radical quenching and determines the thickness of the inhibition layer (${\delta }_{{{\rm{IL}}}}$). **d** Optical microscopy images with corresponding SEM images (inset) showing the volumetric growth of microcubes as a function of curing time τ(10, 40, and 60 s). **e** Normalized microcube dimension $\widetilde{\zeta }$ as a function of τ. **f** Normalized microcube dimension $\widetilde{\zeta }$ as a function of photoinitiation condition $\varepsilon \left[{PI}\right]$. Inset shows $\widetilde{\zeta }$ versus $\varepsilon \left[{PI}\right]$ with power law fits, ${\delta }_{{IL}}$ ~ ${(\epsilon [{PI}])}^{-\gamma }$, where γ is 0.36 for $\widetilde{H}$ and 1.21 for $\widetilde{L}.$ Error bars in (**e**) and (**f**) denote standard deviation; *n* > 20 for $\widetilde{L}$ (optical microscopy) and *n* ≥3 for $\widetilde{H}$ (confocal microscopy). **g** FEA results showing the normalized oxygen concentration $\widetilde{\left[{O}_{2}\right]}$ and normalized monomer concentration $\widetilde{\left[M\right]}$.

A key to enabling the proposed r-PUFs is fabricating a square array of cubic microwells that contain microcubes with controlled geometry. Top-down strategies employing negative pressure or capillarity-assisted loading have previously been used to confine micro-objects in microwells for encoding^41–43^ and single-cell sequencing^44–46^. However, forcing micro-objects into restricted geometries is prone to errors, thereby limiting loading fidelity and increasing fabrication complexity and costs. To overcome this limitation, we design an in situ microcube growth strategy harnessing oxygen-inhibited photopolymerization (Fig. 2a). Poly(ethylene glycol) diacrylate (PEGDA) resin is spin-coated to fill the microwells and form a thin film above them, followed by UV irradiation to initiate photopolymerization. Since oxygen acts as an effective radical quencher^47–49^, gelation proceeds radially from the center of the oxygen-permeable PDMS microwells, while the resin adjacent to the oxygen-rich walls and free surfaces remains uncured due to the presence of oxygen inhibition layers (${\delta }_{{IL}}$). The subsequent removal of these uncured layers results in the formation of microcubes that are smaller than the confining microwell. Due to their translational and rotational freedom, the microcubes can reconfigure within the square microwells upon solvent evaporation. This non-deterministic process forms the fundamental basis of our r-PUF system. We select PEGDA as a representative material owing to its favorable viscosity for uniform coating and compatibility with various photoinitiators (Fig. 2b). We use acetone, a good solvent for PEGDA^50^, to remove the uncured resin, which reduces surface tackiness and enables reliable reconfiguration (discussed in Supplementary Note 1 and Supplementary Fig. 1).

Dimensional control of microcubes within square microwells is achieved by precisely regulating the local oxygen concentration profile and photopolymerization conditions. We introduce a thin silica-like crust layer on the PDMS surface via plasma treatment (Fig. 2c). This layer serves a dual function: it enhances surface wettability to facilitate uniform resin filling and thin-film formation without dewetting (Supplementary Fig. 2) and acts as a diffusion barrier to modulate oxygen diffusion^51^. Consequently, oxygen diffuses freely through the exposed top surface, whereas lateral diffusion is restricted by the composite wall comprising the bulk PDMS and the silica-like barrier. We corroborate this mechanism by observing that prolonged plasma treatment results in minimal lateral ${\delta }_{{{\rm{IL}}}}$, indicating that the resin is fully cured laterally within the well and thereby exhibits growth exclusively along the z-direction under otherwise identical conditions (Supplementary Fig. 2).

To quantify the cured dimensions, we define normalized lateral and vertical cube dimensions as $\widetilde{L}=L/{L}_{0}$ and $\widetilde{H}=H/{H}_{0}$, respectively, where ${L}_{0}$ is the square microwell width and ${H}_{0}$ is the sum of the microwell depth and liquid film thickness formed above the microwells. Microcubes exhibit sustained volumetric growth with increasing curing time (τ), accompanied by a simultaneous reduction in ${\delta }_{{{\rm{IL}}}}$, even as the growth rate diminishes (Fig. 2d–e and Supplementary Fig. 3). This behavior contrasts with that reported in previous studies where growth stagnates^47^ (resembling a knee-shaped curve) or ${\delta }_{{{\rm{IL}}}}$ remains constant after the gelation induction time^48^. This sustained growth is attributed to the restricted oxygen diffusion imposed by the silica-like barrier, which causes continuous oxygen depletion within the microwell.

We further investigate the influence of photoinitiation conditions on microcube growth. This dependency can be rationalized within a well-established theoretical framework based on the Damköhler number^47^, defined as

1 $${{\rm{Da}}}=\frac{\phi \epsilon [{PI}]{{I}_{0}\zeta }_{0}^{2}}{{D}_{{O}_{2},{{\rm{eff}}}}[{O}_{2,{{\rm{eqb}}}}]}$$

where ${\zeta }_{0}$ is the characteristic length scale of the system, ϕ, ϵ, and [PI] denote the quantum yield, molar extinction coefficient, and concentration of the photoinitiator, respectively, ${I}_{0}$ is the incident light intensity, ${D}_{{{{\rm{O}}}}_{2},{{\rm{eff}}}}$ is the effective diffusion coefficient of oxygen within the microwell, and $[{O}_{2,{{\rm{eqb}}}}]$ is the equilibrium oxygen concentration. Well-known scaling laws predict ${\delta }_{{{\rm{IL}}}}\sim {{{\rm{Da}}}}^{-\gamma }$^47,52,53^, which simplifies to ${\delta }_{{{\rm{IL}}}}$ ~ ${(\epsilon [{PI}])}^{-\gamma }$ assuming other factors remain constant. The exponent γ is typically approximated as 0.5 for oxygen-inhibited photopolymerization, in which oxygen is supplied to the resin interface from bare PDMS^47,52^. The best-fit exponent for our system is γ=0.36 for $\widetilde{H}$ and 1.21 for $\widetilde{L}$ (Fig. 2f and Supplementary Fig. 4); the lower exponent for $\widetilde{H}$ reflects the direct, continuous oxygen flux from the ambient free surface, providing an unhindered oxygen supply. In contrast, lateral growth is strictly regulated by the sidewall diffusion, resulting in a significantly sharper power-law dependence. Consistent with this diffusion-limited behavior, $\widetilde{L}$ consistently exceeds $\widetilde{H}$ across all conditions, as shown in Fig. 2e–f.

Finite element analysis (FEA) further supports this interpretation. Building on the previously developed model^47^, we incorporate a diffusion barrier to simulate a silica-like layer along the lateral and bottom surfaces of the microwell. We compute the normalized oxygen and monomer concentrations within the microwell, defined as $\widetilde{\left[{O}_{2}\right]}=[{O}_{2}]/{[{O}_{2}]}_{{{\rm{eq}}}}$ and $\widetilde{\left[M\right]}=[M]/{[M]}_{0}$, respectively, where $[M]_{0}$ is the initial monomer concentration. This barrier induces a pronounced reduction in oxygen transport, resulting in preferential oxygen depletion and monomer consumption, initiating gelation from the center of the well (Fig. 2g). Based on the reported gelation threshold of PEGDA (1–2% double-bond conversion)^54^, we set the gelation threshold at $\widetilde{\left[M\right]}=0.985$. The resulting simulated gelation behavior reproduces microcube growth as a function of τ and the trend of $\widetilde{L}$ exceeding $\widetilde{H}$, consistent with experimental observations (see Supplementary Note 2 and Supplementary Fig. 5 for details).

### Mechanism and optimization of solvent-triggered reconfiguration

**Fig. 3:**
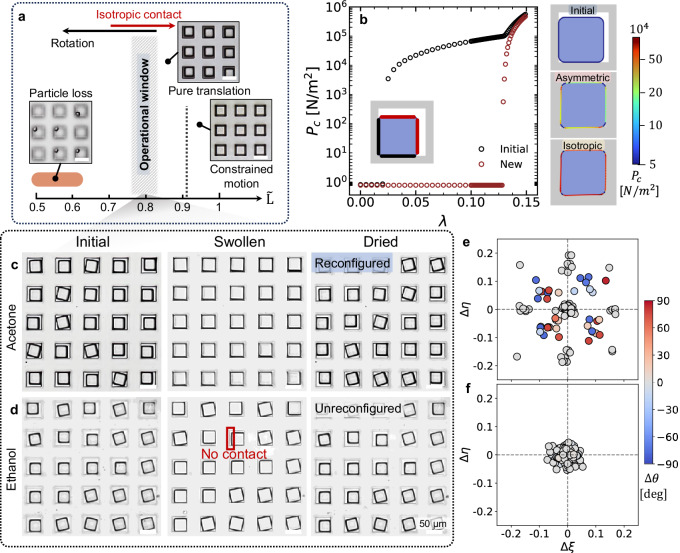
Mechanism of the solvent-triggered reconfiguration process. **a** Conceptual schematic illustrating microcube motion regimes as a function of the normalized lateral dimension, $\widetilde{L}$. **b** FEA results showing contact pressure (${P}_{c}$) as a function of linear expansion ratio (λ); sufficient swelling induces isotropic wall–cube contact by involving both initial and newly contacted lateral walls. **c**, **d** Optical microscopy images showing microcube configurations in the initial, swollen, and dried states following acetone (**c**) and ethanol (**d**) exposure. Acetone induces isotropic wall contact and subsequent reconfiguration upon evaporation, whereas ethanol results in insufficient contact and microcube immobilization. **e**, **f** Distribution plots quantifying changes in translation ($\varDelta \xi,\varDelta \eta$) and rotation ($\varDelta \theta$) after acetone (**e**) and ethanol (**f**) treatment.

The dynamics of microcubes upon exposure to a volatile solvent are governed by the interplay between lateral spatial confinement, quantified by $\widetilde{L}$, and the swelling of the PDMS microwells and PEGDA microcubes driven by solvent affinity. An optimal reconfiguration window exists in which swelling ensures isotropic wall–cube contact while allowing rotation (Fig. 3a). Microcubes with $\widetilde{L}$ < 0.6 are prone to washout (>50% loss) during washing. In contrast, those with $\widetilde{L}$ > 0.9 remain immobilized or do not undergo rotation due to the lack of free volume (Supplementary Fig. 6). Assuming a simplified 2D model of rigid squares with sharp corners within rigid square microwells, the geometric limit for in-plane rotation is $\widetilde{L}$ < 1/$\sqrt{2}$ (≈ 0.71); however, we observe rotational reconfiguration even at $\widetilde{L}\approx 0.82$. We attribute this discrepancy to the rounded corners of the microcubes and the elastic deformation of the softer PDMS microwells upon contact with the higher-modulus PEGDA microcubes^49^.

2D FEA contact simulations demonstrate that sufficient swelling induces isotropic wall–cube contact (Fig. 3b). When the cube is initially positioned at a corner with two sides contacting adjacent walls, inward bulging of the walls drives the cube toward the center as the linear expansion ratio (λ) of PDMS increases. Beyond a critical linear expansion ratio (${\lambda }_{{crit}}$), the available free volume diminishes, and the cube comes into contact with the remaining two sidewalls. Consequently, the contact pressure $({P}_{c})$, defined as the normal contact stress at the interface, becomes comparable across all lateral walls (see Supplementary Fig. 7 and Methods for details).

Guided by these predictions, we compare reconfiguration dynamics under two solvents that induce markedly different swelling responses in the PDMS microwells and PEGDA microcubes. Under acetone exposure, which induces substantially greater swelling than ethanol (Supplementary Fig. 8), the microcube shifts toward the center and contacts the lateral walls in the swollen state (Fig. 3c). Detachment from the bottom wall occurs rapidly during this process (Supplementary Fig. 9 and Supplementary Movie 1). As evaporation proceeds, the contacts detach sequentially; this asymmetric detachment generates unbalanced forces and torque that drive the microcube to translate or rotate into a new state, resulting in non-deterministic reconfiguration (Supplementary Movie 2). In contrast, ethanol results in limited swelling that fails to establish wall–cube contact, leaving the microcubes pinned in their original positions (Fig. 3d).

To quantify reconfiguration, we define the relative translational displacements $\varDelta \xi$ and $\varDelta \eta$ as the normalized in-plane shifts of the microcube center along two orthogonal axes, and the rotational change $\varDelta \theta$ as the in-plane rotation relative to the original state. The resulting distribution plots confirm that acetone induces substantial reconfiguration in both translation and rotations (Fig. 3e and Supplementary Movie 3). Conversely, ethanol results in negligible reconfiguration, leaving the microcubes immobilized (Fig. 3f and Supplementary Movie 4).

### Machine-learning-assisted authentication and performance evaluation of physical unclonable functions

**Fig. 4:**
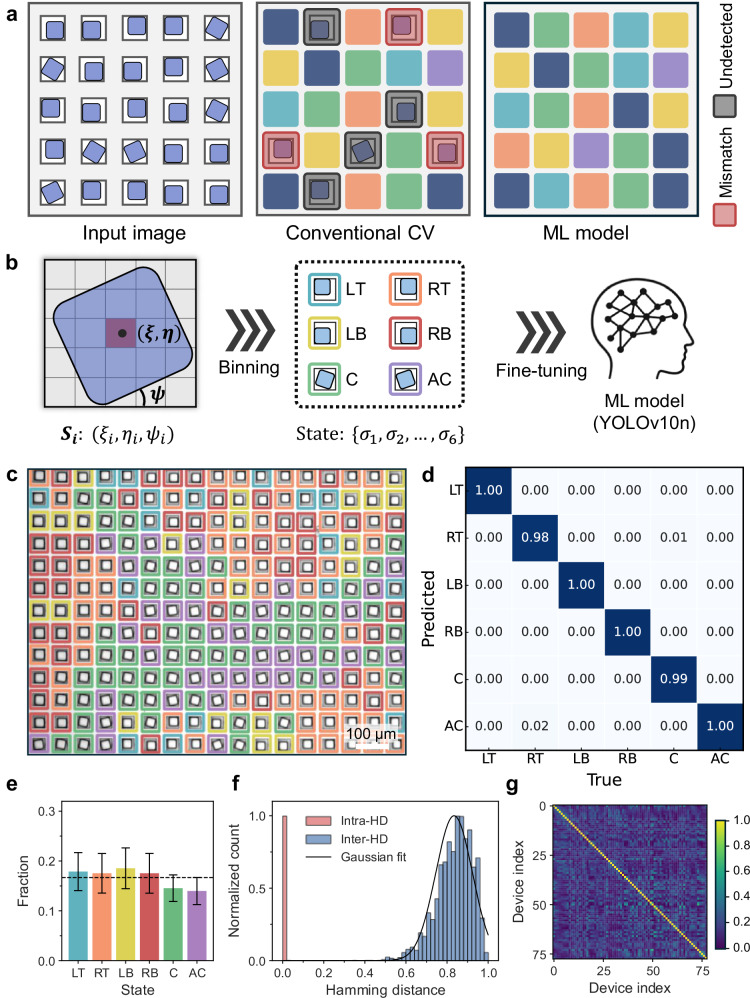
Machine-learning-assisted authentication and performance evaluation of physical unclonable functions. **a** Comparative schematics of detection performance between conventional computer vision (CV)-based patch identification and the fine-tuned machine learning (ML) model. **b** Discretization of the continuous microcube configuration space into six distinct states for fine-tuning the YOLOv10n object detection model. **c** Representative experimental PUF image showing classification of 221 elements (13 × 17 array) into the six defined states. **d** Confusion matrix demonstrating classification accuracies exceeding 98% across all states. **e** State probability distribution. Error bars denote 95% confidence intervals. **f** Distributions of Intra-Hamming distance (Intra-HD) and Inter-Hamming distance (Inter-HD) of the PUF responses. **g** Cross-correlation map of PUF responses.

Conventional image processing for PUF pattern identification suffers from inevitable mismatches and undetected regions, limiting its practicality for reliable authentication. To overcome these limitations, we implement a machine-learning(ML)-based authentication framework that significantly enhances both the robustness and accuracy of PUF code extraction (Fig. 4a). While the microcube configuration space is defined as continuous ${S}_{i}({\xi }_{i},{\eta }_{i},{\psi }_{i})$ (where $0 < {\xi }_{i},{\eta }_{i} < 1$ and $0^\circ < {\psi }_{i} < 90^\circ$), we discretize these configurations into six states: four corner-contact states, LT, RT, LB, and RB, and two rotated states, C and AC, corresponding to clockwise and anti-clockwise rotations, respectively. Based on this classification, we fine-tune an object detection model (YOLOv10n) (Fig. 4b) using a combinatorial data generation strategy. In this process, we exclusively crop individual cube-well pair images that are successfully identified by conventional computer vision algorithms and randomly recombine them into synthetic grid arrays to form a robust training dataset (see Supplementary Fig. 10 for details). By excluding the inevitable mismatches and undetected regions inherent in traditional methods, this approach creates error-free ground-truth data and enables up to O(N!) unique permutations from a single source image, offering a distinct advantage over prior studies that require extensive raw image acquisition^55,56^. Using this approach, 221 elements are accurately detected and classified into the six distinct states (Fig. 4c). The confusion matrix (Fig. 4d) demonstrates classification accuracies exceeding 98% across all states, with the fine-tuned model achieving a mean Average Precision (mAP) of 99%, confirming reliable localization and classification (see Supplementary Fig. 11 for extended metrics).

Based on the ML-based recognition results, we evaluate the PUF performance metrics. To assess randomness, we quantify the state uniformity of each state from 80 different PUF codes. Although corner states appear slightly more frequent than rotated states, the 95% confidence intervals for all six states include the ideal probability of 1/6 ( ≈ 0.1667), indicating comparable state probabilities (Fig. 4e). We further quantify the state-distribution uniformity using Shannon entropy. The calculated entropy is 2.577 bits, close to the ideal value of log_2_6 ( ≈ 2.585 bits), corresponding to an effective encoding capacity of ${6}^{220}$ within a compact footprint of approximately 1.4 mm^2^, ensuring sufficient density for secure authentication (see “Methods”). To assess reliability, we quantify the similarity between repeated readouts of a single PUF code using the Intra-Hamming distance (Intra-HD), defined as the fraction of mismatched elements between two PUF codes (see “Methods”). The Intra-HD, calculated from 50 repeated responses of a single PUF code, yields a low mean of 0.0036 ± 0.0049, indicating high response stability and reliability (Fig. 4f). To evaluate uniqueness, we analyze the Inter-Hamming distance (Inter-HD) across 80 different PUF codes. The generalized Inter-HD is 0.8341 ± 0.0892, which approximates the theoretical value of 5/6( ≈ 0.8333) for a 6-ary system (Fig. 4f). We note that Inter-HD quantifies uniqueness across different PUF tags; code variation upon repeated reconfiguration is discussed in the following section. Furthermore, the pairwise correlation matrix of the encoded 221-element PUF responses exhibits a prominent diagonal, indicating that high correlations occur only for identical patterns (Fig. 4g).

### Proof-of-concept application and stability testing

**Fig. 5:**
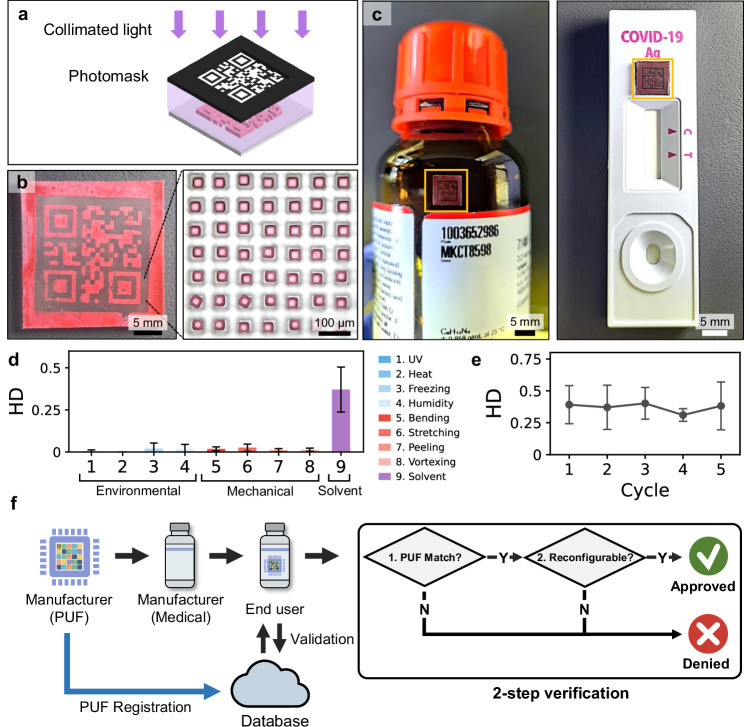
Proof-of-concept for practical application: Hybrid QR–PUF tags, stability testing, and two-step verification protocol. **a** Schematic of hybrid tag fabrication via mask-assisted lithography. **b** Photograph and magnified optical microscopy image of the fabricated quick response–physical unclonable function (QR–PUF) hybrid tag. **c** Demonstration of compatibility through attachment of the hybrid tags onto reagent bottles and diagnostic kits. **d** Hamming distance (HD) values confirming structural stability under environmental stressors (1–4) and mechanical perturbations (5–8), contrasted with pronounced reconfiguration after solvent exposure (9). Bars and error bars represent the mean and standard deviation, respectively; *n* = 3 independent measurements for the stability tests (1–8) and *n* = 25 (5 samples × 5 cycles) for solvent triggering (9). **e** Cycle-to-cycle HD over 5 consecutive solvent-triggered reconfiguration cycles. Error bars denote standard deviation (*n* = 5 samples per cycle). **f** Schematic of the proposed two-step verification protocol. The database first verifies whether the scanned tag code matches a registered code, and then the end user applies a solvent droplet to test reconfigurability. Only authentic tags undergo successful reconfiguration, while counterfeit replicas fail to reconfigure and are denied.

While PUFs offer superior anti-counterfeiting performance, their lack of direct visual readability can limit field applicability. To address this potential issue, we fabricate a QR-PUF hybrid tag using mask-assisted lithography (Fig. 5a). By incorporating a visualization dye, the tag features a QR code as a scannable visual identifier that links to the PUF verification system, while the embedded microscopic PUF provides high-security authentication. (Fig. 5b). Notably, the QR code retains its readability even after solvent-triggered reconfiguration, confirming its independence from the PUF microstates (Supplementary Fig. 12). We evaluate the compatibility of these hybrid tags in real-world settings by attaching them to reagent bottles and diagnostic kits; the tags exhibit stable adhesion across various materials and curved surfaces (Fig. 5c). Furthermore, the tags demonstrate robust mechanical integrity, with encoded patterns remaining intact after repeated tape pull tests (100 cycles), cyclic bending (radius: 0.4 mm, 100 cycles), and cyclic stretching (20% strain, 50 cycles), and vortex-induced vibration (~2000 rpm, 30 min) (Supplementary Fig. 13); detachment occurs only under bath sonication in aqueous media, consistent with prior reports on microparticle harvesting from PDMS molds via ultrasonic agitation^57^ (Supplementary Fig. 14).

Distinct from conventional o-PUFs that often rely on labile photoactive materials, our system employs a crosslinked polymer matrix free from such additives, ensuring exceptional stability against various environmental stressors. To validate this, we subject the PUFs to a suite of rigorous stress tests: UV irradiation (365 nm, 15 mW/cm² for 24 h), high-temperature exposure (100 °C for 24 h), freezing (−20 °C for 24 h), and high humidity (75% RH for 24 h). The encoded patterns remain nearly unchanged under these harsh conditions, as confirmed by our fine-tuned detection model (Fig. 5d and Supplementary Fig. 15). Quantitative analysis based on the Hamming distance (HD) further confirms that the encoded patterns remain nearly unchanged under both environmental stressors and mechanical perturbations, with the mean HD for each stress condition remaining below 0.026, whereas acetone exposure induces pronounced reconfiguration of the encoded pattern (Fig. 5d). To evaluate the repeatability and cycle-to-cycle variability of this process, we perform 5 consecutive solvent-triggered reconfiguration cycles on independent samples. The HD between consecutive cycles remains approximately 0.37, with no decreasing trend, confirming persistent cycle-to-cycle variation (Fig. 5e, and Supplementary Fig. 16, and Supplementary Movie 5). To further assess statistical dependence between consecutive reconfiguration cycles, we also calculate normalized mutual information (MI), which quantifies the dependence between site-state transitions before and after each cycle. The average MI is ~0.33, where 0 denotes statistical independence and 1 denotes complete dependence (Supplementary Fig. 17). The cycle-to-cycle HD is lower than the ideal value of 5/6 for a fully random 6-ary system, and the measured MI indicates residual statistical dependence between consecutive responses. Nevertheless, the residual configurational space is estimated to be ~${2}^{381}$, which exceeds the 256-bit security-strength level commonly referenced by the National Institute of Standards and Technology (NIST)^58^, supporting practical resistance against exhaustive prediction-based attacks (see “Methods” for details).

## Discussion

Overall, this work establishes a solvent-driven r-PUF architecture to effectively decouple reconfigurability from environmental stability. We construct an array of microcubes confined within microwells through controlled oxygen-inhibited polymerization, thereby enabling precise dimensional control that directly governs the degrees of freedom of the microcubes. We experimentally and computationally elucidate the mechanism underlying solvent-triggered reconfiguration dynamics; after isotropic wall–cube contact via transient swelling, the sequential detachment of the lateral walls during evaporation breaks the contact symmetry, causing the microcube to drift or rotate toward a non-deterministic position. Integrated with an ML-based authentication framework, our system achieves high classification accuracy (~99%) and demonstrates practical utility in anti-counterfeiting applications, exhibiting robust stability under mechanical, thermal, optical, and humidity stresses.

In contrast to conventional systems that rely on external energy inputs such as light, heat, or magnetic fields and require dedicated instrumentation for reconfiguration at the point of use, the solvent-triggered approach is orthogonal to ambient environmental factors and requires only a droplet of volatile solvent, which can be supplied as a compact verification kit accompanying the product. This makes it readily implementable in pharmaceutical and medical supply chains, preventing unintended code changes during transport while enabling on-demand reconfiguration accessible to end users without specialized equipment.

Based on these capabilities, we propose a two-step verification protocol that enables active post-compromise defense (Fig. 5f). Once the PUF tags are manufactured, their initial codes are registered in an authentication database, and the tags are subsequently attached to medical products. When an authorized end user (e.g., a physician) receives a tagged product, authentication is performed before use. First, the end user scans the tag, and the database verifies whether a matching code exists; a tag with an unregistered code is immediately rejected. To verify that the tag is not a clone replicating a registered code, the end user then applies a solvent droplet to test its reconfigurability. Successful reconfiguration occurs only for an authentic tag based on our r-PUF architecture, and the product is approved for use. In contrast, a counterfeit tag fabricated as a monolithic replica fails to undergo reconfiguration and is denied. Even non-monolithic replication would require reproducing the geometry and placement of individual microcubes, as well as the spatial crosslink-density gradients and cube-well interfacial properties. These features critically govern reconfiguration dynamics, yet are not readily accessible via conventional bulk and surface characterization.

We envision this strategy as a generalizable anti-counterfeiting platform. Although PEGDA serves as a model material, we demonstrate applicability to other acrylate-based resins (Supplementary Fig. 18). Future studies could explore geometric design strategies, such as high-density grid arrays with reduced inter-particle spacing that undergo collective buckling, enabling reconfiguration with milder solvents^59,60^. By elucidating the fundamental mechanism of solvent-triggered reconfiguration, this work establishes a solid foundation for field-deployable r-PUFs for high-security authentication.

## Methods

### Materials

SU-8 3050 photoresist was purchased from Kayaku (Microchem). Polydimethylsiloxane (PDMS, Sylgard 184) was purchased from Dow Corning. Poly(ethylene glycol) diacrylate (PEGDA, average molecular weight = 700 g ${{{\rm{mol}}}}^{-1}$) and photoinitiators, 2-hydroxy-2-methylpropiophenone (HMPP) and 2,2-dimethoxy-2-phenylacetophenone (DMPA) were purchased from Sigma-Aldrich. Polyurethane acrylate (PUA)-based resin (MINS-301) was obtained from MCNet Co., Ltd. Perfluoropolyether (PFPE)-urethane dimethacrylate (Fluorolink MD 700) was purchased from Solvay. Norland Optical Adhesive 74 (NOA 74) was purchased from Norland Products. All chemicals were used as received without further purification, unless otherwise indicated.

### In situ formation of microcube arrays for physical unclonable functions

A master mold was fabricated on a silicon wafer using SU-8 3050 photoresist via standard photolithography following the manufacturer’s guidelines. After post-baking at 150 °C for 1 h, an elastomeric negative mold was created by casting polydimethylsiloxane (PDMS) prepolymer mixed with a curing agent (10:1 w/w) over the master mold, followed by thermal curing at 65 °C for 4 h. Poly(ethylene glycol) diacrylate (PEGDA) prepolymers were prepared by mixing with two types of photoinitiators, 2-hydroxy-2-methylpropiophenone (HMPP) or 2,2-dimethoxy-2-phenylacetophenone (DMPA), at concentrations of 1, 3, and 5 wt%. The solution was deposited onto the PDMS molds after oxygen plasma treatment (CUTE, Femtoscience, 100 W, 50 sccm) for 10 s and covered with a polyethylene terephthalate (PET) film. The mold was placed in a vacuum chamber (~10⁻^2^ torr) for 2 min to remove bubbles and fill the microwells with the resin. For the unidirectional growth in Supplementary Fig. 2, the plasma treatment time was increased to 60 s, with all other conditions kept identical. Subsequently, the PET film (thickness: 50 µm) was removed, and an additional 200 µL of PEGDA resin solution was applied, followed by spin-coating at 2000 rpm for 30 s (acceleration: 10 s). The mold coated with a thin resin film was immediately exposed to ultraviolet (UV) irradiation for 300 s (using a Fusion Cure 360, MINUTA Tech, 10 mW/cm², ultraviolet A (UVA) range). After curing, the sample was gently washed with acetone to remove uncured resin, resulting in a randomized microcube array confined within the PDMS microwells.

The other acrylate-based resins (polyurethane acrylate (PUA) and Norland Optical Adhesive 74 (NOA 74)) were used as received, without adding photoinitiator. The PDMS molds were plasma-treated for 20 s, followed by spin-coating at 2000 rpm and UV curing for 40 s under the same conditions described above. PEGDA microcubes ($\widetilde{L}\approx 0.8$) for reconfiguration experiments were also prepared using an alternative experimental setup. In this configuration, oxygen plasma treatment was performed using a plasma cleaner (PDC-32G, Harrick Plasma) for 8 s at high radio frequency (RF) intensity. Photopolymerization was induced using a spot UV curing system (OmniCure Series 1500, Excelitas Technologies) for 5 s at a relative intensity of 25.

### Solvent-triggered reconfiguration of microcube arrays

For reconfiguration experiments, samples were placed on the stage of an optical microscope, and 10 μL of acetone or ethanol was deposited using a micropipette while the structural response was observed in real time. For repeated cycles, the same volume of solvent was reapplied after solvent evaporation, or reconfiguration was confirmed.

### Swelling ratio measurement

The transition from rapid swelling to saturation can be described by a characteristic time scale, *t*_*c*_~*L*^*2*^/D₀^61^. For macroscopic samples, slow swelling kinetics limit accurate measurements; therefore, micropillar patterns (the inverse of the microwell geometry) composed of polydimethylsiloxane (PDMS) and poly(ethylene glycol) diacrylate (PEGDA) were prepared via a consecutive soft lithography process. For L~45 μm, ${t}_{c}$ is on the order of seconds. Pillar diameters were measured prior to and 30 s after solvent droplet deposition, and the swelling ratio was determined from the normalized diameter change (*l/l₀*).

### Environmental stability tests

We assessed the robustness of the physical unclonable functions (PUFs) under four categorized stress conditions: (i) Thermal: 100 °C for 24 h; (ii) Optical: ultraviolet (UV) irradiation (365 nm, 15 mW/cm²) for 24 h; (iii) Humidity: 75% relative humidity (RH) for 24 h; (iv) Freezing: -20 °C for 24 h.

### Mechanical stability tests

We evaluated the mechanical robustness of the physical unclonable function (PUF) tags under five stress conditions: (i) Peel: repeated adhesion and removal using 3 M tape (100 cycles); (ii) Bending: cyclic bending based on American Society for Testing and Materials (ASTM) D522 (radius: 0.4 mm, 100 cycles); (iii) Stretching: cyclic tensile deformation (20% strain, 50 cycles); (iv) Vortex: vortex mixing (~2000 rpm, 30 min). (v) Sonication: bath sonication in aqueous media (50–60 kHz, 30 min). After each test, the encoded patterns were imaged and analyzed using the fine-tuned detection model to assess pattern integrity.

### Fabrication of quick response-physical unclonable function hybrid tag

To ensure compatibility with a diagnostic kit or a reagent bottle, a polydimethylsiloxane (PDMS) microwell sample with a total thickness of 500 µm was prepared. A resin was formulated using poly(ethylene glycol) diacrylate (PEGDA) mixed with 3 wt% 2-hydroxy-2-methylpropiophenone (HMPP) as a photoinitiator; Nile red was added for visualization. The solution was introduced into the microwells following the same procedure described for the microcube array fabrication, which included oxygen plasma treatment, vacuum infiltration, and spin coating. Subsequently, a film mask with an inverted quick response (QR) code pattern (8 × 8 mm² and 20 × 20 mm²) was placed in soft contact with the sample. The assembly was exposed to collimated ultraviolet (UV) light for 70 s using a mask aligner (MDA400M, MIDAS System). Finally, the sample was washed with acetone to remove uncured resin, yielding the quick response–physical unclonable function (QR–PUF) hybrid tag. The diagnostic kits were purchased from SD Biosensor (Korea).

### Statistical evaluation of physical unclonable function performance 1)

Hamming Distance: The uniqueness of the physical unclonable functions (PUFs) was quantified using the generalized Inter-Hamming distance (Inter-HD), defined as

2 $${{\rm{Inter}}}-{{\rm{HD}}}=\frac{2}{N(N-1)}{\sum }_{i=1}^{N-1}{\sum }_{j=i+1}^{N}\frac{{HD}\left({R}_{i},{R}_{j}\right)}{n}$$

where ${R}_{i}$ and ${R}_{j}$ are the n-element keys of the i-th and j-th PUFs (n=221), and N is the total number of PUF images (N=80). For a q-ary system with equiprobable states, the probability that two independently generated sites are in different states is (q−1)/q. Since the fractional Hamming distance is the fraction of differing sites, the expected Inter-HD is (q−1)/q, yielding a theoretical value of 5/6≈0.833 for q=6.

The reliability of the PUFs was evaluated using the generalized Intra-Hamming distance (Intra-HD), which measures the similarity between repeated responses obtained from the same PUF device. The Intra-HD is defined as

3 $${{\rm{Intra}}}-{{\rm{HD}}}=\frac{1}{M}{\sum }_{i=1}^{M}\frac{{HD}\left({R}_{0},{R}_{i}\right)}{n}$$

where ${R}_{0}$ is the reference n-element key of a given PUF (n=221), and ${R}_{i}$ is the key obtained from the same PUF during the i-th measurement among a total of M=50 repeated measurements. All responses are compared with the reference key ${R}_{0}$.

2) Effective encoding capacity: To quantify the deviation from ideal encoding capacity, we computed the multistate Shannon entropy per site^62^: $H=-{\sum }_{s=1}^{q}{p}_{s}{\log }_{2}{p}_{s}$, where ${p}_{s}$ is the probability of observing the state s across the dataset and q=6. The ideal entropy for equiprobable states is ${H}_{\max }={\log }_{2}6=2.585$ bits. The total configurational entropy is ${H}_{{\mbox{total}}}=n\cdot H$, where n=221 is the number of sites. The effective number of independent configurational states is then defined as ${{{\rm{N}}}}_{{\mbox{eff}}}={{{\rm{H}}}}_{{\mbox{total}}}/{\log }_{2}6$ and the effective encoding capacity as ${C}_{{\mbox{eff}}}={2}^{{H}_{{\mbox{total}}}}$. Based on 80 PUF codes, we obtained H=2.577 bits and ${N}_{{\mbox{eff}}}=220$, yielding ${C}_{{\mbox{eff}}}={6}^{220}$.

3) Cycle-to-cycle independence: To evaluate statistical dependence between consecutive cycle outputs, we computed the normalized mutual information (MI) from the 6 × 6 transition matrix of site states between two successive cycles:

4 $${MI}=\frac{1}{{\log }_{2}6}{\sum }_{i=1}^{6}{\sum }_{j=1}^{6}p\left(i,j\right){\log }_{2}\left(\frac{p\left(i,j\right)}{{p}_{t}(i){p}_{t+1}(j)}\right)$$

where p(i,j) is the joint probability that a site is assigned to the state i at cycle t and state j at cycle t+1, and ${p}_{t}(i)$ and ${p}_{t+1}(j)$ are the corresponding marginal probabilities. For each cycle pair, p(i,j) is obtained by counting the state transitions across 221 sites and normalizing by 221. By this definition, MI = 0 indicates statistical independence, whereas MI = 1 indicates complete dependence. The measured MI values range from ~0.30 to 0.37 across 5 cycles, with an average value of ~0.33. Using the measured state-distribution entropy of 2.577 bits per site, the residual conditional uncertainty is estimated as $2.577-0.33{\times \log }_{2}6\approx 1.72$ bits per site. Across 221 sites, this gives a total residual uncertainty of $221\times 1.72\approx 381$ bits, corresponding to a residual configurational space of ~${2}^{381}$, exceeding the 256-bit security-strength level commonly referenced in National Institute of Standards and Technology (NIST) cryptographic key-generation guidance^58^.

### Characterization

Optical microscopy (OM; Olympus BX41M) was used to acquire OM images and observe the reconfiguration process (Supplementary Movie 1 and 2). High-speed imaging of bottom-wall adhesion release was performed using a high-speed camera (Fastcam Mini AX200, Photron) attached to an optical microscope (ECLIPSE TE200, Nikon). Scanning electron microscopy (SEM; EM-30, COXEM, Republic of Korea) was conducted at an acceleration voltage of 10 kV to obtain SEM images. Confocal images were acquired using a three-dimensional (3D) surface confocal laser scanning microscope (LSM 800 MAT, Carl Zeiss) with a magnification of ×100.

### Data pre-processing and synthetic dataset generation

All image processing pipelines were implemented in Python using OpenCV and scikit-learn. 1) Patch Identification: Optical micrographs of 13 × 17 unit-cell arrays were segmented using adaptive thresholding, and individual unit cells were extracted by contour analysis. Detected objects were classified into microwells and microcubes using K-means clustering. Cube orientation was quantified using principal component analysis (PCA). Only valid well–cube pairs were retained for subsequent annotation. 2) Binning and Augmentation: The cropped patches were sorted into six classes based on alignment and orientation: left-top (LT), right-top (RT), left-bottom (LB), right-bottom (RB), clockwise (C), and anti-clockwise (AC), with accuracy verified by human supervision. Augmentation techniques including Gaussian blur, noise addition, and contrast adjustment were applied to balance the class distribution, resulting in 1843 patches per class. 3) Reconstruction: The augmented patches were utilized to generate synthetic training data. Patches were randomly arranged into 3 × 3, 4 × 4, and 5 × 5 grid arrays with auto-generated annotations. This process yielded a total of 22,000 labeled images (20,000 for training and 2000 for validation).

### Object detection

The YOLOv10n object detector, initialized with pre-trained weights from YOLO11n, was fine-tuned using the PyTorch framework in a Google Colab Pro environment equipped with a single NVIDIA A100 graphics processing unit (GPU). The training protocol used the stochastic gradient descent (SGD) optimizer with a batch size of 64 and an input resolution of 1280 × 1280 pixels for up to 300 epochs, with early stopping patience set to 40 epochs. Mosaic augmentation was retained to increase effective sample diversity. The weights yielding the highest validation performance were selected for downstream analysis.

### Validation and post-processing

For validation-set inference and confusion-matrix analysis, predictions were generated at an input resolution of 1280 pixels using a confidence threshold of 0.25. Predicted boxes were matched to ground-truth boxes using an intersection over union (IoU) threshold of 0.5. The resulting 6 × 6 confusion matrix excluded background false-positive and false-negative entries.

### Finite-element simulation of swelling-induced contact

Finite element analysis (FEA) was performed using the Solid Mechanics interface of the Structural Mechanics module in COMSOL Multiphysics (v. 6.0). The polydimethylsiloxane (PDMS) microwell was modeled as a hyperelastic material using a Neo-Hookean constitutive model, while the poly(ethylene glycol) diacrylate (PEGDA) microcube was treated as a linear elastic material. For simplicity, the PEGDA cube geometry was prescribed to represent the swollen state, with a lateral dimension of 36 µm, a fillet radius of 3 µm, and positioned inside a 45 µm square microwell. Swelling-induced deformation of PDMS was implemented by imposing a linear expansion ratio in the range of λ=0−0.15. The strain energy density of PDMS was decomposed into shear (distortional) energy and volumetric energy, expressed as $W=\frac{1}{2}{\mu }_{L}({I}_{1}-3)+\frac{1}{2}{\lambda }_{L}{\left[{\mathrm{ln}}({J}_{{el}})\right]}^{2},$ where ${\mu }_{L}$ and ${\lambda }_{L}$ are the Lamé parameters, ${I}_{1}$ is the first invariant of the deviatoric deformation tensor, and ${J}_{{el}}$ is the elastic volume ratio. Geometric nonlinearity was included to accurately capture large deformations and the evolution of contact during swelling. Contact between the PEGDA microcube and the PDMS walls was modeled using the penalty method, which applies a restoring force upon surface interpenetration. Adhesion was incorporated using an activation criterion with a characteristic length scale of $1\times {10}^{-8}\,{{\rm{m}}}$. Frictional interactions were described by a Coulomb friction model with a friction coefficient of 0.01, chosen to approximate the transient, solvent-lubricated polymer contact conditions during acetone-induced swelling^63^. Material properties for PDMS were taken from the COMSOL material library, while those for PEGDA were obtained from literature sources^64^.

## Supplementary information

Supplementary Information

Description Of Additional Supplementary File

Supplementary Movie 1

Supplementary Movie 2

Supplementary Movie 3

Supplementary Movie 4

Supplementary Movie 5

Transparent Peer Review file

## Source data

Source data

## Data Availability

The data supporting the findings of this study are provided in the paper, Supplementary Information, and Source Data file. Source data are provided with this paper.
