# Supplementary material for: Solvent-triggered reconfiguration of optical physical unclonable functions
Source: Nat Commun. 2026 Jun 20;17:7826. doi: 10.1038/s41467-026-74463-5 (PMC13438844; doi:10.1038/s41467-026-74463-5)
Supplement: Supplementary file 1 [file 41467_2026_74463_MOESM1_ESM.pdf]

Supplementary Information

Solvent-Triggered Reconfiguration of Optical Physical Unclonable Functions

Ji Hoon Kim,[1,2,3] Jihee Kim,[2] Jung Gun Bae,[1] Junsik Choi,[2] Seongsu Cho,[3] Daeyeon Lee,[*3] Hyunsik Yoon,[*2] and Won Bo Lee[*1]

[1]*Department of Chemical and Biological Engineering, Institute of Chemical Processes, Seoul National University, 08826, Republic of Korea*

[2]*Department of Chemical and Biomolecular Engineering, Seoul National University of Science and Technology, Seoul, 01811, Republic of Korea*

[3]*Department of Chemical and Biomolecular Engineering, University of Pennsylvania, Philadelphia, Pennsylvania, 19104, USA*

*Corresponding authors

Daeyeon Lee: daeyeon@seas.upenn.edu

Won Bo Lee: wblee@snu.ac.kr

Hyunsik Yoon: hsyoon@seoultech.ac.kr

This Supplementary Information document includes:

Supplementary Notes 1 and 2

Supplementary Figures 1 to 18

Supplemental Notes & Figures

Supplementary Note 1:

Calculation of Hansen solubility parameters and solubility distance

The Hansen solubility distance R_a is calculated as

$$R_a = \sqrt{4(\delta_{d,1} - \delta_{d,2})^2 + (\delta_{p,1} - \delta_{p,2})^2 + (\delta_{h,1} - \delta_{h,2})^2}$$

where δ_d, δ_p, and δ_h denote the dispersive, polar, and hydrogen-bonding components, respectively. The Hansen solubility parameters of PEGDA were estimated using the van Krevelen group-contribution method, in which the molecular structure is decomposed into first- and second-order functional groups and their individual contributions are summed to obtain δ_d, δ_p, and δ_h.[1] Solvent parameters were taken from standard Hansen solubility parameter databases.[2]

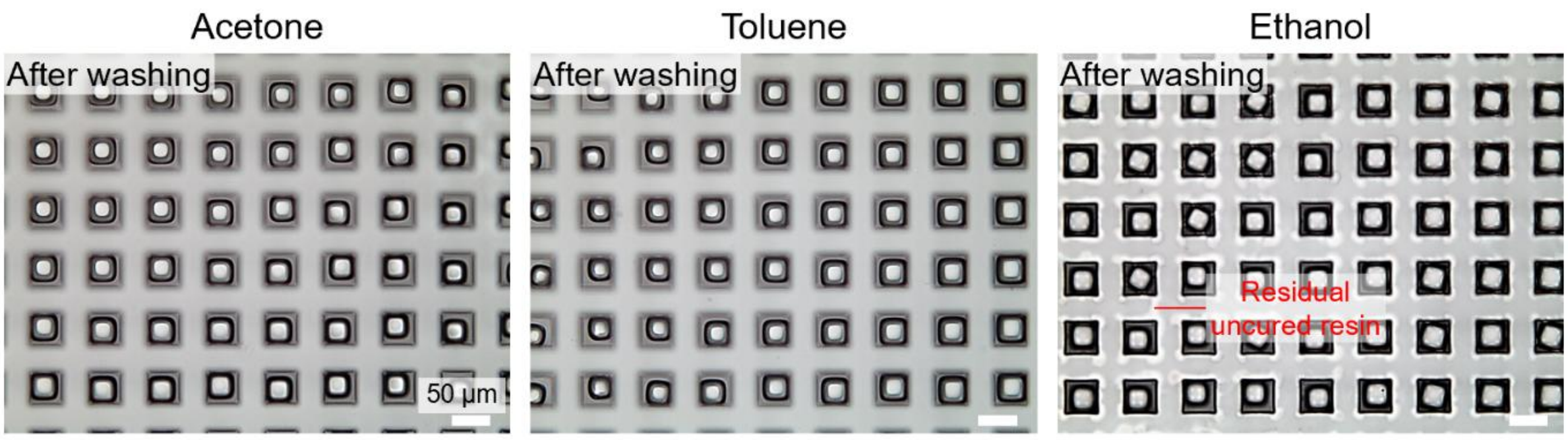

Material	δ_d $[MPa^{0.5}]$	δ_p $[MPa^{0.5}]$	δ_h $[MPa^{0.5}]$	R_a
PEGDA	17*	9*	5*	—
Acetone	15.5	10.4	7	3.9
Toluene	18	1.4	2	8.4
Ethanol	15.8	8.8	19.4	14.6

*Estimated using van Krevelen's group contribution method.

Supplementary Figure 1. Influence of solvent type on washing performance and its relation to Hansen solubility parameters.

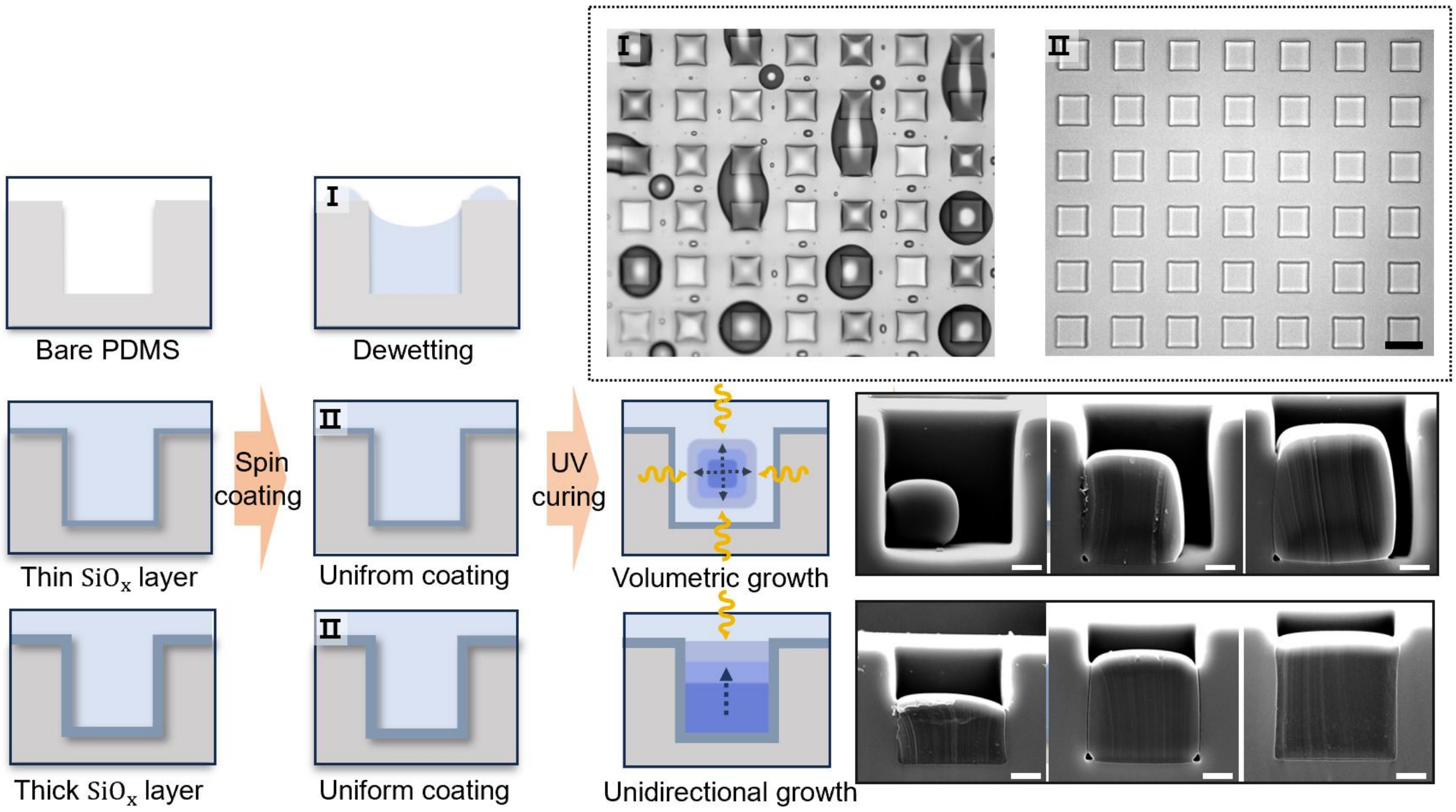

Supplementary Figure 2. Schematics and optical microscopy (OM) and SEM images illustrating the effect of plasma treatment on uniform coating formation and microparticle growth direction as a function of plasma treatment time (10 s for volumetric growth and 60 s for unidirectional growth).

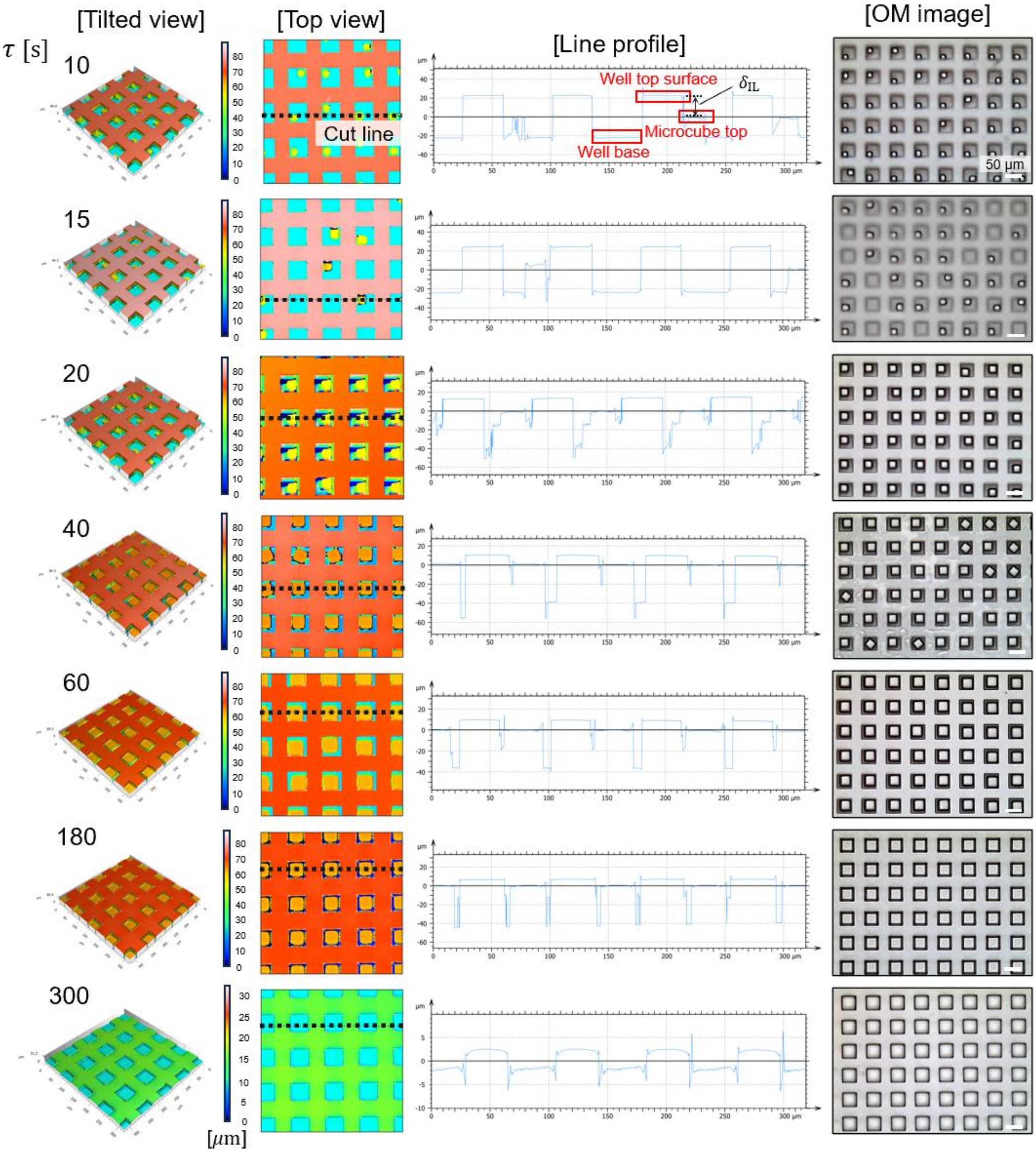

Supplementary Figure 3. Confocal and optical microscopy (OM) images capturing the volumetric growth of microcubes at varying curing times (τ).

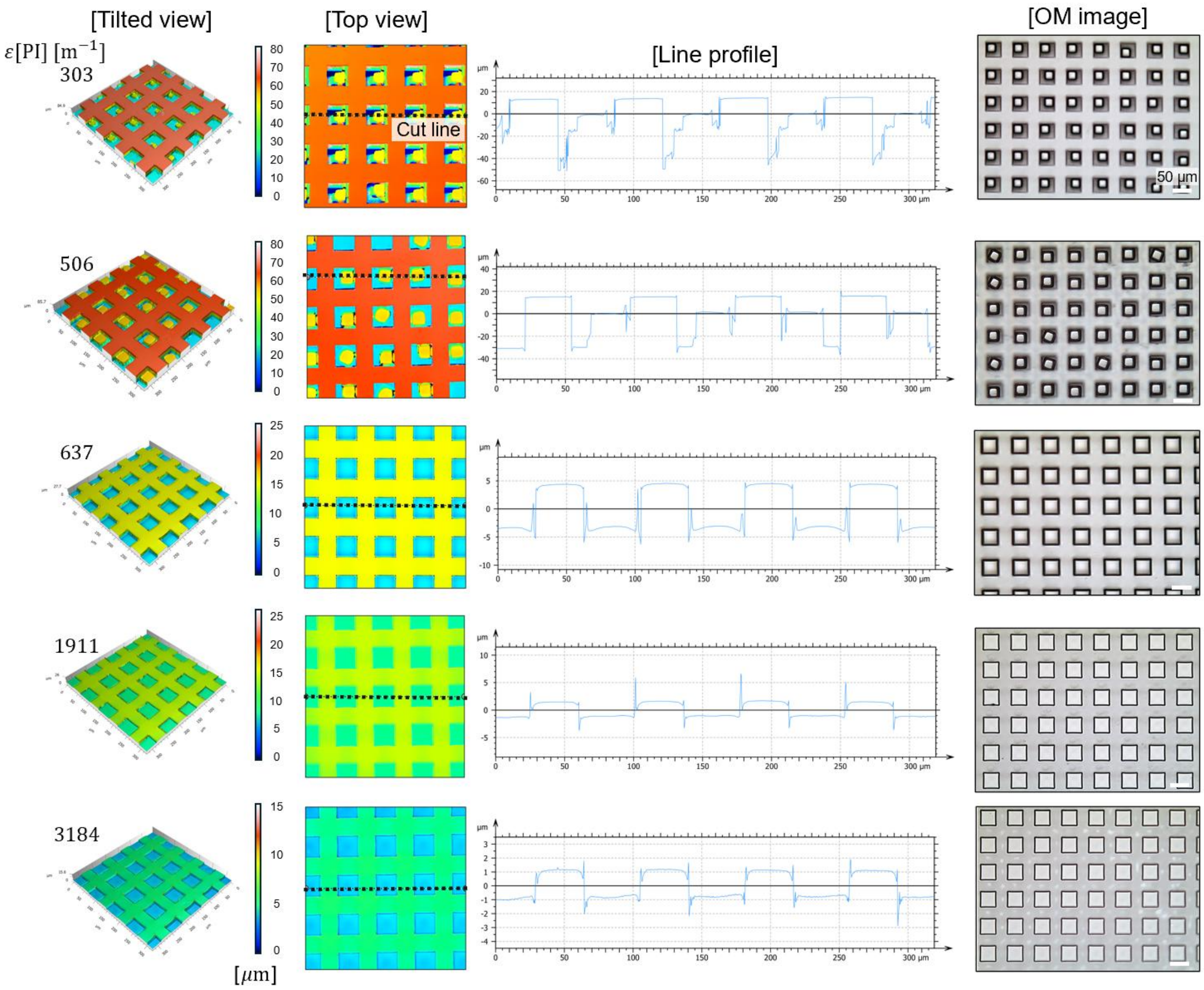

Supplementary Figure 4. Confocal and optical microscopy (OM) images illustrating the influence of photoinitiator conditions (ε[PI]) on microcube morphology.

Supplementary Note 2:

Modeling and finite element analysis of oxygen-inhibited photopolymerization in confined microwells

1. Nomenclature

Symbol	Description
PI	Photoinitiator
$\dot{\mathrm{R}}$	Primary radical
$\dot{\mathrm{M}}$	Propagating radical
O_2	Oxygen
RM_nOO	Propagating radical peroxide
$\dot{X}$	Lumped radical concentration (includes $\dot{\mathrm{R}}$, $\dot{\mathrm{M}}$)

2. Reaction mechanism

Step	Reaction	Description
Photolysis	$\mathrm{PI} \xrightarrow{h\nu} \dot{\mathrm{R}}$	Photoinitiator activation
Initiation	$\dot{\mathrm{R}} + \mathrm{M} \rightarrow \mathrm{R}\dot{\mathrm{M}}$	Chain initiation
Propagation	$\dot{\mathrm{R}}\mathrm{M_n} + \mathrm{M} \rightarrow \dot{\mathrm{R}}\mathrm{M_{n+1}}$	Chain propagation
Termination	$\dot{\mathrm{R}}\mathrm{M_n} + \dot{\mathrm{R}}\mathrm{M_m} \rightarrow \mathrm{RM_nM_M}$	Radical recombination
Inhibition	$\dot{\mathrm{R}}\mathrm{M_m} + \mathrm{O_2} \rightarrow \mathrm{RM_nOO}$	Radical quenching by oxygen

3. Kinetic model

Assuming quasi-steady-state conditions (i.e., rate of radical production equals rate of radical consumption), the lumped radical concentration $\dot{X}$ can be approximated as:[3]

$$\dot{X} = \frac{-k_{O_2}[O_2] + \sqrt{\left(k_{O_2}[O_2]\right)^2 + 4r_a k_t}}{2k_t}$$

where the radical production rate r_a is given by $r_a = \phi\, \epsilon\, I\, [PI]\, e^{-\epsilon[PI]z'}$ and z' denotes the vertical penetration depth from the top free surface.[3]

4. Species Balance Equations

$$\frac{\partial[O_2]}{\partial t} = D_{O_2}\frac{\partial^2[O_2]}{\partial z^2} - k_{O_2}[O_2]\dot{X}$$

$$\frac{\partial[M]}{\partial t} = -k_p[M]\dot{X}$$

5. Parameters used in simulation

Parameter	Description	Value	Unit	Source
k_p	Propagation rate constant	25	m³/(mol·s)	4
k_t	Termination rate constant	2520	m³/(mol·s)	4
k_{O_2}	Oxygen quenching rate constant	5×10^5	m³/(mol·s)	5
ϕ	Quantum yield (photoinitiator)	0.6	–	6
ϵ	Molar extinction coefficient (decadic)	1.6 for HMPP	m³/mol	6
		15 for DMPA		7
$I(\lambda)$	Light intensity (365 nm)	10	mW/cm²	Measured
$D_{O_2,PEGDA}$	Oxygen diffusion coefficient in PEGDA	2.84×10^{-11}	m²/s	8
$D_{O_2,PDMS}$	Oxygen diffusion coefficient in PDMS	3.25×10^{-9}	m²/s	9
D_{O_2,SiO_x}	Oxygen diffusion coefficient in SiO_x barrier	1.00×10^{-14}	m²/s	Estimated
$[O_2]_{eqb}$	Equilibrium oxygen concentration (free surface)	1.5	mol/m³	10
d_{SiO_x}	Thickness of SiO_x barrier	10	nm	Estimated
$[PI]$	Photoinitiator concentration	189	mol/m³	Measured
$[M]_0$	Initial monomer concentration	1600	mol/m³	Measured

**Napierian molar extinction coefficient ($\epsilon_{nat} \approx 2.303 \cdot \varepsilon$) is used for computation.*

Although direct measurement of d_{SiO_x} and D_{O_2,SiO_x}, under different plasma conditions is challenging, we estimated the oxygen diffusion coefficient in the silica-like barrier using a serial resistance model from previously reported effective diffusivity:

$$\frac{d_{tot}}{D_{O_2,eff}} = \frac{d_{SiO_x}}{D_{O_2,SiO_x}} + \frac{d_{PDMS}}{D_{O_2,PDMS}}$$

Here, the measured effective diffusivity is $D_{O_2,eff} \approx 1.0 \times 10^{-9}\ m^2/s$ and the PDMS thickness is $d_{PDMS} = 1\ mm$.[9] Assuming $d_{\mathrm{tot}} \approx d_{\mathrm{PDMS}}$ and adopting $d_{SiO_x} = 10\ nm$ based

on plasma conditions comparable to those described by Bertrand et al.,[11] the intrinsic diffusivity of the silica-like barrier is estimated as $D_{O_2,SiO_x} \approx 1.4 \times 10^{-14}\ m^2/s$.

6. Geometry

The domain consists of a periodic unit cell containing a cubic microwell (45 µm × 45 µm × 45 µm) with a lateral spacing of 30 µm between adjacent wells. The well is filled with a resin and an overlying resin layer with a thickness of 7.8 µm is present above the well.

7. Boundary and initial conditions

Boundary conditions

1) Dirichlet (liquid-air interface): $[O_2](z_{surf}, t) = [O_2]_{eqb}$

2) Periodic (lateral boundaries): $[O_2]_{src} = [O_2]_{dst}$, $[M]_{src} = [M]_{dst}$,

(‘src’ and ‘dst’: source and destination boundaries).

Initial conditions

$$[O_2](z,0) = [O_2]_{eqb}, \qquad [M](z,0) = [M]_0$$

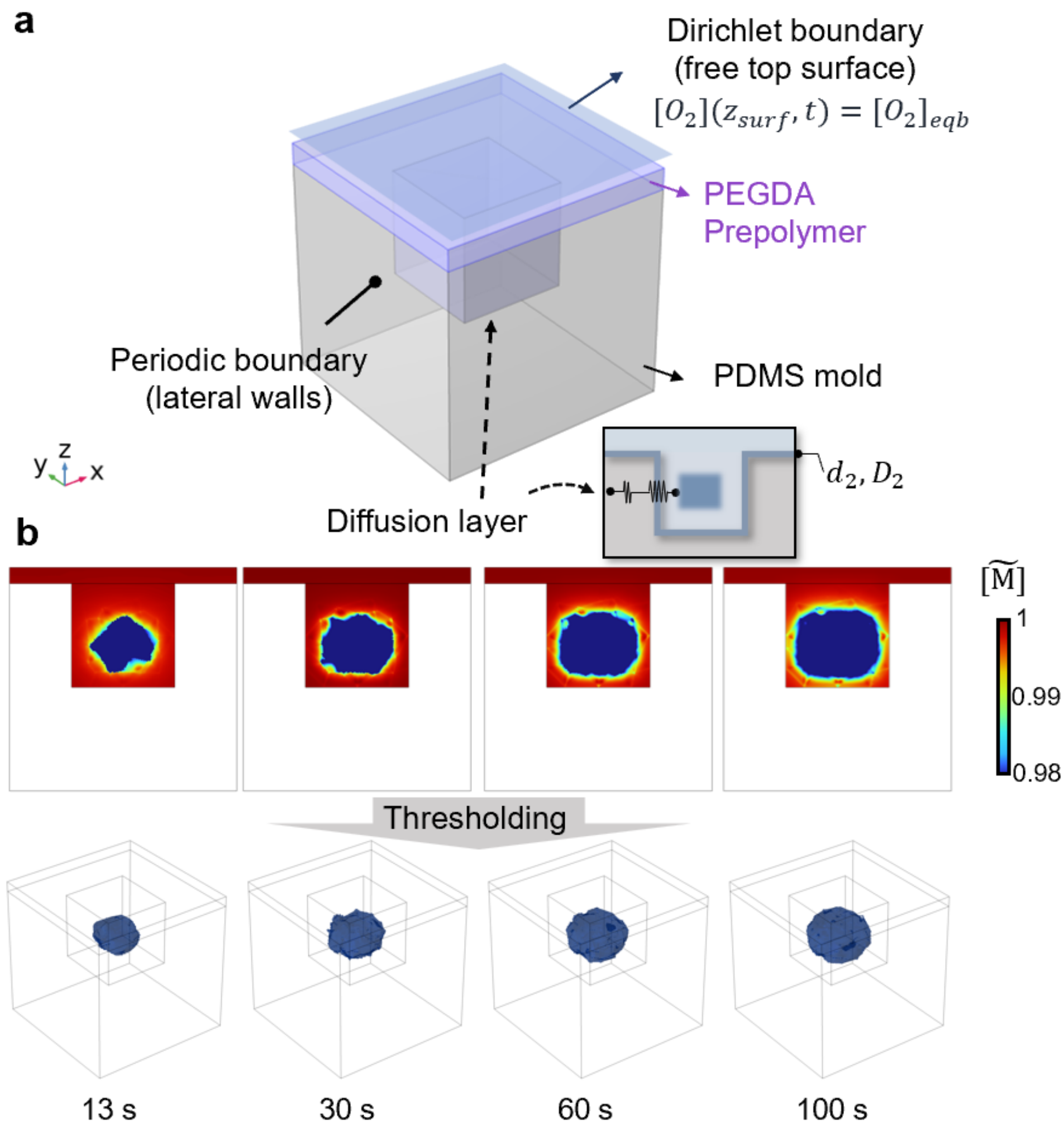

Supplementary Figure 5. Finite element simulation of oxygen-inhibited photopolymerization of PEGDA within a PDMS microwell.

(a) Simulation geometry and boundary conditions.

(b) Monomer concentration profiles at different curing times, with particle domains identified by thresholding ($[\widetilde{M}] < 0.985$), where $[\widetilde{M}] = [M]/[M]_0$.

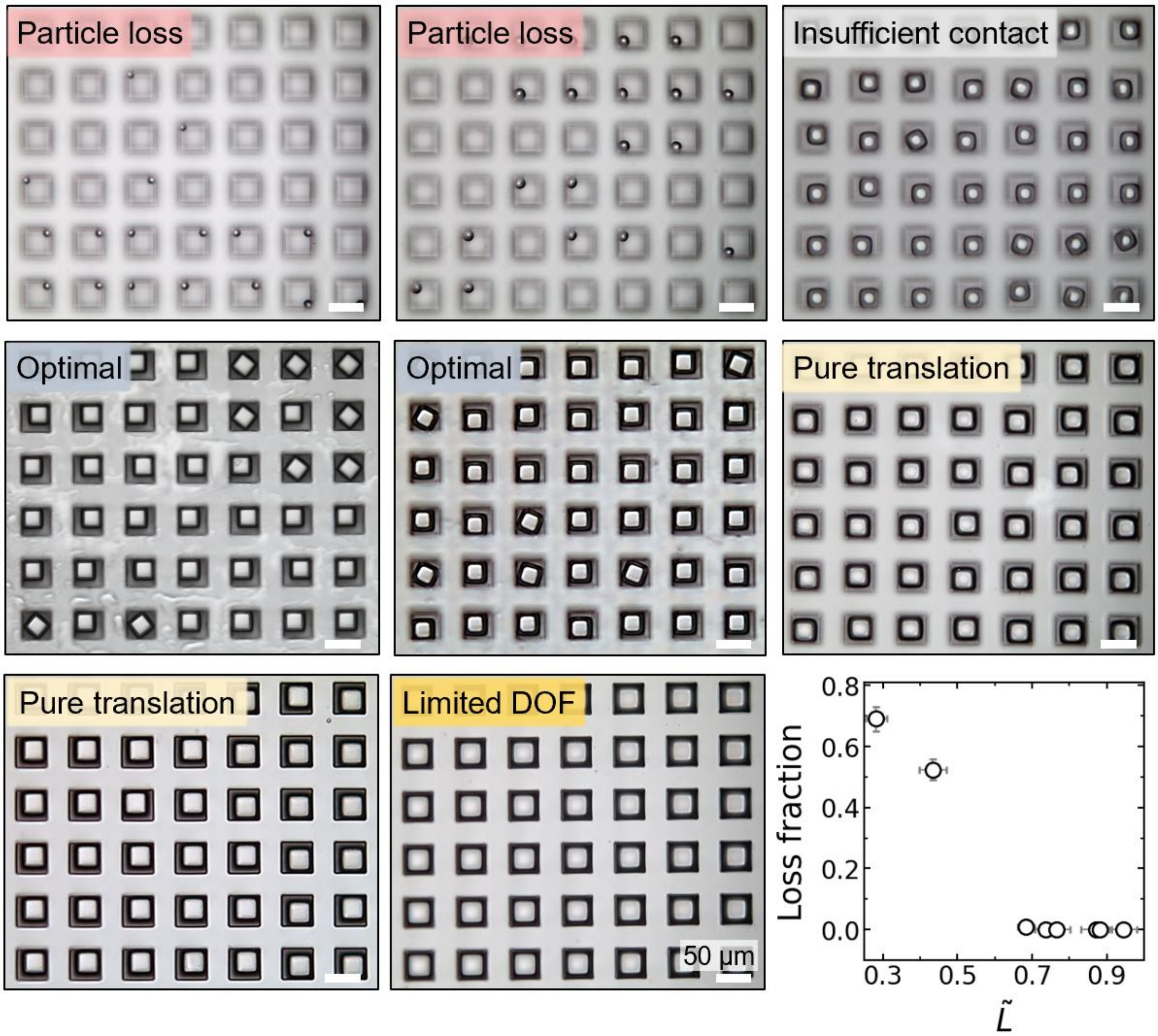

Supplementary Figure 6. Particle loss fraction and degree of freedom as a function of $\tilde{L}$.

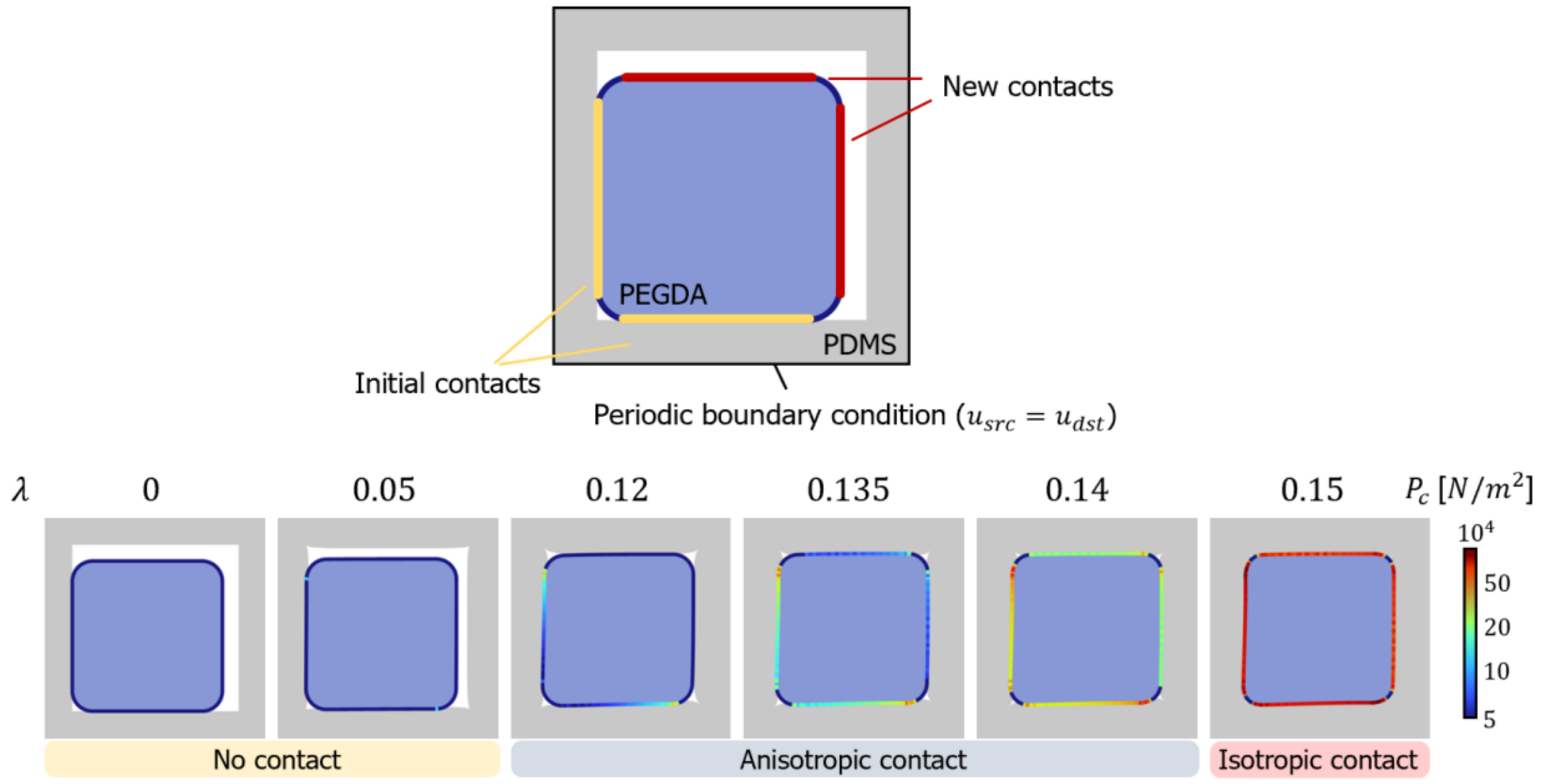

Supplementary Figure 7. Finite element simulation visualizing the evolution of contact pressure and the transition to isotropic contact with increasing swelling.

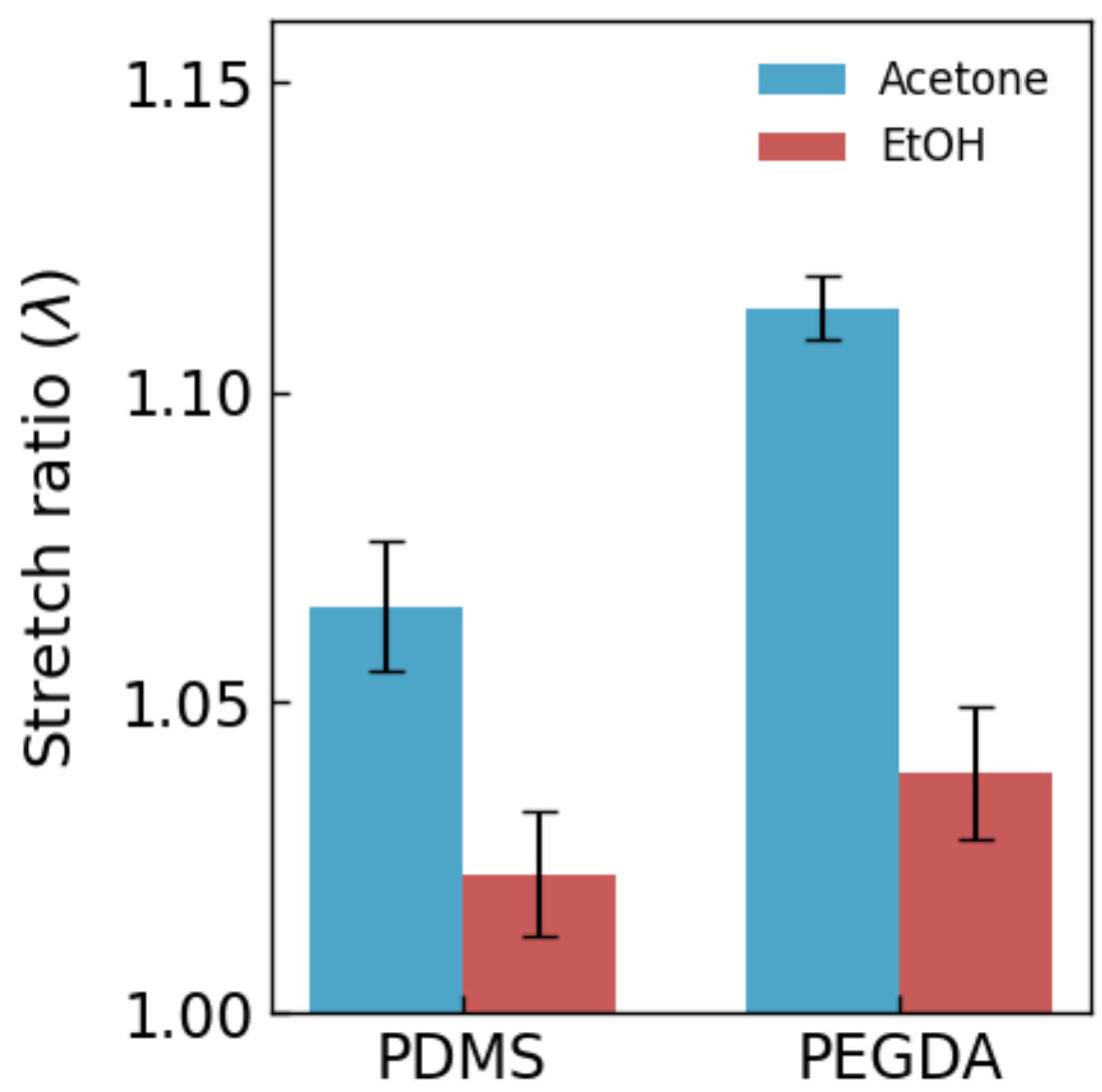

Supplementary Figure 8. Swelling ratios of PDMS and PEGDA in acetone and ethanol. Error bars denote standard deviation (n > 10).

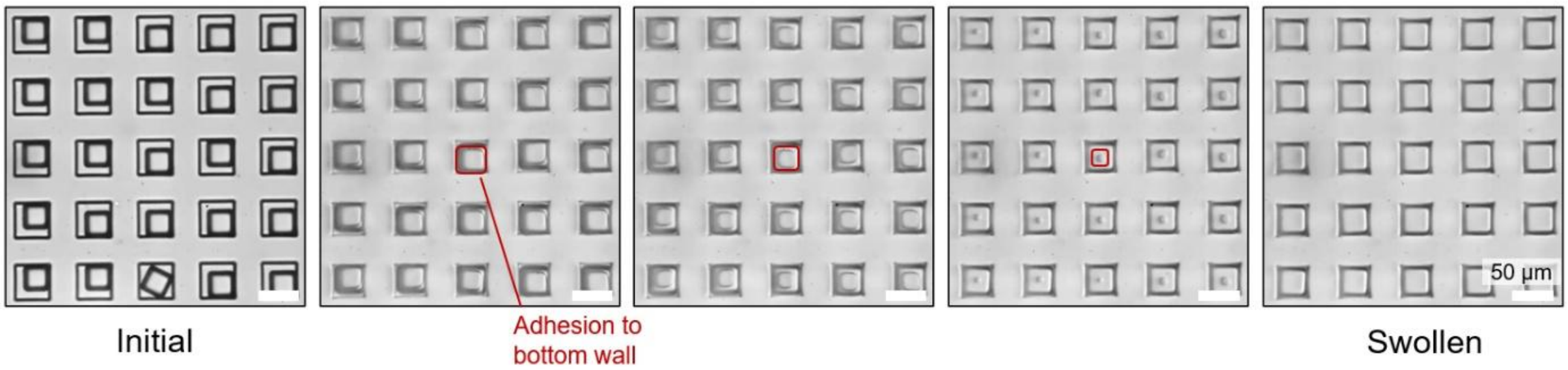

Supplementary Figure 9. Time-sequenced optical microscopy images showing rapid detachment of the microcubes from the bottom walls at the early stage of solvent exposure.

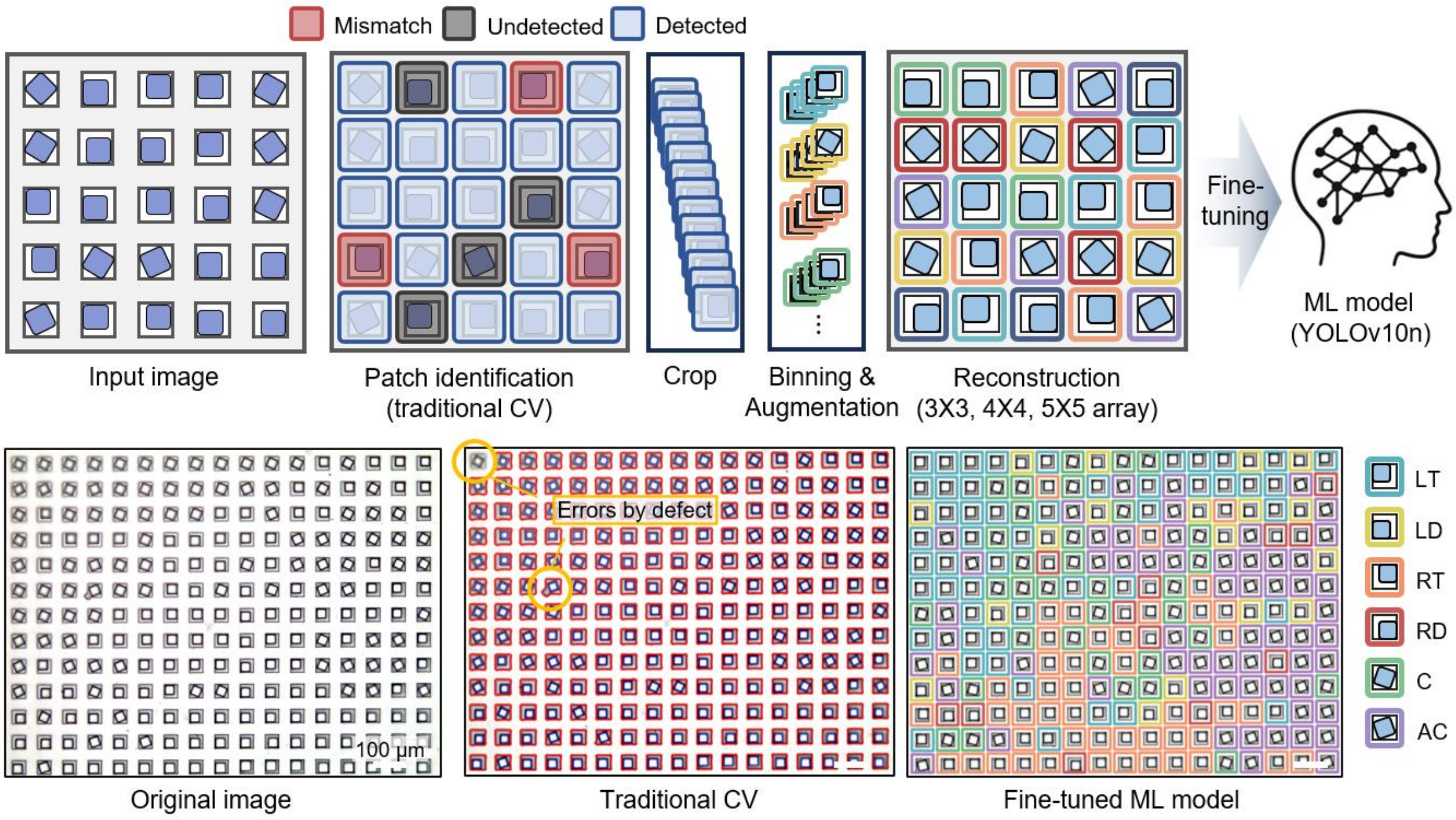

Supplementary Figure 10. Data preparation workflow for fine-tuning the object detection model and representative detection results.

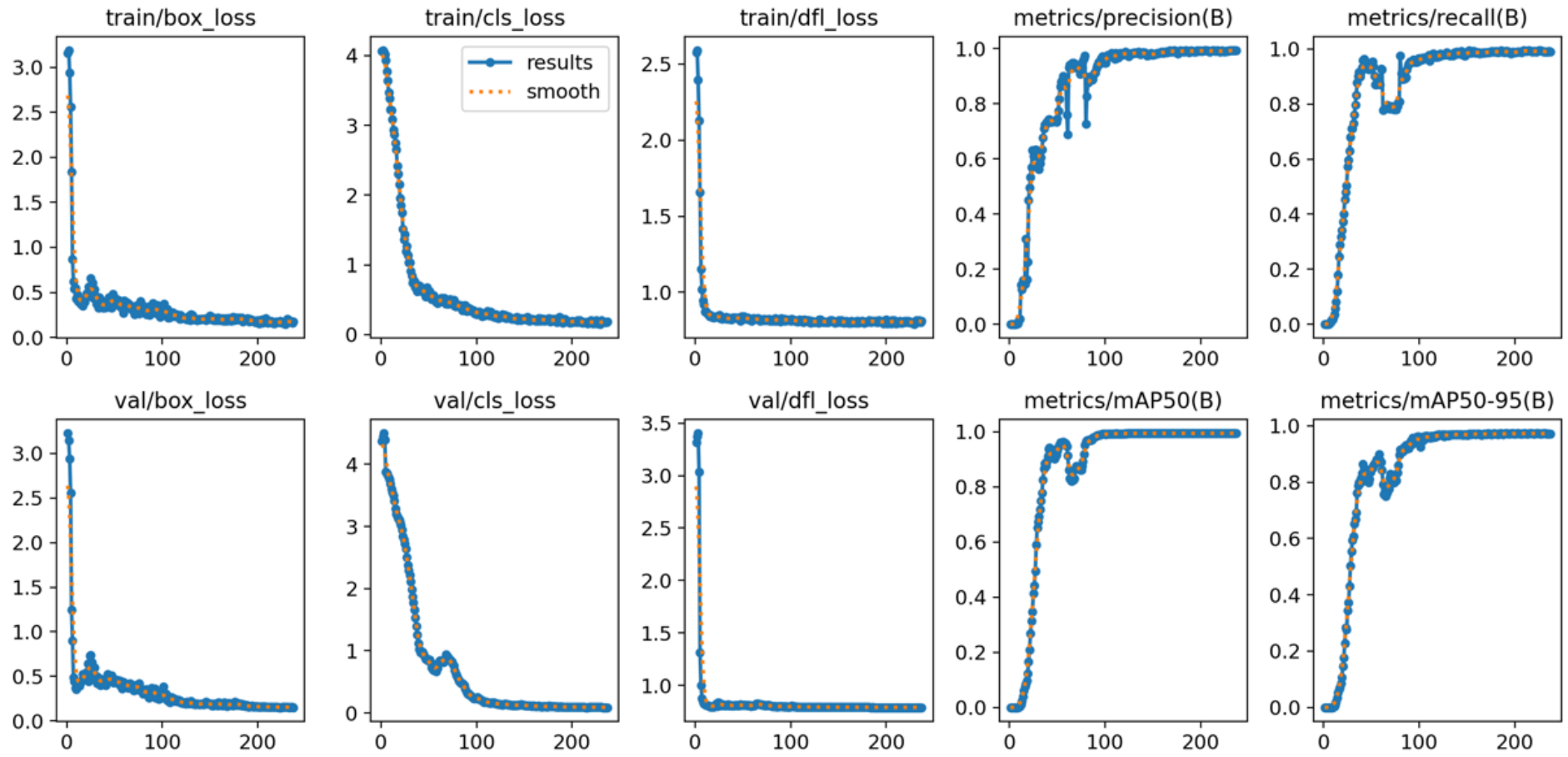

Metric	Percentage
mAP 50	99.0 %
mAP 50–95	98.8%
Precision	99.4%
Recall	97.4%

Supplementary Figure 11. Training and validation curves and final performance metrics of the fine-tuned object detection model (YOLOv10n).

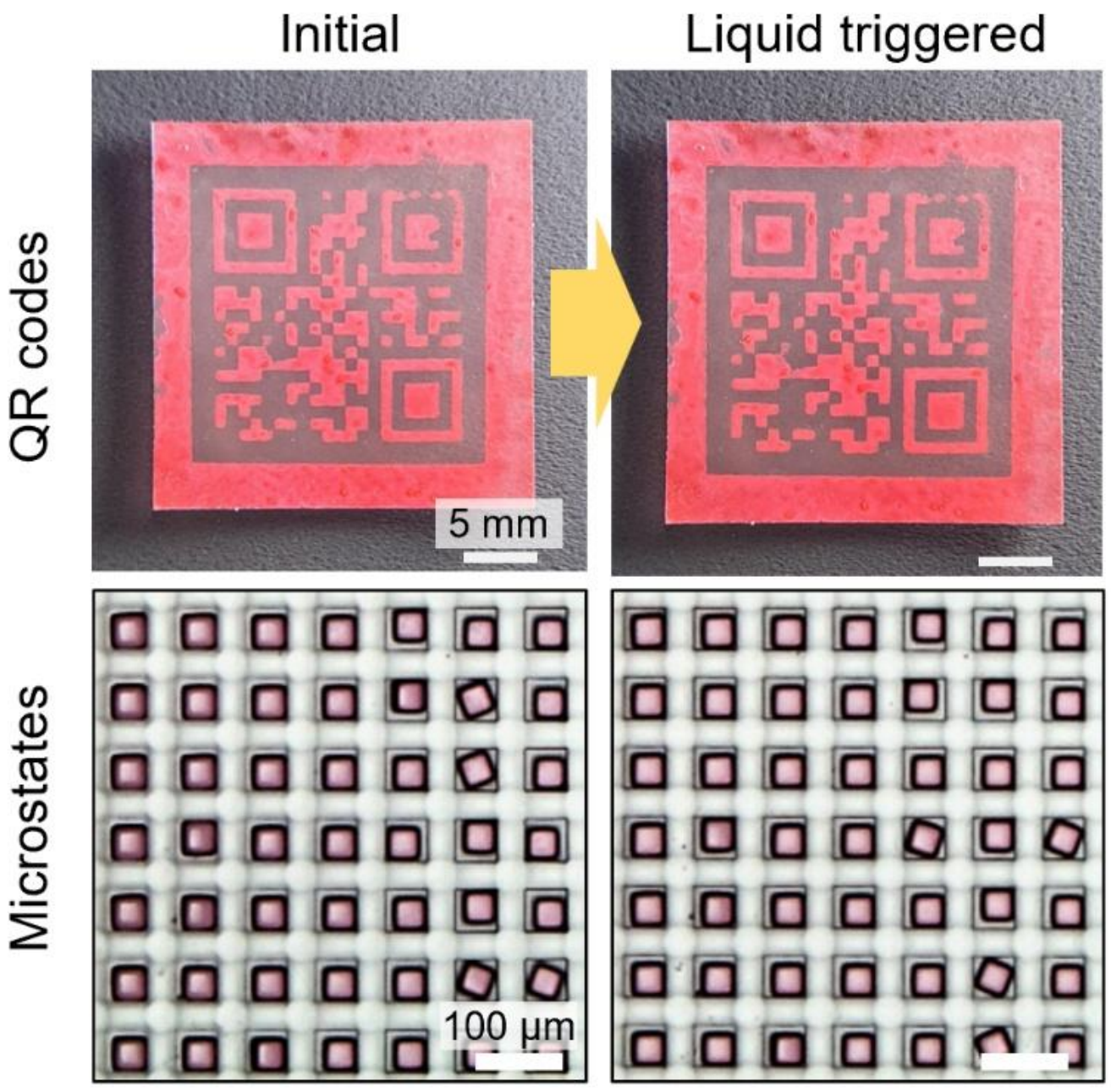

Supplementary Figure 12. QR code images before and after solvent-triggered reconfiguration, demonstrating preserved readability independent of the underlying PUF microstates. Optical microscopy images show the corresponding microstate changes.

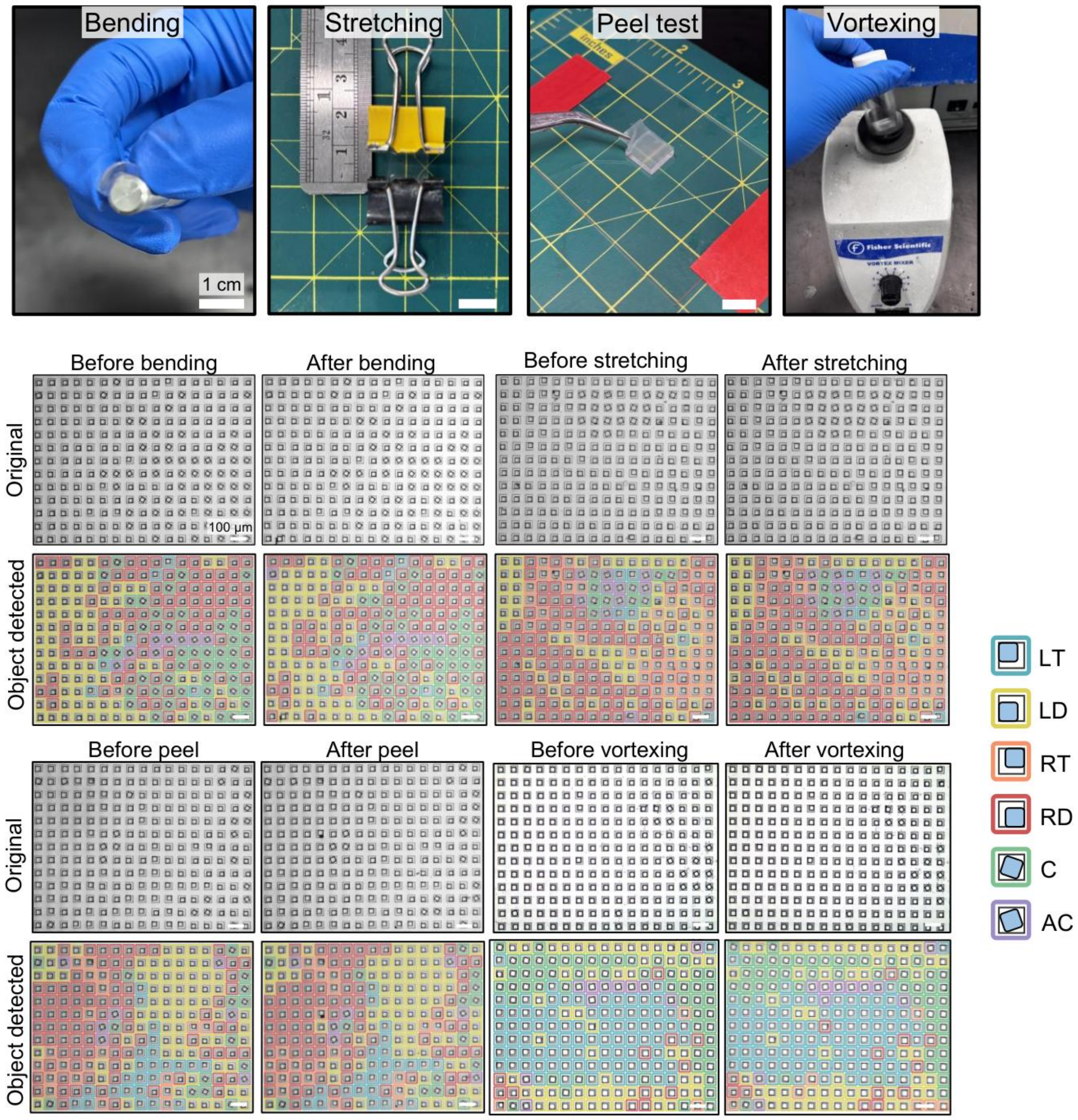

Supplementary Figure 13. Mechanical stability test of a PUF under bending, stretching, peeling and vortex-induced vibration. Mechanical test setups and optical microscopy images of the encoded patterns before and after testing, with the corresponding Hamming Distance (HD) calculated using a fine-tuned object detection model (YOLOv10n).

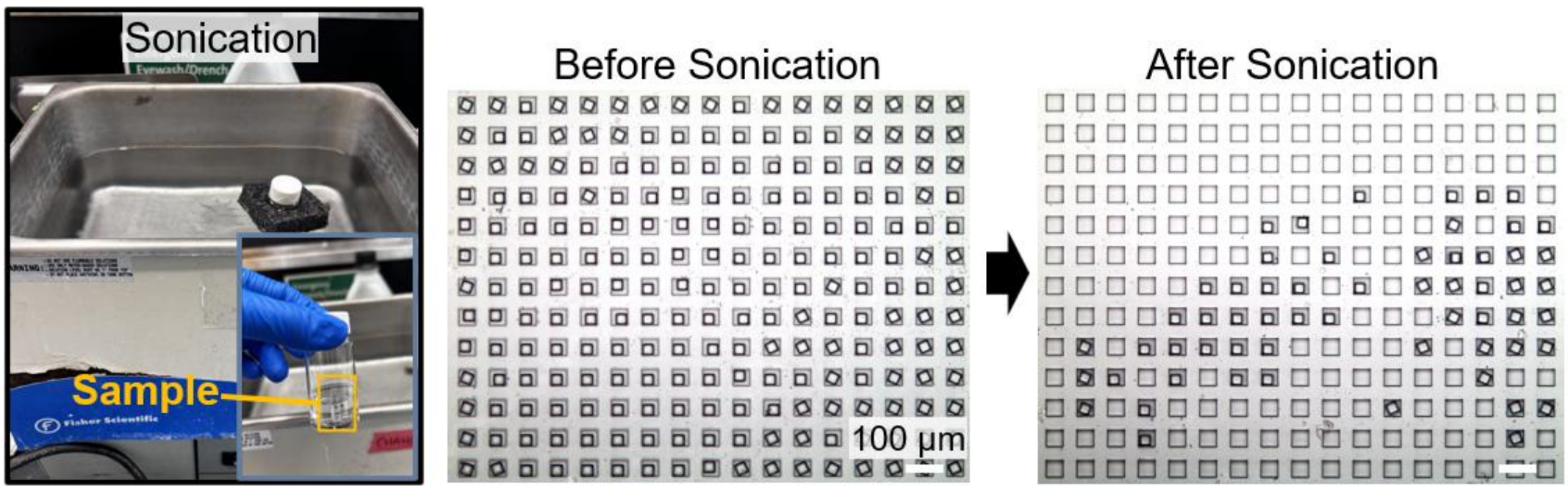

Supplementary Figure 14. Detachment of microcubes from microwells under bath sonication in aqueous media.

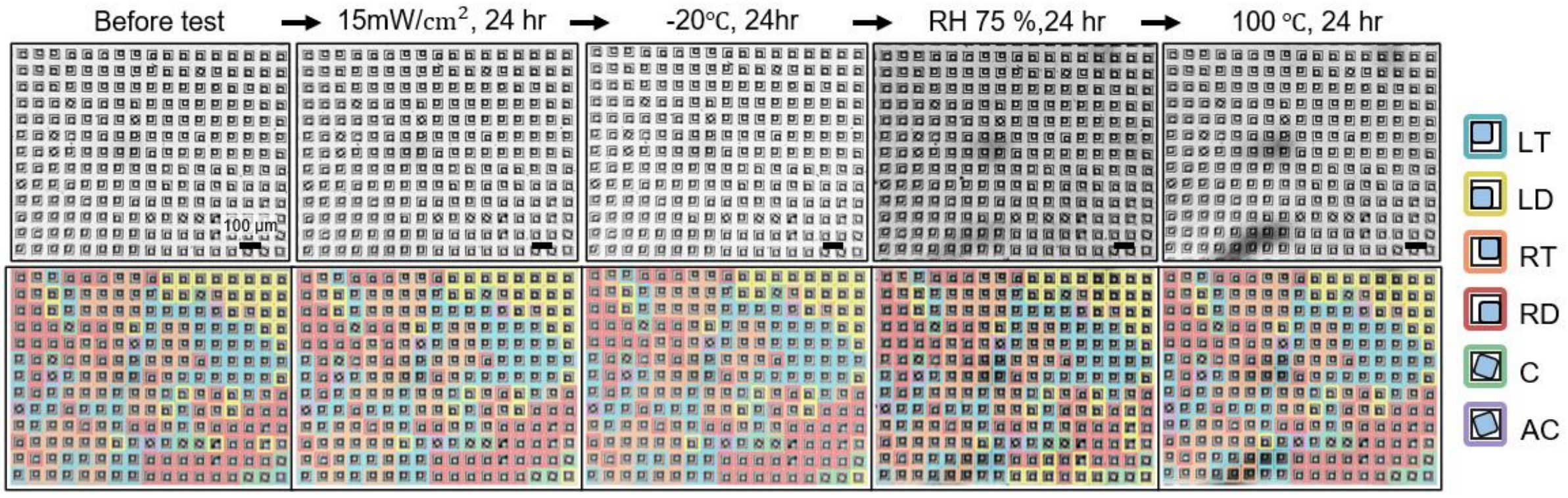

Supplementary Figure 15. Environmental stability test of a PUF under sequential UV exposure, freezing, high-humidity conditions, and thermal annealing applied to the same PUF code. The corresponding Hamming Distance (HD) was calculated using a fine-tuned object detection model (YOLOv10n).

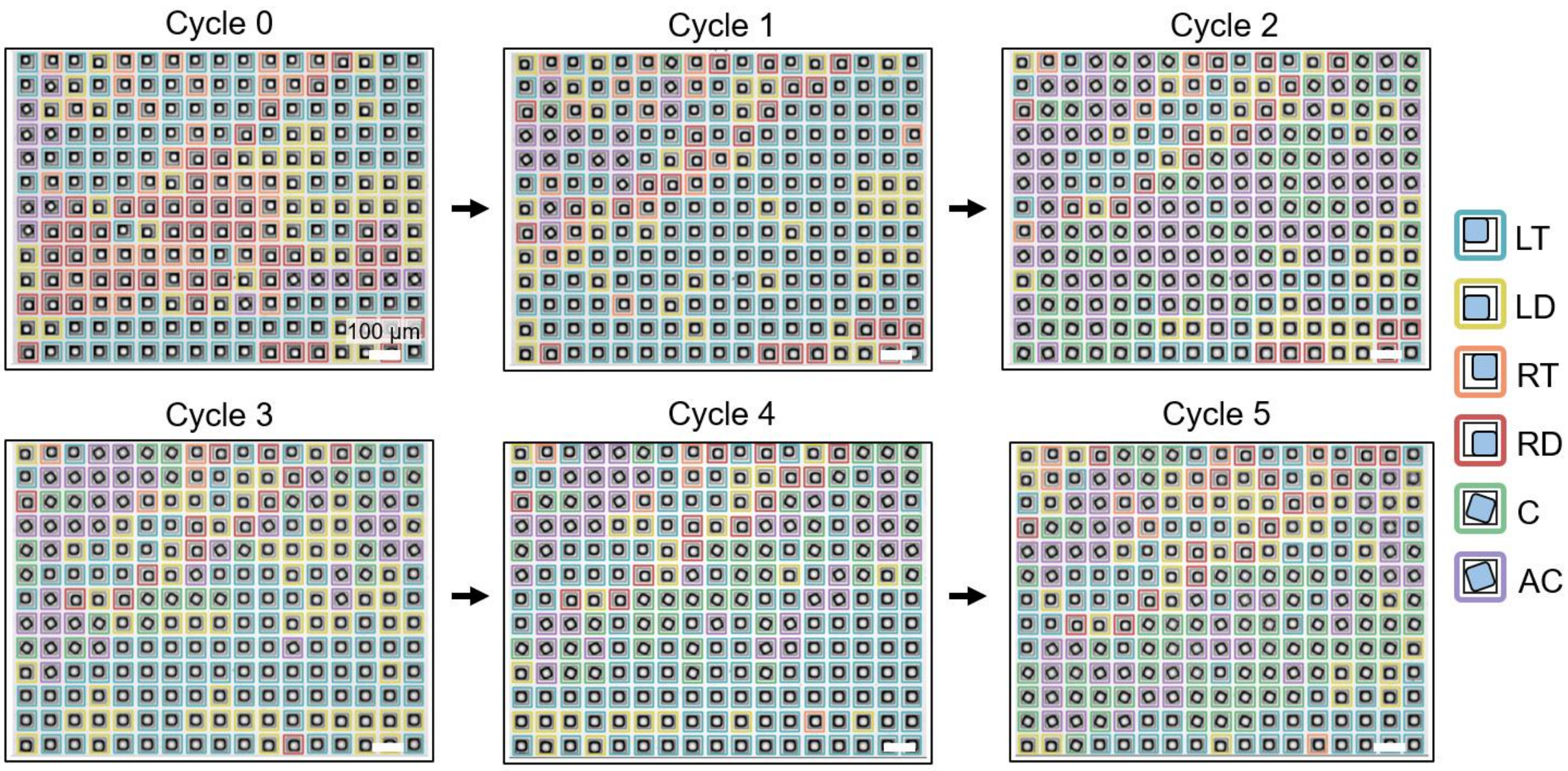

Supplementary Figure 16. Sequential reconfiguration of the PUF pattern over 5 repeated cycles.

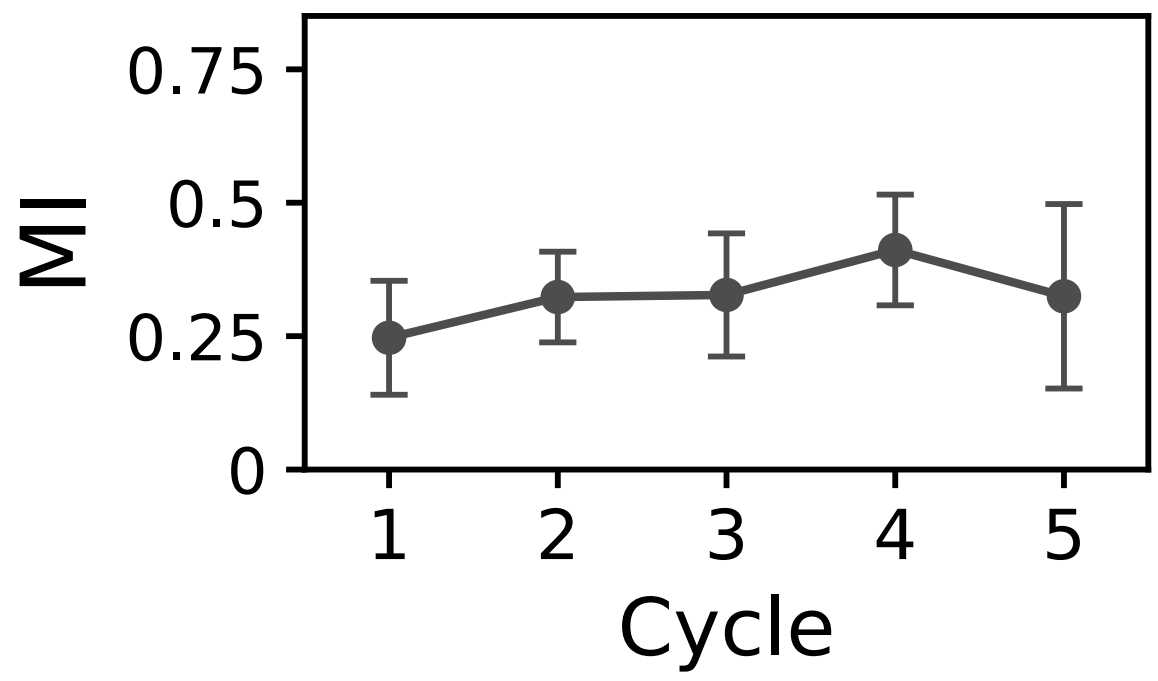

Supplementary Figure 17. Normalized mutual information between consecutive reconfiguration cycles over 5 repeated solvent-triggered cycles. Error bars denote standard deviation (n = 5).

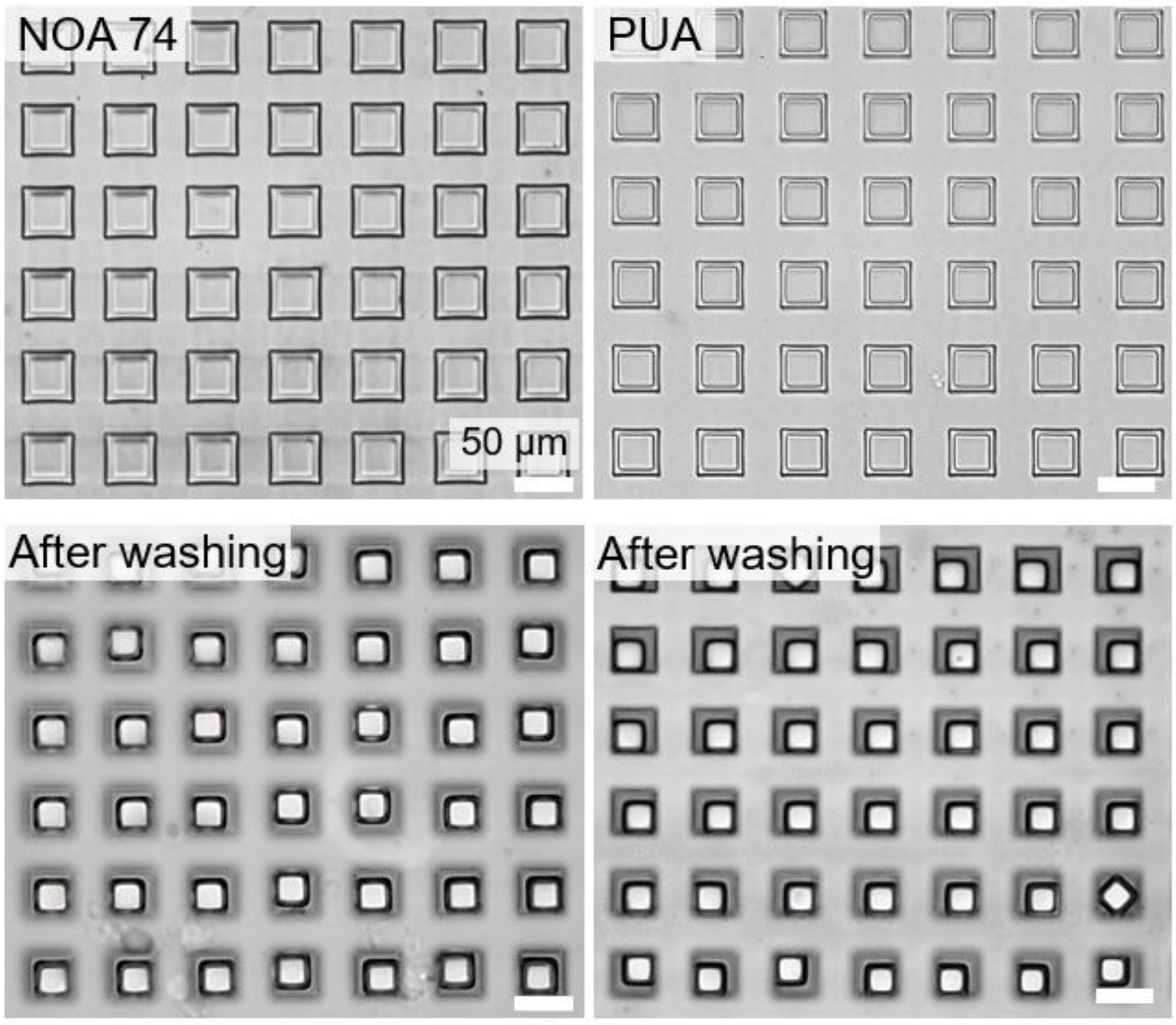

Supplementary Figure 18. Application of the oxygen-inhibition-based strategy to other acrylate-based systems (NOA 74 and PUA).

References

1. Stefanis, E. & Panayiotou, C. Prediction of Hansen Solubility Parameters with a New Group-Contribution Method. *Int. J. Thermophys.* **29**, 568–585 (2008).
2. Hansen, C. M. *Hansen Solubility Parameters: A User's Handbook, Second Edition*. (CRC Press, Boca Raton, 2007). doi:10.1201/9781420006834.
3. Dendukuri, D. *et al.* Modeling of oxygen-inhibited free radical photopolymerization in a PDMS microfluidic device. *Macromolecules* **41**, 8547–8556 (2008).
4. O'Brien, A. K. & Bowman, C. N. Modeling the Effect of Oxygen on Photopolymerization Kinetics. *Macromol. Theory Simul.* **15**, 176–182 (2006).
5. Decker, C. & Jenkins, A. D. Kinetic approach of oxygen inhibition in ultraviolet- and laser-induced polymerizations. *Macromolecules* **18**, 1241–1244 (1985).
6. Lecamp, L., Lebaudy, P., Youssef, B. & Bunel, C. Influence of UV radiation wavelength on conversion and temperature distribution profiles within dimethacrylate thick material during photopolymerization. *Polymer* **42**, 8541–8547 (2001).
7. Yamamoto, S. UV-curable pressure-sensitive adhesive composition and its pressure-sensitive adhesive sheet. (2001).
8. Lin, H. & Freeman, B. D. Gas Permeation and Diffusion in Cross-Linked Poly(ethylene glycol diacrylate). *Macromolecules* **39**, 3568–3580 (2006).
9. Markov, D. A., Lillie, E. M., Garbett, S. P. & McCawley, L. J. Variation in diffusion of gases through PDMS due to plasma surface treatment and storage conditions. *Biomed. Microdevices* **16**, 91–96 (2014).
10. Goodner, M. D. & Bowman, C. N. Development of a comprehensive free radical photopolymerization model incorporating heat and mass transfer effects in thick films. *Chem. Eng. Sci.* **57**, 887–900 (2002).
11. Béfahy, S. *et al.* Thickness and Elastic Modulus of Plasma Treated PDMS Silica-like Surface Layer. *Langmuir* **26**, 3372–3375 (2010).
