# Supplementary material for: Solvent-triggered reconfiguration of optical physical unclonable functions
Source: Nat Commun. 2026 Jun 20;17:7826. doi: 10.1038/s41467-026-74463-5 (PMC13438844; doi:10.1038/s41467-026-74463-5)
Supplement: Supplementary file 2 [file 41467_2026_74463_MOESM2_ESM.pdf]

Description of Additional supplementary files

Supplementary Movie 1. Rapid detachment of microcubes from the bottom walls upon solvent exposure.

Supplementary Movie 2. Reconfiguration mechanism during acetone deposition on a microcube array. As evaporation proceeds from the conformal contact state, uneven detachment of the lateral wall contacts drives microcubes to transition non-deterministically to new stable configurations.

Supplementary Movie 3. Three consecutive acetone deposition cycles on a microcube array.

Supplementary Movie 4. Three consecutive ethanol deposition cycles on a microcube array. Insufficient swelling in each cycle prevents contact with new lateral walls, resulting in particle immobilization without reconfiguration.

Supplementary Movie 5. Five consecutive solvent-triggered reconfiguration cycles of microcube arrays under repeated acetone deposition.
