# Supplementary material for: Solvent-triggered reconfiguration of optical physical unclonable functions
Source: Nat Commun. 2026 Jun 20;17:7826. doi: 10.1038/s41467-026-74463-5 (PMC13438844; doi:10.1038/s41467-026-74463-5)
Supplement: Supplementary file 3 [file 41467_2026_74463_MOESM8_ESM.pdf]

nature portfolio

Peer Review File

Solvent-Triggered Reconfiguration of Optical Physical Unclonable Functions

Corresponding Author: Professor Hyunsik Yoon

This file contains all reviewer reports in order by version, followed by all author rebuttals in order by version.

Version 0:

Reviewer comments:

Reviewer #1

(Remarks to the Author)
I enjoyed reading this manuscript. It presents a novel and very clever architecture for reconfigurable optical physical unclonable functions (r-PUFs) using polymeric microcube arrays confined within PDMS microwells. The work successfully decouples environmental stability from reconfigurability by employing a volatile solvent (acetone) as a specific trigger for stochastic rearrangement. By integrating in situ oxygen-inhibited photopolymerization for fabrication and a YOLOv10n AI framework for authentication, the authors demonstrate a high-capacity security platform (7221 states) that remains stable under diverse mechanical and thermal stresses. I believe this manuscript makes a significant and elegant contribution to the field of anti-counterfeiting.
I recommend the manuscript for publication with minor revisions.

1. The FEA contact simulations assume a Coulomb friction coefficient of 0.01, which appears very low. Can the authors briefly justify the choice of this value?
2. I am curious: for this technology to be deployed, does it require high-quality images, or are pictures taken with a smartphone good enough?
3. Line 215, there is an extra space after mm2.

Reviewer #2

(Remarks to the Author)
This paper details a method for producing tamper-resistant, configurable Optical Unclonable Functions (oPUFs). The concept relies on the formation of swellable microscopic polymeric blocks encased in a container made from an oxygen permeable polymer (here: polydimethylsiloxane, PDMS). These structures are generated in situ without the need of post-assembly. During manufacturing, the polymer is inhibited by oxygen permeation through the walls thus ensuring that the components remain mobile inside of the container.
The concept is interesting and the paper is very rich in experimental detail. The reviewer was particularly impressed by the detail given to the inhibition dynamics. As this is a method commonly applied in microfabrication, this would have not warranted this detail of investigation and a few references to established models would have sufficed. However, this is highly appreciated.
The reviewer would like to suggest two addition experiments to validate the usability of the process.
Firstly, it would be important to quantify the stability of the oPUF against mechanical tampering. As compression can be excluded by suitable setups, vibration is more challenging to isolate from. If the authors could verify, that the microblocks position cannot be changed by vibrations (including ultrasonic actuation) or to which degree the patterns will remain untampered, this would give the concept more strength. This would require only a subfigure as experimental result and a statement.
Secondly, the authors claim that the tag supports the full range of theoretical position. If this is the case, the process of generating each state should, in consequence, be of the same likeliness. This would mean, that the pattern formation is random, i.e., every new reset/set cycle, produces a new pattern. It would therefore be required to validate on a reasonable number of cycles, that no two pattern repeats. Given that the readout is automated to a high degree already using the YOLO model (which is a very solid choice), this should be reasonable to carry out. Every randomization-based identification requires pure randomness as input in order to prove that the system is not “physically skewed” - in a sense, has a tendency to produce only a subset of patterns.

After completing these two experiments, the reviewer would strongly support the publication of this paper!

Sincerely,
Bastian E. Rapp

Reviewer #3

(Remarks to the Author)
The authors present physical tags based on hydrogel microcubes embedded in polymer wells, with reconfigurability achieved via solvent immersion. The fabrication procedure is described in detail, including a careful selection of processing parameters. In addition, an AI-based authentication protocol is proposed to accelerate the identification process of the developed tags.
The manuscript is clear and well written. However, several fundamental aspects require deeper discussion and clarification.

1. A primary concern relates to the potential clonability of the system after fabrication. The tag consists of an ordered array of wells containing microcubes. The initial state appears to exhibit limited intrinsic unclonability: following optical and chemical inspection (e.g., Raman spectroscopy, nanoindentation), an adversary could potentially reproduce the tag using similar materials, geometry, and fabrication procedures. The authors are therefore encouraged to clarify how clonability is prevented in practice and to discuss the robustness of the proposed system against reverse-engineering strategies.
2. The novelty and relevance of the work in the field of reconfigurable PUFs are not sufficiently articulated. As presented by the authors, the literature already includes several reconfigurable PUF implementations, including magnetic-optical and memristive systems that demonstrate strong environmental stability. Moreover, existing works describe both weak PUFs (limited challenge–response pairs) and strong PUFs (large CRP spaces). The authors are invited to more clearly highlight the specific advancements of their approach compared to the current state of the art. In addition, the claimed applications in "pharmaceuticals" (page 4, line 68) and "medical products" (Discussion, line 280) require further justification, as the practical advantage of solvent-based reconfiguration in these contexts is not evident. For completeness, the comparison table of optical reconfigurable PUFs could be expanded to include recent demonstrations such as the reconfigurable PDMS–LC composite reported in APL Photonics, 11(2), 2026.

The characterization of the proposed PUF requires further quantitative analysis.

3. How has the encoding capacity been calculated? The encoding capacity should be calculated based on the number of independent bits in the extracted key, and not taking into account the total bit length (number of cubes per image) of the response string. The authors should therefore estimate the effective number of independent configurational states.
4. To demonstrate the security of the system, all possible microcube arrangements should ideally be equiprobable. However, from Figure 3c–e, corner states appear more likely than others, suggesting possible bias in the configurational distribution. The authors should provide a statistical analysis of the state uniformity. Furthermore, for the reconfiguration process to be unpredictable, successive states should be statistically independent of previous configurations. The authors should therefore quantify the correlation coefficient between states before and after reconfiguration (e.g., Figures S12–S16) and analyze how this correlation evolves over multiple reconfiguration cycles. If state equiprobability is not verified, the implications for security—particularly under repeated reconfiguration—should be discussed, including vulnerability to modeling or prediction attacks.
5. The definition of generalized fractional Hamming distance (FHD) for a base of seven states requires a more rigorous and formal derivation. The generalized uniqueness metric presented appears to evaluate the randomness across cube states rather than the independence of the tag before and after reconfiguration. This distinction should be clarified. Additionally, the expected theoretical uniqueness value for a 7-state-base PUF should be explicitly derived and compared with experimental results.
6. Regarding Figure 4f, clarification is needed on how correlation coefficients were calculated. Based on the optical images presented in both the main text and the supplementary material, the correlation coefficients computed over the entire image are expected to be close to 1. The authors should therefore specify whether image processing, or feature extraction steps were applied, and whether correlation was calculated globally or on segmented regions.

Version 1:

Reviewer comments:

Reviewer #1

(Remarks to the Author)
The authors have addressed all my comments. I recommend publication.

Reviewer #2

(Remarks to the Author)
The authors have done an excellent job revising the paper which is now ready for publication.

Reviewer #3

(Remarks to the Author)
The authors have addressed the reviewers' concerns by providing additional experiments and clarifications, significantly improving the manuscript. However, the proposed PUF does not demonstrate fully solvent reconfigurability.

In particular, although the authors revised the PUF state definition from a 7-ary to a 6-ary system and showed improvements in key PUF metrics such as bit uniformity and unclonability, the achieved reconfiguration performance remains not optimal because of the presence of residual correlations among the different responses. The stochasticity of the reconfiguration process reported in the article is not supported by the data analysis. The authors are invited to better discuss or revise this point.

Open Access This Peer Review File is licensed under a Creative Commons Attribution 4.0 International License, which permits use, sharing, adaptation, distribution and reproduction in any medium or format, as long as you give appropriate credit to the original author(s) and the source, provide a link to the Creative Commons license, and indicate if changes were made.
In cases where reviewers are anonymous, credit should be given to 'Anonymous Referee' and the source.
The images or other third party material in this Peer Review File are included in the article's Creative Commons license, unless indicated otherwise in a credit line to the material. If material is not included in the article's Creative Commons license and your intended use is not permitted by statutory regulation or exceeds the permitted use, you will need to obtain permission directly from the copyright holder.
To view a copy of this license, visit https://creativecommons.org/licenses/by/4.0/

Notes on revision made to manuscript NCOMMS-26-015112:

Solvent-Triggered Stochastic Reconfiguration of Optical Physical Unclonable Functions

Ji Hoon Kim, [1,2,3] Jihee Kim, [2] Jung Gun Bae, [1] Junsik Choi, [2] Seongsu Cho, [3] Daeyeon Lee, [*3] Hyunsik Yoon, [*2] and Won Bo Lee [*1]

[1]Department of Chemical and Biological Engineering, Institute of Chemical Processes, Seoul National University, 08826, Republic of Korea
[2]Department of Chemical and Biomolecular Engineering, Seoul National University of Science and Technology, Seoul, 01811, Republic of Korea
[3]Department of Chemical and Biomolecular Engineering, University of Pennsylvania, Philadelphia, Pennsylvania, 19104, USA

*Corresponding authors

Daeyeon Lee: daeyeon@seas.upenn.edu
Hyunsik Yoon: hsyoon@seoultech.ac.kr
Won Bo Lee: wblee@snu.ac.kr

Point-by-point response to reviewer's comments

Reviewer #1

Overall comment
I enjoyed reading this manuscript. It presents a novel and very clever architecture for reconfigurable optical physical unclonable functions (r-PUFs) using polymeric microcube arrays confined within PDMS microwells. The work successfully decouples environmental stability from reconfigurability by employing a volatile solvent (acetone) as a specific trigger for stochastic rearrangement. By integrating in situ oxygen-inhibited photopolymerization for fabrication and a YOLOv10n AI framework for authentication, the authors demonstrate a high-capacity security platform (7^221 states) that remains stable under diverse mechanical and thermal stresses. I believe this manuscript makes a significant and elegant contribution to the field of anti-counterfeiting.
I recommend the manuscript for publication with minor revisions.

[Author reply]
We sincerely appreciate the reviewer's positive assessment, and the time and effort devoted to reviewing our work. We have carefully addressed all suggestions to improve the manuscript.

1. The FEA contact simulations assume a Coulomb friction coefficient of 0.01, which appears very low. Can the authors briefly justify the choice of this value?

[Author reply]
We thank the reviewer for this helpful comment. The friction coefficient of μ = 0.01 was chosen to approximate the transient, solvent-lubricated PDMS–hydrogel contact during acetone exposure, rather than the dry PDMS–PEGDA interface in the final state. During solvent uptake, the interface behaves as a swollen, lubricated polymer contact, where interfacial sliding resistance is substantially reduced. As reported by Cuccia et al. :[1]

> [*PNAS,* 117.21 (2020): 11247-11256.]
> *"Generally, the friction coefficients (μ) of hydrogel–hydrogel and hydrogel–solid interfaces are very low, often below μ = 0.01 at laboratory-scale sliding velocities. Such low friction coefficients result partially from a hydrogel's high solvent volume fraction and associated lubrication."*

We therefore consider μ = 0.01 a physically reasonable estimate for the transient contact conditions modeled in our simulation.
To clarify these points, we have added the following highlighted sentence to the supplementary information:

(Page S6, Methods Section)
Frictional interactions were described by a Coulomb friction model with a friction coefficient of 0.01, chosen to approximate the transient, solvent-lubricated polymer contact conditions during acetone-induced swelling.[1] Material properties for PDMS were taken from the COMSOL material library, while those for PEGDA were obtained from literature sources.[2]

2. I am curious: for this technology to be deployed, does it require high-quality images, or are pictures taken with a smartphone good enough?

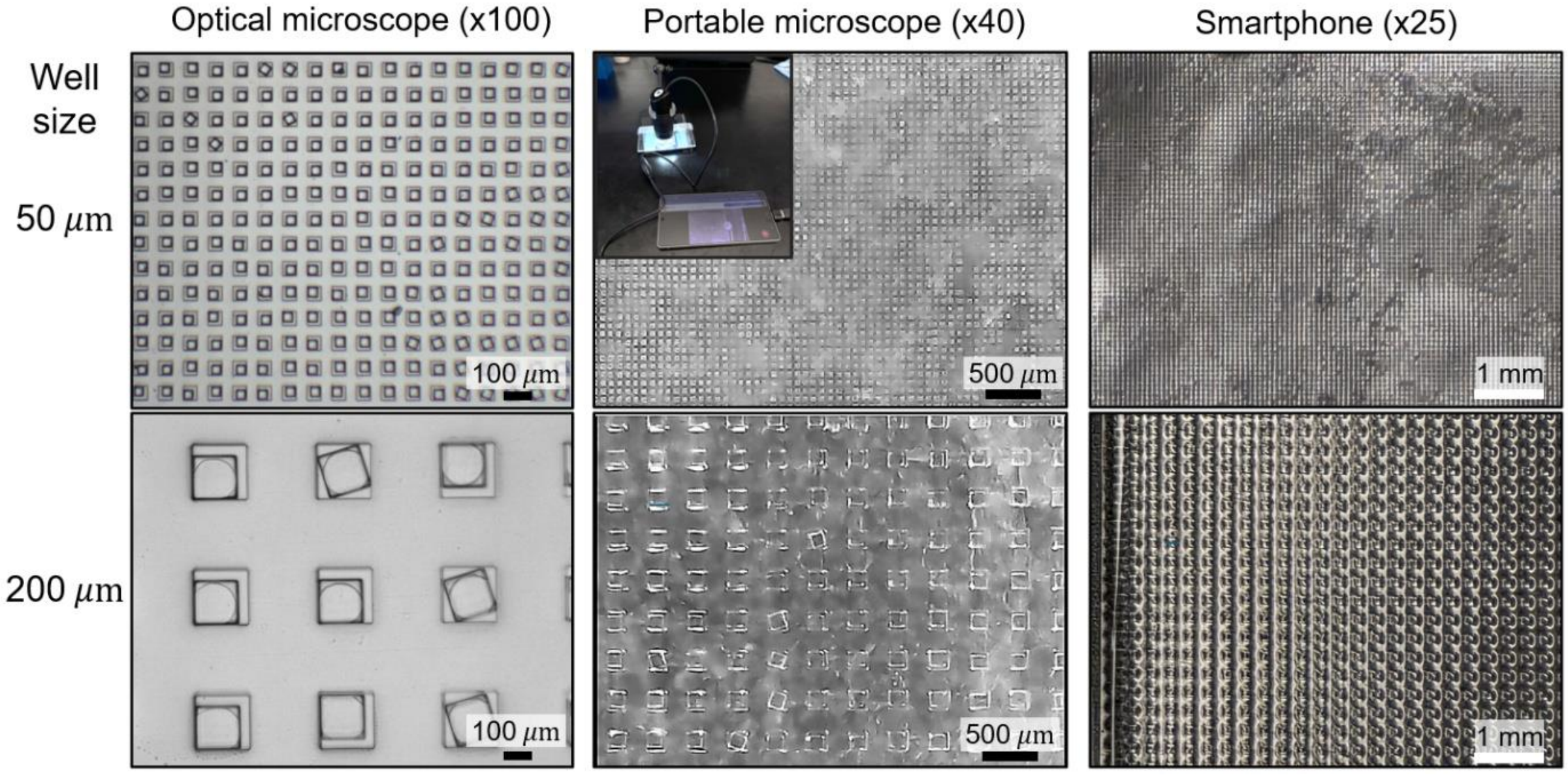

Figure R1. Comparison of PUF readout across imaging modalities at different feature sizes.

[Author reply]

We compared three imaging methods: an optical microscope (Olympus BX41M, ×100), a USB portable microscope (Smartoy, USD 55, ×40), and a smartphone camera (Samsung Galaxy S25 Ultra, ×25). For the microwell size used in this study (45 μm), images obtained with the portable microscope and smartphone are insufficient for reliable authentication (Figure R1), as the microcube configurations are not clearly resolved at this scale.

To further examine the usable range, we fabricated larger particle-in-well arrays consisting of 160 μm particles in 200 μm wells. Despite the continued limitations of the smartphone camera, the configurations are readily distinguishable with the portable microscope, enabling authentication when combined with image processing or AI-based analysis.

These results indicate that imaging requirements are primarily governed by feature size, suggesting that structures at the hundreds-of-micrometers scale are suitable for practical field authentication.

3. Line 215, there is an extra space after mm2.

[Author reply]

We thank the reviewer for the careful review. The typographical error has been corrected in the revised manuscript.

Reviewer #2

Overall comment

This paper details a method for producing tamper-resistant, configurable Optical Unclonable Functions (oPUFs). The concept relies on the formation of swellable microscopic polymeric blocks encased in a container made from an oxygen permeable polymer (here: polydimethylsiloxane, PDMS). These structures are generated in situ without the need of post-assembly. During manufacturing, the polymer is inhibited by oxygen permeation through the walls thus ensuring that the components remain mobile inside of the container.

The concept is interesting and the paper is very rich in experimental detail. The reviewer was particularly impressed by the detail given to the inhibition dynamics. As this is a method commonly applied in microfabrication, this would have not warranted this detail of investigation and a few references to established models would have sufficed. However, this is highly appreciated.

The reviewer would like to suggest two addition experiments to validate the usability of the process.

[Author reply]

We appreciate the reviewer's careful reading and insightful suggestions to strengthen our manuscript. We agree that validation of vibration resistance and repeated-cycle stochasticity would further strengthen the work, and we have addressed both points below.

During this revision, we also refined the state definition from the nominal 7-ary system to a practically relevant 6-ary system, as discussed in detail in our response to Comment 2.

1. It would be important to quantify the stability of the oPUF against mechanical tampering. As compression can be excluded by suitable setups, vibration is more challenging to isolate from. If the authors could verify, that the microblocks position cannot be changed by vibrations (including ultrasonic actuation) or to which degree the patterns will remain untampered, this would give the concept more strength. This would require only a subfigure as experimental result and a statement.

[Author reply]

We thank the reviewer for this valuable suggestion. We agree that vibration represents a more practically relevant mechanical perturbation during routine handling and distribution. To address this concern, we performed two representative vibration tests: (i) bath sonication in water (50–60 Hz, 30 min) and (ii) vortexing (~2000 rpm, 30 min).

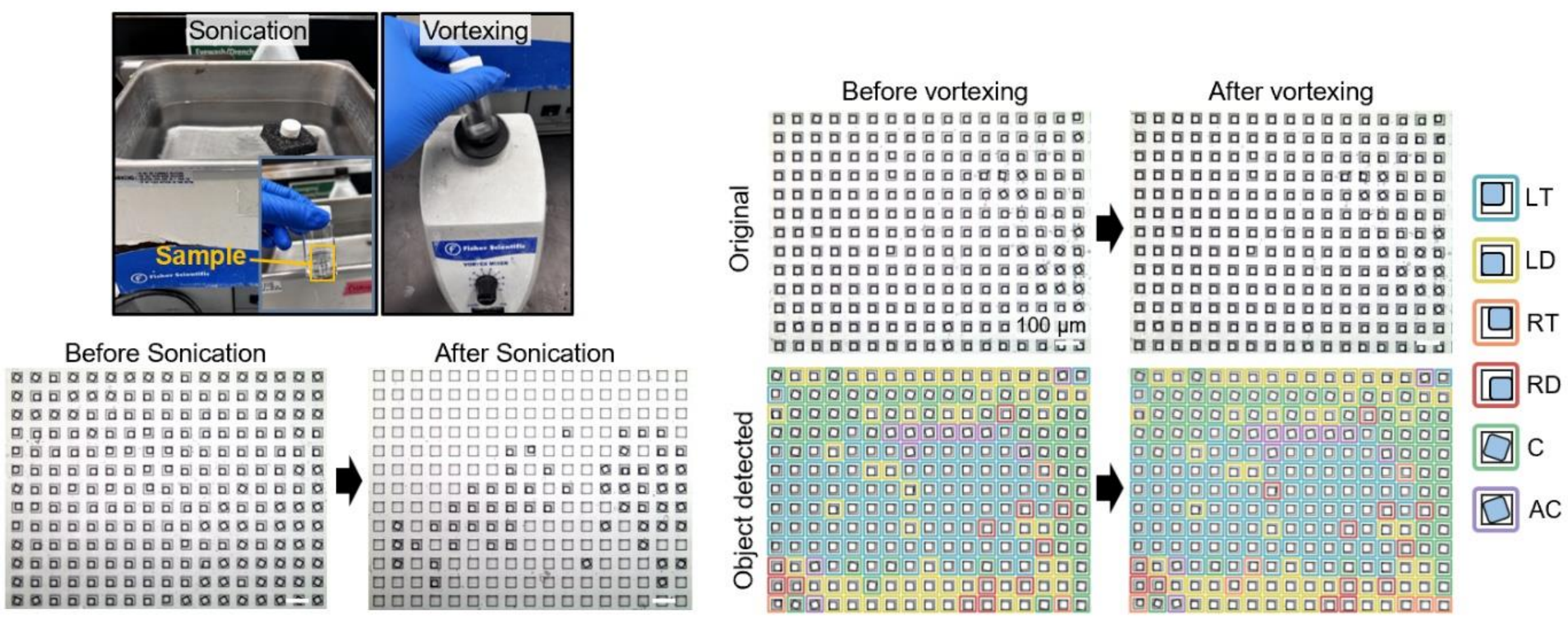

Figure R2. Vibration stability of the PUF under sonication and vortexing.

As shown in Fig. R2, the encoded patterns remained robust under vortexing (HD ~ 0.02), whereas most of the encoded particles detached after bath sonication. This suggests that sonication disrupts the

interfacial interactions (e.g., hydrogen bonding) between the PEGDA microcubes and the plasma-treated PDMS. In practical handling and distribution, however, PUF tags are rarely subjected to strong vibration while immersed in aqueous environments; vortexing therefore represents the vibration regime relevant to realistic use.

We further note that the detachment observed under bath sonication is consistent with prior reports on PEGDA particle harvesting from PDMS molds under solvent-assisted sonication conditions [ACS Appl. Polym. Mater. 2020, 2, 846–852].[3]

Based on this discussion, we have added the vortexing experimental result to Fig. S13 (mechanical stability), the bath sonication result to Fig. S14 (failure boundary), and revised Fig. 5d to consolidate HD values across all conditions, as follows:

(Figure 5d)

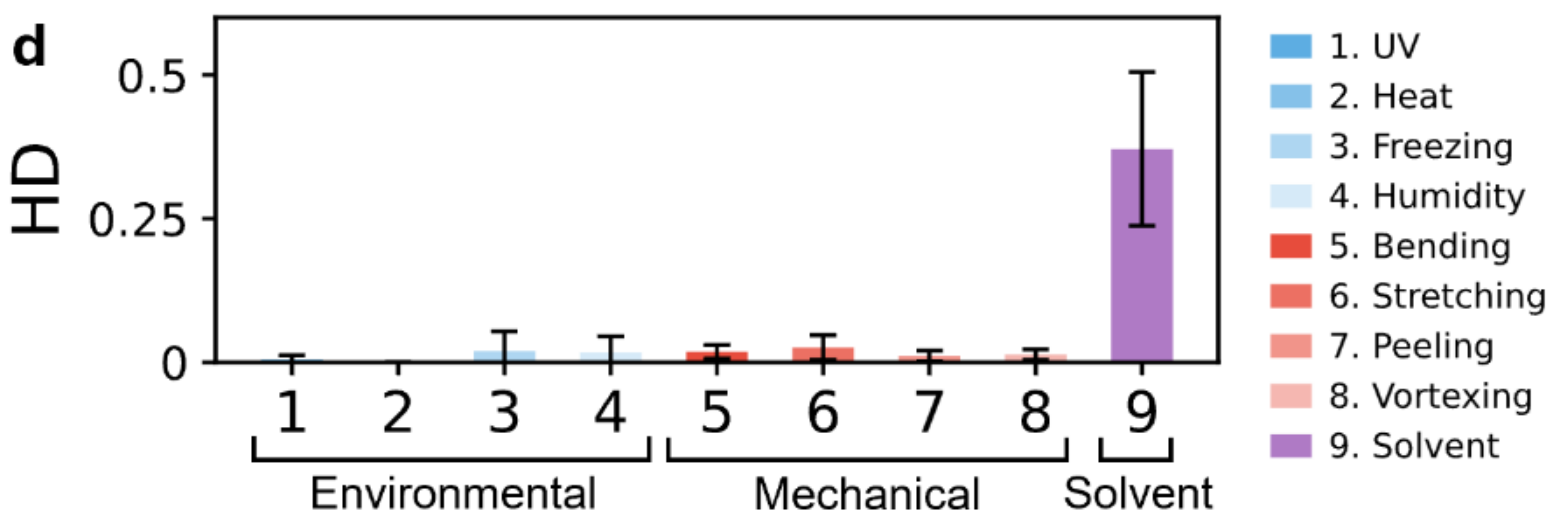

Figure 5d. HD values confirming structural stability under environmental stressors (1–4) and mechanical perturbations (5–8), contrasted with pronounced reconfiguration after solvent exposure (9).

(Figure S13)

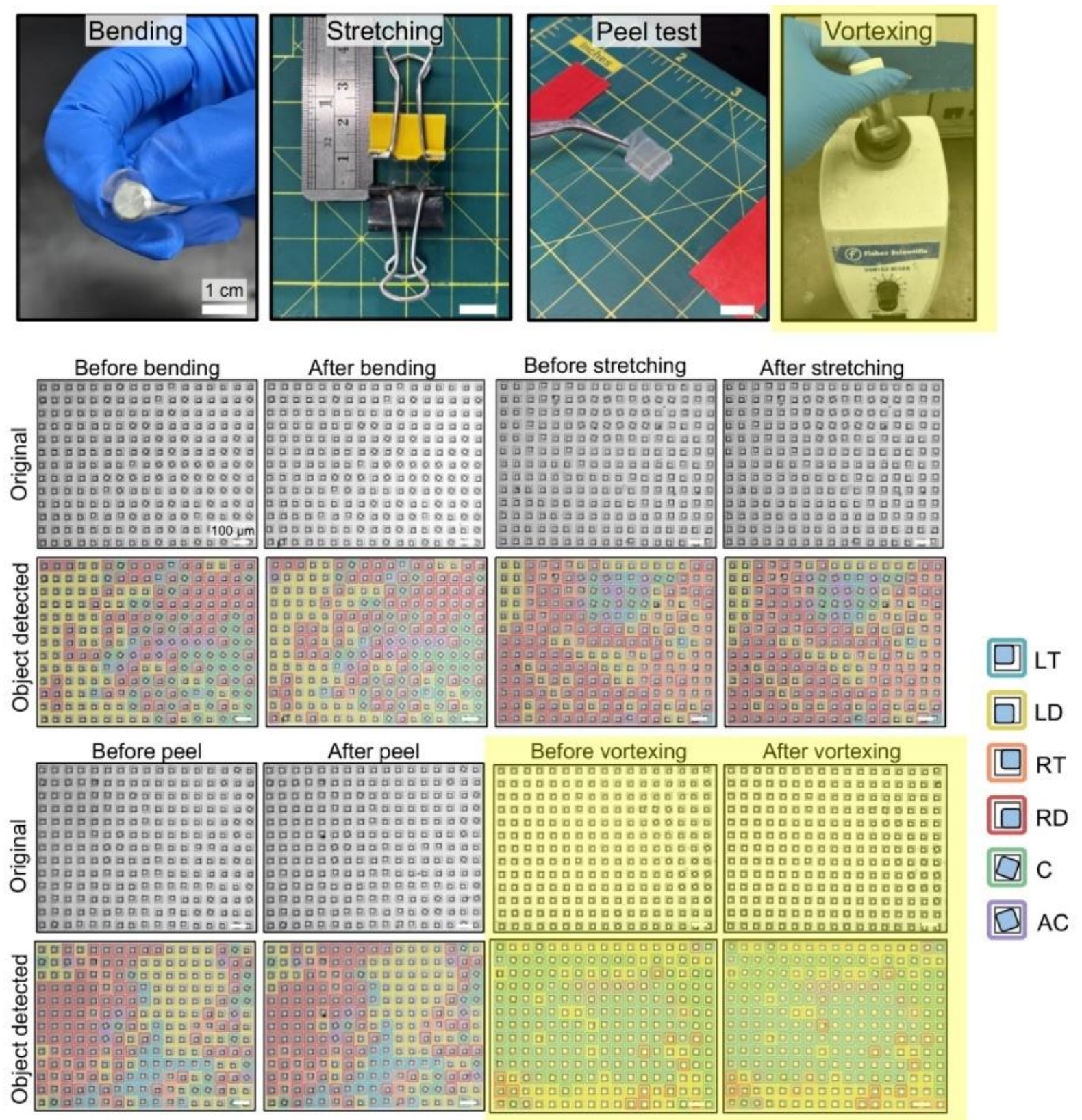

Figure S13. Mechanical stability test of a PUF under bending, stretching, peeling and vortex-induced vibration. Mechanical test setups and optical microscopy images of the encoded patterns before and after testing, with the corresponding Hamming Distance (HD) calculated using a fine-tuned object detection model (YOLOv10n).

(Figure S14)

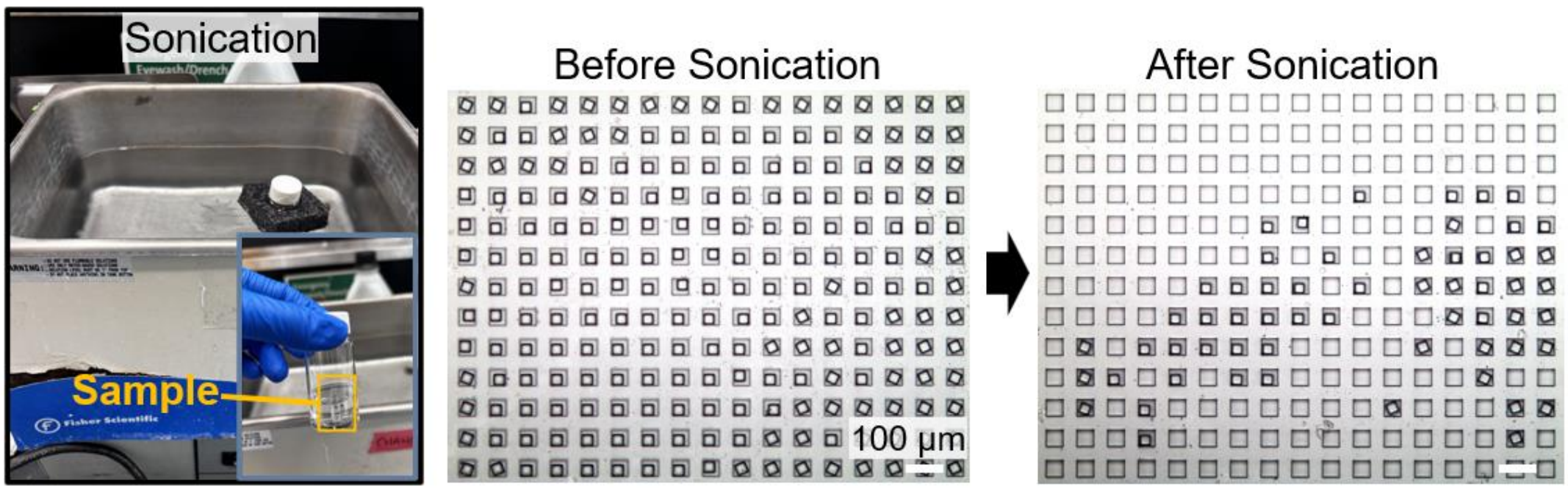

Figure S14. Detachment of microcubes from microwells under bath sonication in aqueous media.

(Page 12, line 250)

Furthermore, the tags demonstrate robust mechanical integrity, with encoded patterns remaining intact after repeated tape pull tests (100 cycles), cyclic bending (radius: 0.4 mm, 100 cycles), and cyclic stretching (20% strain, 50 cycles), and vortex-induced vibration (~2000 rpm, 30 min) (Supplementary Fig. 13); detachment occurs only under bath sonication in aqueous media, consistent with prior reports on microparticle harvesting from PDMS molds via ultrasonic agitation[3] (Supplementary Fig. 14).

2. The authors claim that the tag supports the full range of theoretical position. If this is the case, the process of generating each state should, in consequence, be of the same likeliness. This would mean, that the pattern formation is random, i.e., every new reset/set cycle, produces a new pattern. It would therefore be required to validate on a reasonable number of cycles, that no two pattern repeats. Given that the readout is automated to a high degree already using the YOLO model (which is a very solid choice), this should be reasonable to carry out. Every randomization-based identification requires pure randomness as input in order to prove that the system is not "physically skewed" - in a sense, has a tendency to produce only a subset of patterns.

[Author reply]

We thank the reviewer for raising this critical point. We agree that both state uniformity and cycle-to-cycle independence are essential for validating the stochastic nature of the reconfiguration process and for excluding potential physical bias. To address this concern, we performed two complementary experiments: (i) statistical evaluation of state uniformity using 80 independent 221-element PUF codes, and (ii) repeated solvent-triggering experiments across multiple independent samples (n = 5), each subjected to 5 reconfiguration cycles.

Before presenting these results, we first clarify a refinement to the state definition. The state space has been refined from a nominal 7-ary to a practically relevant 6-ary system. The 45°-rotated state is observed only in smaller microcubes in the lower-confinement regime ($\tilde{L} \approx 0.6$), where excessive free volume reduces swelling-induced wall contact and lowers reconfiguration reliability. In contrast, the operational regime in this study ($\tilde{L} \approx 0.75$) yields robust reconfiguration and an experimentally well-defined 6-state space (Fig. R4).

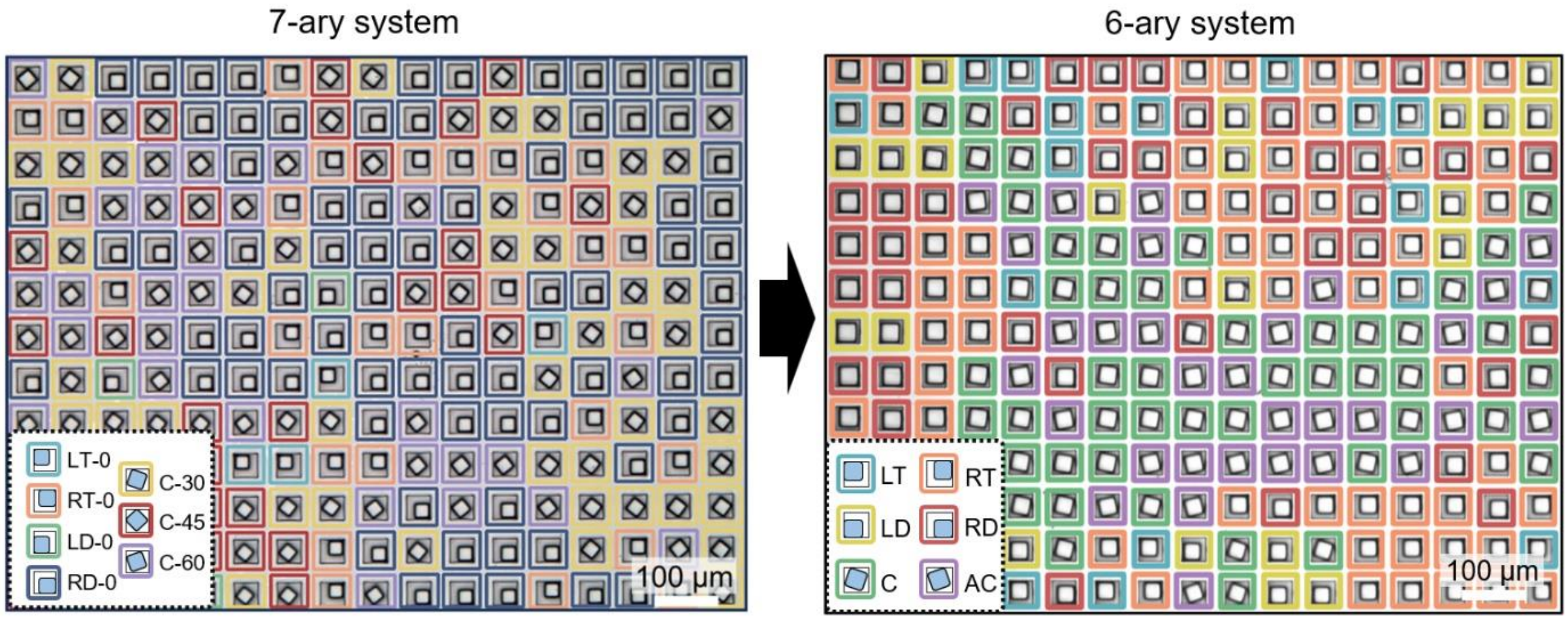

Figure R4. Refinement of the state definition from the previous 7-ary system to the revised 6-ary system.

(1) State uniformity and effective number of independent configurational states

State uniformity was evaluated using 80 PUF images based on the revised 6-ary classification (Fig. R5). The distribution is not perfectly uniform, with corner states being slightly more frequent than rotated states. However, the 95% confidence intervals for all six states include the ideal probability of $1/6(\approx 0.1667)$, indicating that the observed deviations from uniformity are modest and do not substantially bias the accessible state distribution.

To further quantify this effect, we computed the multistate Shannon entropy per site:[4]

$$H = -\sum_{s=1}^{6} p_s \log_2 p_s$$

where p_s is the probability of observing the state s across the dataset. The measured entropy is $H = 2.577$ bits, which is close to the ideal value $H_{\max} = \log_2 6 = 2.585$ bits.
The effective number of independent configurational states is defined by $N_{eff} = H/H_{ideal} \times 221 \approx 220$. We therefore estimate the effective encoding capacity as $6^{N_{eff}} = 6^{220}$. Thus, based on the revised 6-ary system, the encoding capacity approaches the ideal configurational space.

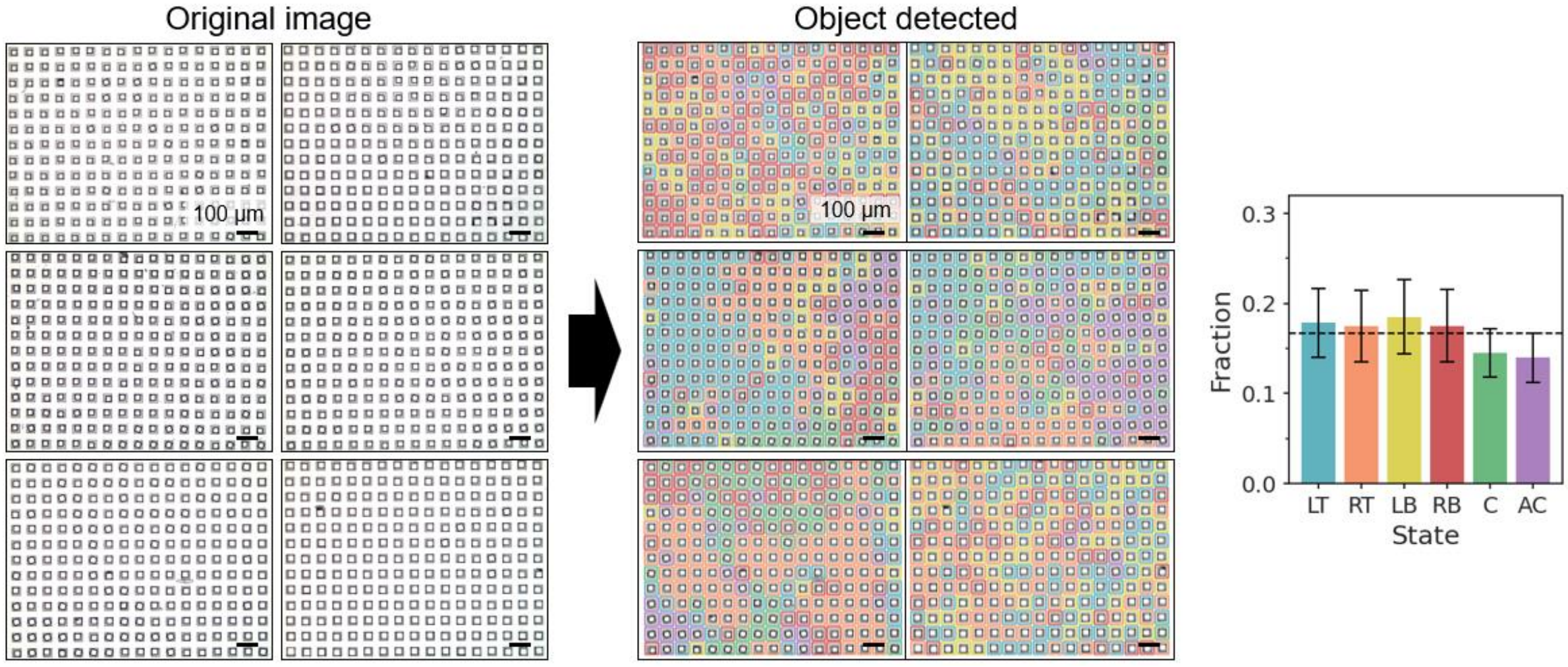

Figure R5. Representative examples from the 80-image dataset and corresponding state-uniformity analysis.

(2) Cycle-to-cycle independence
Cycle-to-cycle variability was first evaluated by measuring the Hamming distance between successive reconfiguration states (Fig. R6). The fractional Hamming distance remains approximately 0.37 over 5 cycles without a decreasing trend, indicating that successive states do not become progressively more

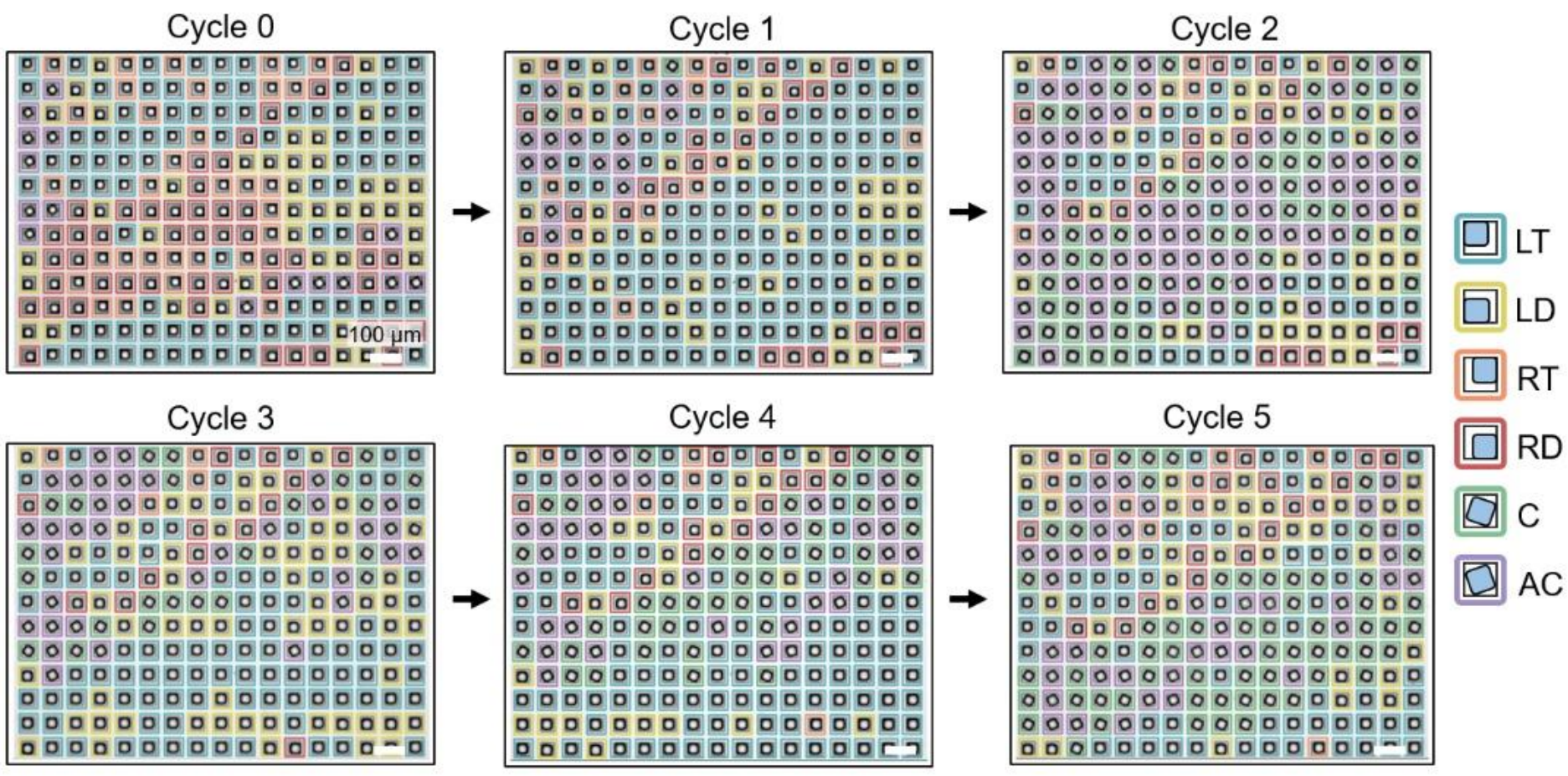

Figure R6. Sequential reconfiguration of the PUF pattern over 5 repeated cycles.

correlated. Although this value is lower than the ideal value of 5/6($\approx \mathbf{0.83}$) for a fully random 6-ary system, it remains sufficient to provide effective decorrelation between repeated reconfiguration cycles. To further assess statistical dependence between successive states, we quantified the normalized mutual information (MI) before and after each reconfiguration cycle. MI is defined as[4]

$$\mathrm{MI} = \frac{1}{log_2 6} \sum_{i=1}^{6} \sum_{j=1}^{6} p\,(i,j) log_2 \left(\frac{p(i,j)}{p_t(i)\, p_{t+1}(j)} \right)$$

where $p(i,j)$ is the joint probability that a site is in state i before reconfiguration and state j after reconfiguration, and $p_t(i)$ and $p_{t+1}(j)$ are the corresponding marginal probabilities at cycles t and $t+1$, respectively. By this normalization, MI = 0 indicates statistical independence, whereas MI = 1 indicates complete dependence.

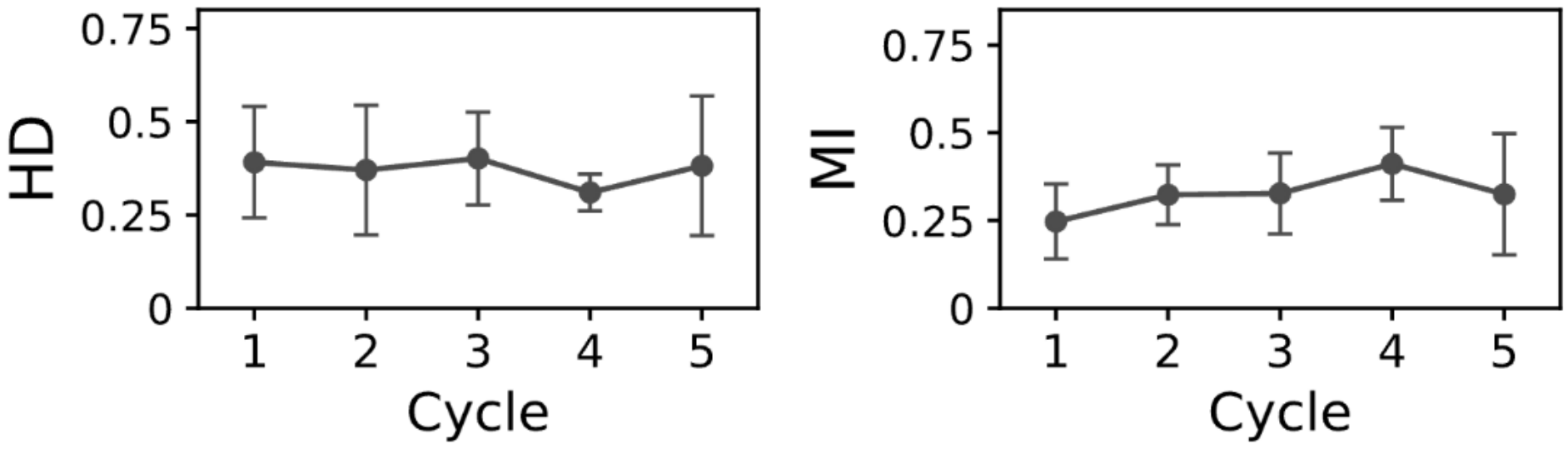

Figure R7. Cycle-to-cycle HD and MI over 5 repeated reconfiguration

The measured MI values range from 0.30 to 0.37 across 5 cycles (Fig. R7 and Supplementary Video 5), with an average value of 0.33. These results indicate partial dependence between consecutive reconfiguration cycles rather than ideal randomization. Using the measured state-distribution entropy of 2.577 bits per site, this average MI corresponds to a residual conditional uncertainty of $2.577 - 0.33 \times \log_2 6 \approx 1.72$ bits per site. Across 221 sites, this gives a residual configurational space of approximately 2^{381}, which is greater than the 256-bit security strength commonly referenced by the National Institute of Standards and Technology (NIST).[5] We therefore acknowledge this partial dependence while clarifying that the remaining configurational space remains sufficient under this reference scale.

We have added the state-uniformity analysis from Fig. R5 to Fig. 4e, included Fig. R6 as Supplementary Fig. S15 together with the corresponding Video S5, and added the MI analysis as Supplementary Fig. S16. We have revised the manuscript accordingly as follows:

(Page 11, line 218)

Based on the AI-assisted recognition results, we evaluate the PUF performance metrics. To assess randomness, we quantify the state uniformity of each state from 80 different PUF codes. Although corner states appear slightly more frequent than rotated states, the 95% confidence intervals for all six states include the ideal probability of 1/6 (≈0.1667), indicating comparable state probabilities (Fig. 4e). We further quantify the state-distribution uniformity using Shannon entropy. The calculated entropy is 2.577 bits, close to the ideal value of $\log_2 6$ (≈2.585 bits), corresponding to an effective encoding capacity of 6^{220} within a compact footprint of approximately 1.4 mm^2, ensuring sufficient density for secure authentication (see Supplementary Information for detailed definitions and calculations).

(Page 13, line 267)

To evaluate the repeatability and stochasticity of this process, we perform 5 consecutive solvent-triggered reconfiguration cycles on independent samples. The HD between consecutive cycles remains approximately 0.37, with no decreasing trend, confirming persistent cycle-to-cycle variation (Fig. 5e, Supplementary Fig. 15, and Supplementary Video 5). To further assess statistical dependence between consecutive reconfiguration cycles, we also calculate normalized mutual information (MI), which quantifies the dependence between site-state transitions before and after each cycle. The average MI is 0.33, where 0 denotes statistical independence and 1 denotes complete dependence (Supplementary Fig. 16). Although the cycle-to-cycle HD is lower than the ideal value of 5/6 for a fully random 6-ary system and the measured MI indicates partial dependence, no repeated patterns are observed over the tested cycles. The residual configurational space is estimated to be approximately 2^{381}, which exceeds the

256-bit security-strength level commonly referenced by the National Institute of Standards and Technology (NIST),[5] supporting practical resistance against exhaustive prediction-based attacks (see Methods in the Supplementary Information for details).

(Figure 4)

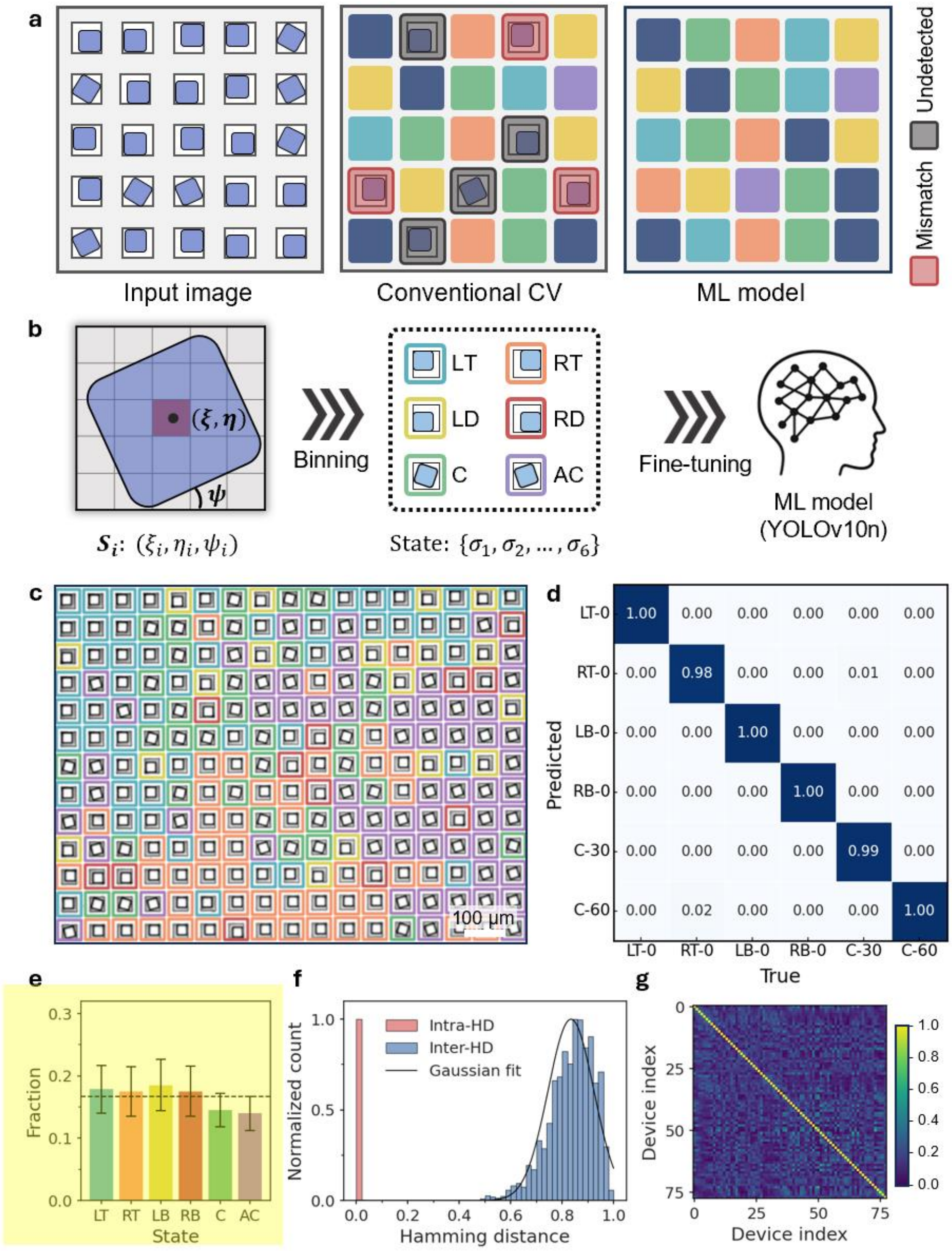

Figure 4. AI-assisted authentication framework and PUF performance metrics.
a, Comparative schematics of detection performance between conventional computer vision (CV)-based patch identification and the fine-tuned machine learning (ML) model. **b,** Discretization of the continuous microcube configuration space into six distinct states for fine-tuning the YOLOv10n object detection model. **c,** Representative experimental PUF image showing classification of 221 elements (13 × 17 array) into the six defined states. **d,** Confusion matrix demonstrating classification accuracies exceeding 98% across all states. **e,** State probability distribution. Error bars denote 95% confidence intervals. **f,** Distributions of Intra-HD and Inter-HD of the PUF responses. g, Cross-correlation map of PUF responses.

(Page S5, Methods section)

2) Effective encoding capacity

To quantify the deviation from ideal encoding capacity, we computed the multistate Shannon entropy per site:[4]

$$H = -\sum_{s=1}^{q} p_s \log_2 p_s$$

where p_s is the probability of observing the state s across the dataset and $q = 6$. The ideal entropy for equiprobable states is $H_{\max} = \log_2 6 = 2.585$bits. The total configurational entropy is $H_{\text{total}} = n \cdot H$, where $n = 221$ is the number of sites. The effective number of independent configurational states is then defined as

$$N_{\text{eff}} = \frac{H_{\text{total}}}{\log_2 6}$$

and the effective encoding capacity as $C_{\text{eff}} = 2^{H_{\text{total}}}$. Based on 80 PUF codes, we obtained $H = 2.577$ bits and $N_{\text{eff}} = 220$, yielding $C_{\text{eff}} = 6^{220}$.

3) Cycle-to-cycle independence

To evaluate statistical dependence between consecutive cycle outputs, we computed the normalized mutual information (MI) from the 6 × 6 transition matrix of site states between two successive cycles:

$$\text{MI} = \frac{1}{\log_2 6} \sum_{i=1}^{6} \sum_{j=1}^{6} p(i,j) \log_2 \left(\frac{p(i,j)}{p_t(i) p_{t+1}(j)} \right)$$

where $p(i,j)$ is the joint probability that a site is assigned to the state i at cycle t and state j at cycle $t+1$, and $p_t(i)$ and $p_{t+1}(j)$ are the corresponding marginal probabilities. For each cycle pair, $p(i,j)$ is obtained by counting the state transitions across 221 sites and normalizing by 221. By this definition, MI = 0 indicates statistical independence, whereas MI = 1 indicates complete dependence. The measured MI values range from 0.30 to 0.37 across 5 cycles, with an average value of 0.33. Using the measured state-distribution entropy of 2.577 bits per site, the residual conditional uncertainty is estimated as $2.577 - 0.33 \times \log_2 6 \approx 1.72$ bits per site. Across 221 sites, this gives a total residual uncertainty of $221 \times 1.75 \approx 381$ bits, corresponding to a residual configurational space of approximately 2^{381}, exceeding the 256-bit security-strength level commonly referenced in NIST cryptographic key-generation guidance.[5]

Reviewer #3

The authors present physical tags based on hydrogel microcubes embedded in polymer wells, with reconfigurability achieved via solvent immersion. The fabrication procedure is described in detail, including a careful selection of processing parameters. In addition, an AI-based authentication protocol is proposed to accelerate the identification process of the developed tags.
The manuscript is clear and well written. However, several fundamental aspects require deeper discussion and clarification.

[Author reply]
We sincerely appreciate the reviewer's thoughtful and detailed feedback, which has been very helpful in improving the clarity and rigor of our manuscript. We have carefully addressed all comments raised by the reviewer.

1. A primary concern relates to the potential clonability of the system after fabrication. The tag consists of an ordered array of wells containing microcubes. The initial state appears to exhibit limited intrinsic unclonability: following optical and chemical inspection (e.g., Raman spectroscopy, nanoindentation), an adversary could potentially reproduce the tag using similar materials, geometry, and fabrication procedures. The authors are therefore encouraged to clarify how clonability is prevented in practice and to discuss the robustness of the proposed system against reverse-engineering strategies.

[Author reply]
We thank the reviewer for this important point. With recent advances in nanofabrication—for example, two-photon polymerization (2PP) systems such as the Nanoscribe Quantum X platform and Microlight3D microFAB-3D, which achieve sub-200 nm resolution at scan speeds of hundreds of mm/s;[6] it is in principle conceivable to duplicate the geometry of a cube-in-well array in a non-monolithic fashion. However, reproducing our tag would require deterministic placement of hundreds of discrete elements, each with a specific translational and rotational configuration, inside ~45 μm PDMS microwells while preserving individual free volume across the full array, which remains practically challenging.
Moreover, material-level replication introduces several coupled physical constraints that further hinder practical cloning. Although Raman spectroscopy or nanoindentation could be used to infer chemical composition or bulk modulus for reverse engineering, the microcubes are formed via oxygen-inhibited photopolymerization, a reaction–diffusion-limited process that inherently generates spatial heterogeneity in crosslink density and interfacial properties. These features are not captured by bulk mechanical measurements and critically govern swelling behavior and detachment dynamics during reconfiguration. In addition, the plasma-induced silica-like crust layer on the PDMS microwells, which controls microcube dimensions by influencing oxygen diffusion, introduces further complexity, as its thickness and permeability are not readily accessible via conventional surface analysis. As a result, precisely reproducing the spatial gradients in material properties and surface characteristics, and consequently replicating the stochastic nature of the reconfiguration process, remains practically infeasible.
To clarify this point, we have revised the Discussion section as follows:

(Page 15, line 312)
To verify that the tag is not a clone replicating a registered code, the end user then applies a solvent droplet to test its reconfigurability. Successful reconfiguration occurs only for an authentic tag based on our r-PUF architecture, and the product is approved for use. In contrast, a counterfeit tag fabricated as a monolithic replica fails to undergo reconfiguration and is denied. Even non-monolithic replication would require reproducing the geometry and placement of individual microcubes, as well as the spatial crosslink-density gradients and cube-well interfacial properties. These features critically govern reconfiguration dynamics, yet are not readily accessible via conventional bulk and surface characterization.

2. The novelty and relevance of the work in the field of reconfigurable PUFs are not sufficiently articulated. As presented by the authors, the literature already includes several reconfigurable PUF implementations, including magnetic-optical and memristive systems that demonstrate strong environmental stability. Moreover, existing works describe both weak PUFs (limited challenge–response pairs) and strong PUFs (large CRP spaces). The authors are invited to more clearly highlight the specific advancements of their approach compared to the current state of the art. In addition, the claimed applications in "pharmaceuticals" (page 4, line 68) and "medical products" (Discussion, line 280) require further justification, as the practical advantage of solvent-based reconfiguration in these contexts is not evident. For completeness, the comparison table of optical reconfigurable PUFs could be expanded to include recent demonstrations such as the reconfigurable PDMS–LC composite reported in APL Photonics, 11(2), 2026.

[Author reply]

We sincerely appreciate the reviewer's constructive comment. We acknowledge that the advantages and advancements of our approach were not sufficiently articulated in the original manuscript.

Beyond environmental stability, **a central practical advantage of solvent-based reconfiguration is its ability to be deployed at the point of use without any specialized instrumentation.** Existing heat-, light-, or magnetic-based r-PUFs require dedicated instrumentation for reconfiguration (e.g., laser sources, heating stages, magnetic coils), which limits accessibility in real-world settings such as clinical environments. In contrast, our system uses a simple solvent trigger, which can be provided as a compact verification kit that accompanies the product. Since volatile solvents such as acetone are routinely handled in laboratory and clinical settings, no additional infrastructure is required. This distinction directly addresses the claimed pharmaceutical and medical applications: our two-step verification protocol can be performed at the point of care without any specialized apparatus.

To provide a more comprehensive comparison, we have expanded Table 1 to include recent magneto-optical systems [Magni et al., Adv. Funct. Mater. 35, e16376 (2025)] and recent reconfigurable optical PUFs [Salvestrini et al., APL Photonics 11, 020802 (2026); Kwak et al., Adv. Mater. 2026, e21712] for completeness.

Based on this discussion, we have revised the manuscript as follows:

(Page 4, line 58)

This vulnerability has motivated the development of reconfigurable optical PUFs (r-PUFs). Existing r-PUF approaches achieve reconfiguration through responsive media such as liquid crystals, inorganic phase-transition materials, or magneto-optical systems, allowing the encoded spatial configurations to be updated using light[7–10] heat,[11–17], or magnetic fields,[18] as summarized in Table 1. However, reconciling stability with practical reconfigurability remains challenging across these approaches. Light- and heat-based stimuli are inherently coupled to environmental exposure; some liquid crystal-based approaches exhibit low activation thresholds susceptible to unintended reconfiguration,[7,16] while inorganic phase-transition materials typically require harsh conditions incompatible with sensitive goods such as pharmaceuticals.[13–15] Magnetic-based approaches can avoid this environmental coupling but rely on a specialized setup for magnetic field generation, constraining field deployability in practical clinical environments.[18]

(Page 14, line 298)

To the best of our knowledge, this is the first demonstration of solvent-triggered reconfiguration in r-PUFs. Whereas conventional systems rely on external energy inputs such as light, heat, or magnetic fields, and require dedicated instrumentation for reconfiguration at the point of use, the solvent-triggered approach is orthogonal to ambient environmental factors and requires only a droplet of volatile solvent, which can be supplied as a compact verification kit accompanying the product. This makes it readily implementable in pharmaceutical and medical supply chains, preventing unintended code changes during transport while enabling on-demand reconfiguration accessible to end users without specialized equipment.

(Table 1)

Materials	Encoding capacity	States (per site)	Reconfiguration Stimuli	Stability	Remarks
Smectic LC[12]	2^{5700} [$432 \times 144\ \mu m^2$]	8	Thermal (212 °C)	Ambient 7 d; Thermal (100 °C 30 min) Humidity (70% RH)	Electrical-optical hybrid
$Ge_2Sb_2Te_5$ nanocrystal[13]	$2^{4147200}$ [$1\ cm^2$]	2	Thermal (255 °C)	–	–
VO_2 nanocrystal[14]	2^{655360}	2	Thermal (75 °C)	–	Reversible
Supersaturated sodium acetate solution[15]	2^{541} [$28 \times 28\ \mu m^2$]	2	Thermal (75 °C)	–	–
PDMS-LC composite[7]	2^{1750} [$1\ cm^2$]	≥ 10	Optical (60 mWcm^2, 470 nm)	–	Reversible
Cholesteric LC[16]	2^{5700} [$640 \times 640\ \mu m^2$]	4	Thermal (35 °C)	Thermal (100 °C)	–
Diamond Microparticles[17]	–	16	Thermal (600 °C)	Chemical (1 M NaOH, 1 M HCl, brine) Thermal (400 °C, 24 h) Light (365 nm & 532nm, 24 h) Humidity (75% RH 24 h) Mechanical abrasion	Air oxidation
Graphene-doped LCE[8]	–	2	Optical (30 mW, 980 nm)	Light (<10 mW, 532 nm)	–
Dye-doped PDLC[10]	–	2	Optical (450 nm)	–	–
$Fe_{70}Ga_{30}$ dot array[18]	2^{478} [$81 \times 60\ \mu m^2$]	3^8	Magnetic (25 mT)	Thermal (100 °C), UV (385 nm, 40 min)	–
Sintered AuNP array[9]	2^{346} [20 µm^2]	2	Optical (532 nm)	Thermal (150 °C, 1 h), Chemical (hexane, THF, IPA)	–
**This work**	6^{220} [$1.4\ mm^2$];	6	**Solvent-triggered (acetone)**	Light (15mW/cm^2, 365 nm, 24 h) Freezing (−20 °C, 24 h) Thermal (100 °C, 24 h) Humidity (75% RH, 24 h) Mechanical (bending, stretching, peeling, vortexing)	

Table 1. Summary of state-of-the-art reconfigurable optical physical unclonable functions.

3. How has the encoding capacity been calculated? The encoding capacity should be calculated based on the number of independent bits in the extracted key, and not taking into account the total bit length (number of cubes per image) of the response string. The authors should therefore estimate the effective number of independent configurational states.

[Author reply]

We sincerely appreciate the reviewer's helpful comment. We agree that the encoding capacity should be estimated based on the effective number of independent configurational states. Before presenting these results, we first introduce a refinement to the state definition. The state space has been refined from a nominal 7-ary to a practically relevant 6-ary system. The 45°-rotated state is observed only in smaller microcubes in the lower-confinement regime ($\tilde{L} \approx 0.6$), where excessive free volume reduces swelling-induced wall contact and lowers reconfiguration reliability. In contrast, the operational regime in this study ($\tilde{L} \approx 0.75$) yields robust reconfiguration and an experimentally well-defined 6-state space (Fig. R8).

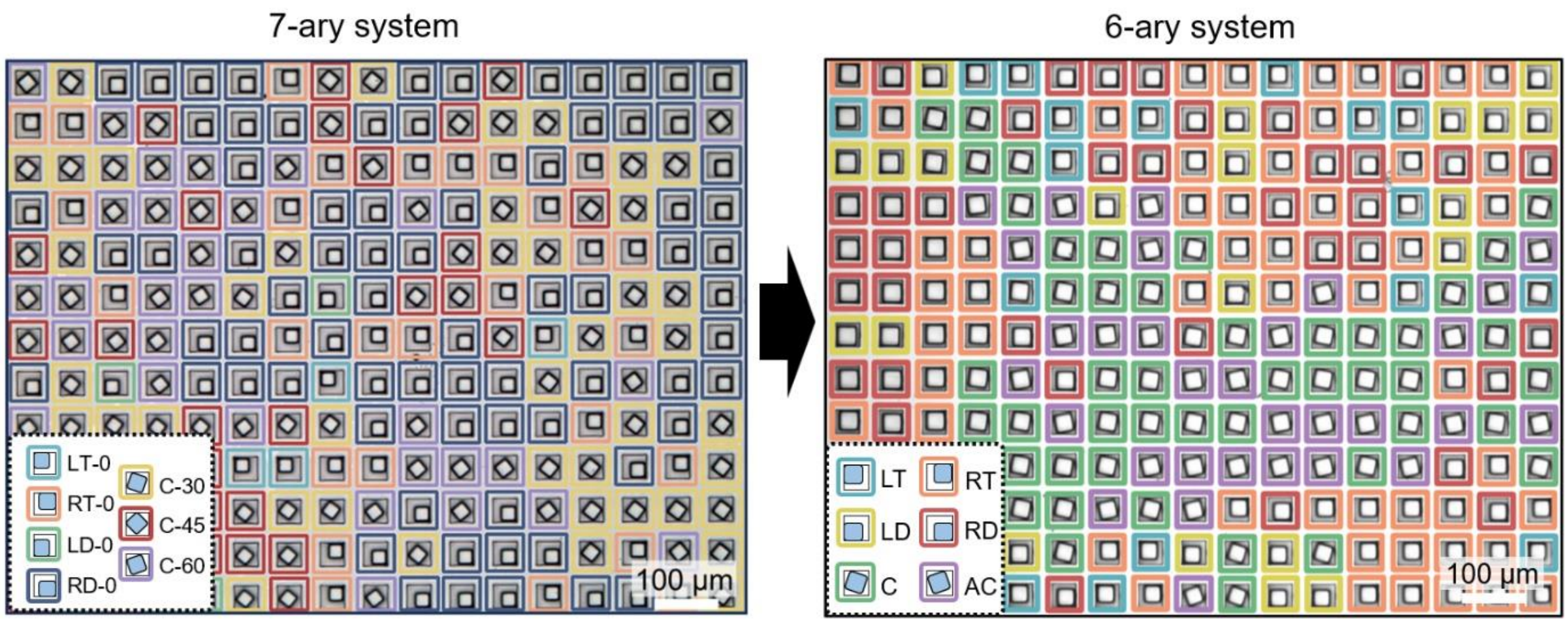

Figure R8. Refinement of the state definition from the previous 7-ary system to the revised 6-ary system.

To calculate effective encoding capacity, we evaluated state uniformity using 80 different PUF images based on the revised 6-ary classification (Fig. R9). The distribution is not perfectly uniform, with corner states being slightly more frequent than rotated states. However, the 95% confidence intervals for all six states include the ideal probability of 1/6 (≈0.1667), indicating that the observed deviations from uniformity are modest and do not substantially bias the accessible state distribution.
To further quantify this effect, we computed the multistate Shannon entropy per site:[4]

$$H = -\sum_{s=1}^{6} p_s \log_2 p_s$$

where p_s is the probability of observing the state s across the dataset. The measured entropy is $H = 2.577$ bits, which is close to the ideal value $H_{\max} = \log_2 6 = 2.585$ bits.
The effective number of independent configurational states is defined by $N_{eff} = H/H_{ideal} \times 221 \approx 220$. We therefore estimate the effective encoding capacity as $6^{N_{eff}} = 6^{220}$. Thus, based on the revised 6-ary system, the encoding capacity approaches the ideal configurational space.

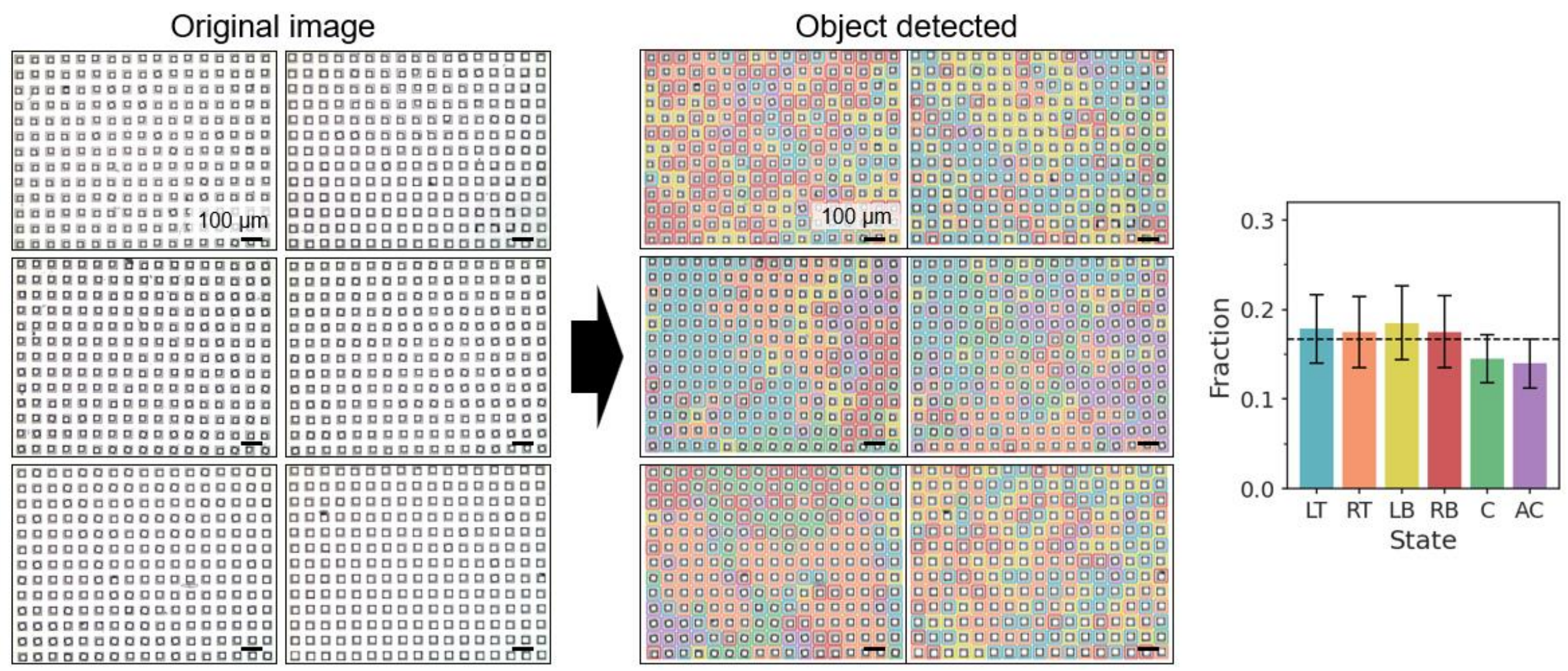

Figure R9. Representative examples from the 80-image dataset and corresponding state-uniformity analysis.

Based on this analysis, we now report the effective encoding capacity of our system as 6^{220} and have updated Table 1 accordingly; the manuscript has been revised as follows:

(Page 11, line 218)

Based on the AI-assisted recognition results, we evaluate the PUF performance metrics. To assess randomness, we quantify the state uniformity of each state from 80 different PUF codes. Although corner states appear slightly more frequent than rotated states, the 95% confidence intervals for all six states include the ideal probability of 1/6 (≈0.1667), indicating comparable state probabilities (Fig. 4e). We further quantify the state-distribution uniformity using Shannon entropy. The calculated entropy is 2.577 bits, close to the ideal value of $\log_2 6$ (≈2.585 bits), corresponding to an effective encoding capacity of 6^{220} within a compact footprint of approximately 1.4 mm^2, ensuring sufficient density for secure authentication (see Supplementary Information for detailed definitions and calculations).

(Page S5, Methods section)

2) Effective encoding capacity

To quantify the deviation from ideal encoding capacity, we computed the multistate Shannon entropy per site:[4]

$$H = -\sum_{s=1}^{q} p_s \log_2 p_s$$

where p_s is the probability of observing the state s across the dataset and $q = 6$. The ideal entropy for equiprobable states is $H_{\max} = \log_2 6 = 2.585$bits. The total configurational entropy is $H_{\text{total}} = n \cdot H$, where $n = 221$ is the number of sites. The effective number of independent configurational states is then defined as

$$N_{\text{eff}} = \frac{H_{\text{total}}}{\log_2 6}$$

and the effective encoding capacity as $C_{\text{eff}} = 2^{H_{\text{total}}}$. Based on 80 PUF codes, we obtained $H = 2.577$ bits and $N_{\text{eff}} = 220$, yielding $C_{\text{eff}} = 6^{220}$.

4. To demonstrate the security of the system, all possible microcube arrangements should ideally be equiprobable. However, from Figure 3c–e, corner states appear more likely than others, suggesting possible bias in the configurational distribution. The authors should provide a statistical analysis of the state uniformity. Furthermore, for the reconfiguration process to be unpredictable, successive states should be statistically independent of previous configurations. The authors should therefore quantify the correlation coefficient between states before and after reconfiguration (e.g., Figures S12–S16) and analyze how this correlation evolves over multiple reconfiguration cycles. If state equiprobability is not verified, the implications for security—particularly under repeated reconfiguration—should be discussed, including vulnerability to modeling or prediction attacks.

[Author reply]

We appreciate the reviewer for raising this important point. As described in our response to Comment 3, we analyzed state uniformity based on 80 different PUF tags. To further evaluate whether repeated reconfiguration produces independent patterns, we performed 5 consecutive solvent-triggered reconfiguration cycles on independent samples (n = 5). A representative reconfiguration video is provided as Supplementary Video 5, and the corresponding still images are shown in Fig. R10.

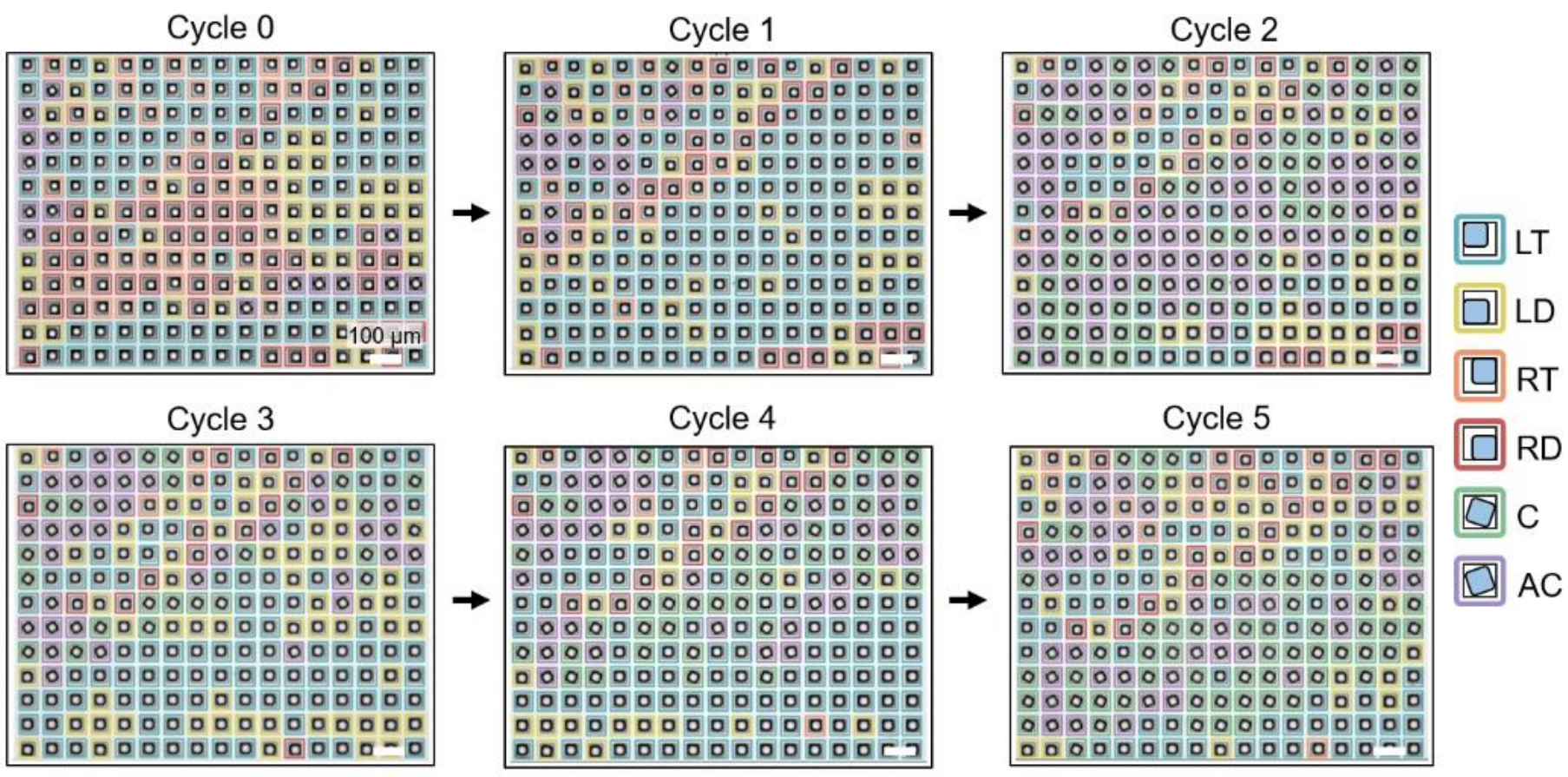

Figure R10. Sequential reconfiguration of the PUF pattern over 5 repeated cycles.

Cycle-to-cycle variability was first evaluated by measuring the Hamming distance between successive reconfiguration states (Fig. R11). The fractional Hamming distance remains approximately 0.37 over 5 cycles, with no decreasing trend, indicating persistent cycle-to-cycle variation. Although this value is lower than the ideal value of 5/6 (≈0.83) for a fully random 6-ary system, no repeated patterns are observed over the tested cycles.

To further assess statistical dependence between successive states, we quantified the normalized mutual information (MI) before and after each reconfiguration cycle. MI is defined as[4]

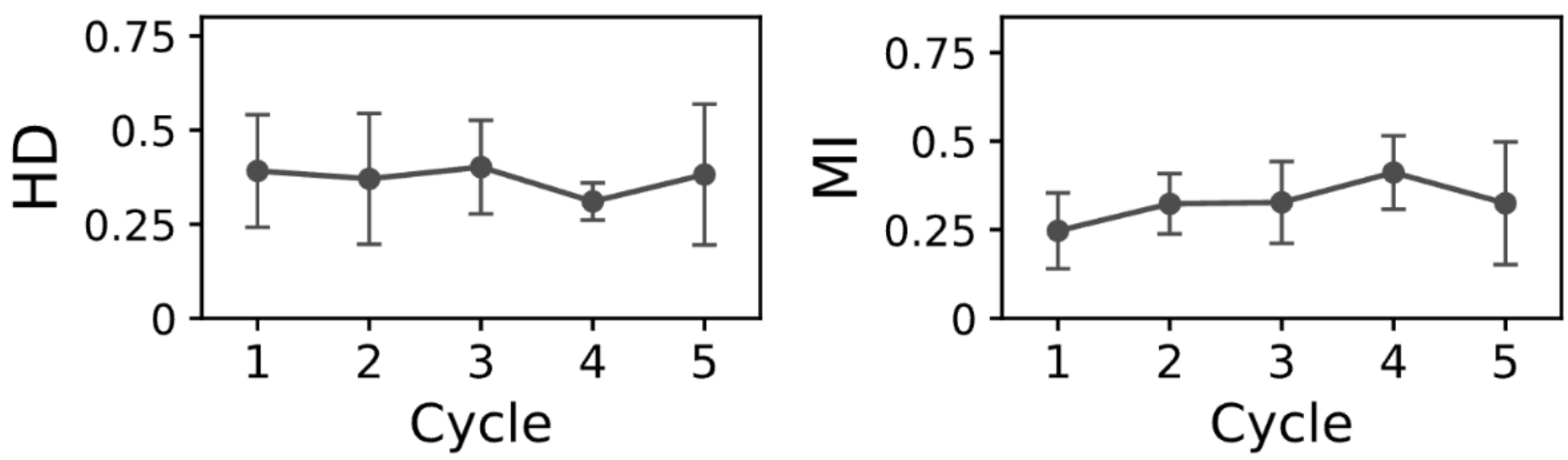

Figure R11. Cycle-to-cycle HD and MI over 5 repeated reconfiguration cycles.

$$\text{MI} = \frac{1}{log_2 6}\sum_{i=1}^{6}\sum_{j=1}^{6} p\,(i,j) log_2\left(\frac{p(i,j)}{p_t(i)\, p_{t+1}(j)}\right)$$

where $p(i,j)$ is the joint probability that a site is in state i before reconfiguration and state j after reconfiguration, and $p_t(i)$ and $p_{t+1}(j)$ are the corresponding marginal probabilities at cycles t and $t+1$, respectively. By this normalization, MI = 0 indicates statistical independence, whereas MI = 1 indicates complete dependence.

The measured MI values range from 0.30 to 0.37 across 5 cycles (Fig. R11 and Supplementary Video 5), with an average value of 0.33. These results indicate partial dependence between consecutive reconfiguration cycles rather than ideal randomization. Using the measured state-distribution entropy of 2.577 bits per site, this average MI corresponds to a residual conditional uncertainty of approximately $2.577 - 0.33 \times \log_2 6 \approx 1.72$ bits per site. Across 221 sites, this gives a residual configurational space of approximately 2^{381}, which is exceeds the 256-bit security strength commonly referenced by the National Institute of Standards and Technology (NIST).[5] We therefore acknowledge this partial dependence while clarifying that the remaining configurational space remains sufficient under this reference scale.

Based on this analysis, we have added the state-uniformity analysis from Fig. R9 to Fig. 4e, included Fig. R10 as Supplementary Fig. S15 together with the corresponding Video S5, and added the MI analysis as Supplementary Fig. S16. We have revised the manuscript accordingly as follows:

(Page 11, line 218)

Based on the AI-assisted recognition results, we evaluate the PUF performance metrics. To assess randomness, we quantify the state uniformity of each state from 80 different PUF codes. Although corner states appear slightly more frequent than rotated states, the 95% confidence intervals for all six states include the ideal probability of 1/6 (≈0.1667), indicating comparable state probabilities (Fig. 4e). We further quantify the state-distribution uniformity using Shannon entropy. The calculated entropy is 2.577 bits, close to the ideal value of $\log_2 6$ (≈2.585 bits), corresponding to an effective encoding capacity of 6^{220} within a compact footprint of approximately 1.4 mm^2, ensuring sufficient density for secure authentication (see Supplementary Information for detailed definitions and calculations).

(Page 13, line 269)

To evaluate the repeatability and stochasticity of this process, we perform 5 consecutive solvent-triggered reconfiguration cycles on independent samples. The HD between consecutive cycles remains approximately 0.37, with no decreasing trend, confirming persistent cycle-to-cycle variation (Fig. 5e, Supplementary Fig. 15, and Supplementary Video 5). To further assess statistical dependence between consecutive reconfiguration cycles, we also calculate normalized mutual information (MI), which quantifies the dependence between site-state transitions before and after each cycle. The average MI is 0.33, where 0 denotes statistical independence and 1 denotes complete dependence (Supplementary Fig. 16). Although the cycle-to-cycle HD is lower than the ideal value of 5/6 for a fully random 6-ary system and the measured MI indicates partial dependence, no repeated patterns are observed over the tested cycles. The residual configurational space is estimated to be approximately 2^{381}, which exceeds the 256-bit security-strength level commonly referenced by the National Institute of Standards and Technology (NIST),[5] supporting practical resistance against exhaustive prediction-based attacks (see Methods in the Supplementary Information for details).

(Figure 4)

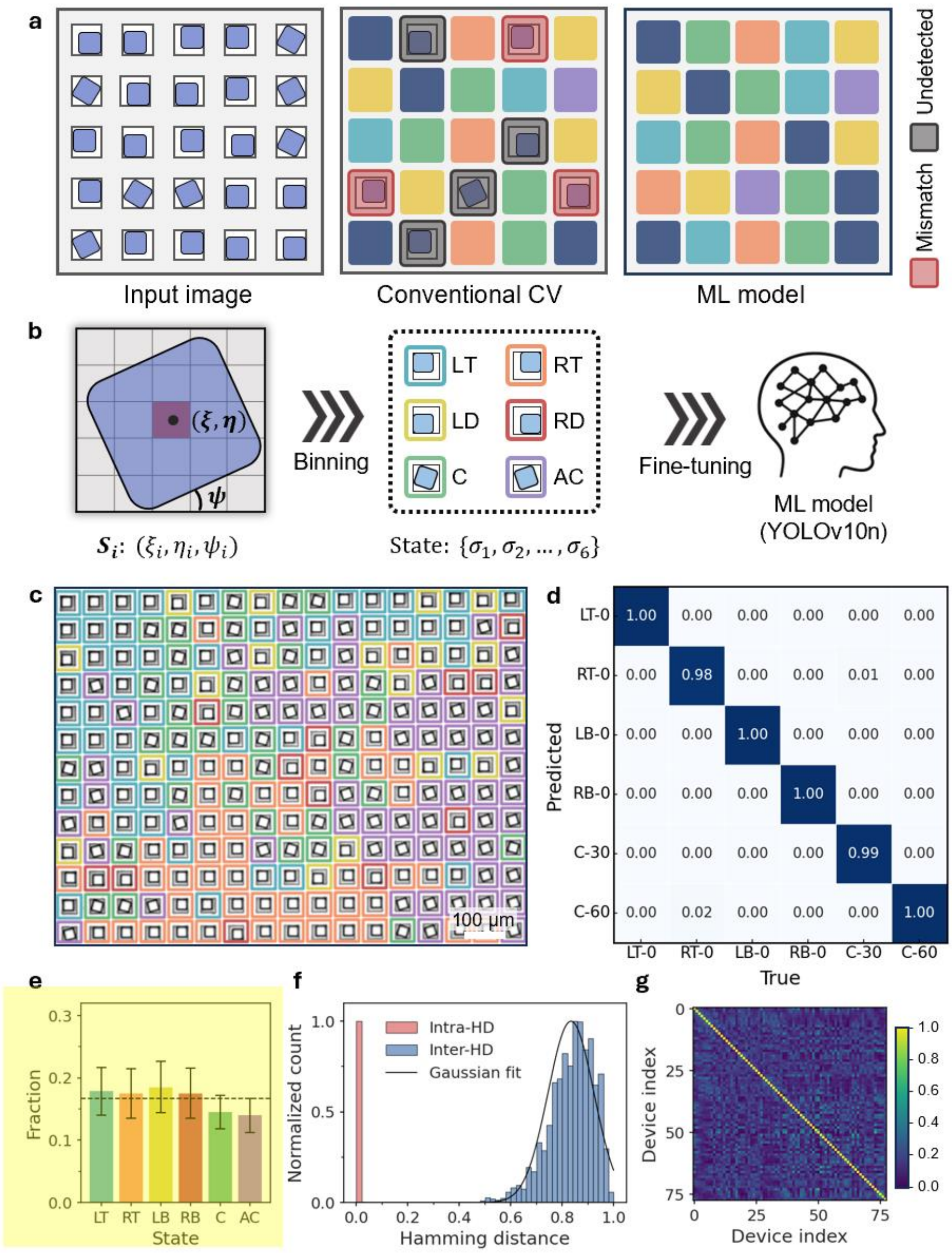

Figure 4. AI-assisted authentication framework and PUF performance metrics.
a, Comparative schematics of detection performance between conventional computer vision (CV)-based patch identification and the fine-tuned machine learning (ML) model. **b,** Discretization of the continuous microcube configuration space into six distinct states for fine-tuning the YOLOv10n object detection model. **c,** Representative experimental PUF image showing classification of 221 elements (13 × 17 array) into the six defined states. **d,** Confusion matrix demonstrating classification accuracies exceeding 98% across all states. **e,** State probability distribution. Error bars denote 95% confidence intervals. **f,** Distributions of Intra-HD and Inter-HD of the PUF responses. g, Cross-correlation map of PUF responses.

(Page S5, Methods Section)

3) Cycle-to-cycle independence

To evaluate statistical dependence between consecutive cycle outputs, we computed the normalized mutual information (MI) from the 6 × 6 transition matrix of site states between two successive cycles:

$$\mathrm{MI} = \frac{1}{\log_2 6}\sum_{i=1}^{6}\sum_{j=1}^{6} p(i,j)\log_2\left(\frac{p(i,j)}{p_t(i)p_{t+1}(j)}\right)$$

where $p(i,j)$ is the joint probability that a site is assigned to state i at cycle t and state j at cycle $t+1$, and $p_t(i)$ and $p_{t+1}(j)$ are the corresponding marginal probabilities. For each cycle pair, $p(i,j)$ is obtained by counting the state transitions across 221 sites and normalizing by 221. By this definition, MI = 0 indicates statistical independence, whereas MI = 1 indicates complete dependence. The measured MI values range from 0.30 to 0.37 across 5 cycles, with an average value of 0.33. Using the measured state-distribution entropy of 2.577 bits per site, the residual conditional uncertainty is estimated as $2.577 - 0.33 \times \log_2 6 \approx 1.72$ bits per site. Across 221 sites, this gives a total residual uncertainty of $221 \times 1.72 \approx 381$ bits, corresponding to a residual configurational space of approximately 2^{381}, exceeding the 256-bit security-strength level commonly referenced in NIST cryptographic key-generation guidance.[5]

5. The definition of generalized fractional Hamming distance (FHD) for a base of seven states requires a more rigorous and formal derivation. The generalized uniqueness metric presented appears to evaluate the randomness across cube states rather than the independence of the tag before and after reconfiguration. This distinction should be clarified. Additionally, the expected theoretical uniqueness value for a 7-state-base PUF should be explicitly derived and compared with experimental results.

[Author reply]

We agree that this distinction was not sufficiently clear in the original manuscript. The generalized Inter-HD $\left(\frac{2}{N(N-1)}\sum_{i=1}^{N-1}\sum_{j=i+1}^{N}\frac{HD(R_i,R_j)}{n}\right)$, as defined in the Supplementary Information, quantifies uniqueness between 80 different PUF responses, i.e., between distinct tags, and does not describe the statistical independence of a tag before and after reconfiguration. We address cycle-to-cycle independence separately in our response to Comment 4.
We have revised the manuscript to clarify this point explicitly:

(Page 11, line 227)
To assess reliability, we quantify the similarity between repeated readouts of a single PUF code using the Intra-Hamming distance (Intra-HD), defined as the fraction of mismatched elements between two PUF codes (see Supplementary Information for detailed definitions). The Intra-HD, calculated from 50 repeated responses of a single PUF code, yields a low mean of 0.0036 ± 0.0049, indicating high response stability and reliability (Fig. 4f). To evaluate uniqueness, we analyze the Inter-Hamming distance (Inter-HD) across 80 different PUF codes. The generalized Inter-HD is 0.8341 ± 0.0892, which approximates the theoretical value of $5/6$(≈ 0.8333) for a 6-ary system (Fig. 4f). We note that Inter-HD quantifies uniqueness across different PUF tags; code variation upon repeated reconfiguration is discussed in the following section.

(Page S4, Methods Section)
For a q-ary system with equiprobable states, the probability that two independently generated sites are in different states is $(q-1)/q$. Since the fractional Hamming distance is the fraction of differing sites, the expected Inter-HD is $(q-1)/q$, yielding a theoretical value of $5/6 \approx 0.833$ for $q = 6$.

6. Regarding Figure 4f, clarification is needed on how correlation coefficients were calculated. Based on the optical images presented in both the main text and the supplementary material, the correlation coefficients computed over the entire image are expected to be close to 1. The authors should therefore specify whether image processing, or feature extraction steps were applied, and whether correlation was calculated globally or on segmented regions.

[Author reply]
We agree that our original description of the correlation analysis was not sufficiently clear. In Fig. 4f, the correlation coefficients were not calculated directly from the full optical images. Instead, each optical image was first converted into a 221-element encoded response vector after individual cube–well units were identified and classified using the fine-tuned YOLO model. The correlation matrix was then calculated between these extracted PUF response vectors. We have revised the manuscript to clarify this analysis procedure explicitly.
To clarify this point, we have revised the relevant sentence as follows:

(Page 11, line 235)
We note that Inter-HD quantifies uniqueness across different PUF tags; code variation upon repeated reconfiguration is discussed in the following section. Furthermore, the pairwise correlation matrix of the encoded 221-element PUF responses exhibits a prominent diagonal, indicating that high correlations occur only for identical patterns (Fig. 4g).

References

1. Cuccia, N.L., Pothineni, S., Wu, B., Méndez Harper, J., and Burton, J.C. (2020). Pore-size dependence and slow relaxation of hydrogel friction on smooth surfaces. Proc. Natl. Acad. Sci. *117*, 11247–11256. https://doi.org/10.1073/pnas.1922364117.

2. Drira, Z., and Yadavalli, V.K. (2013). Nanomechanical measurements of polyethylene glycol hydrogels using atomic force microscopy. J. Mech. Behav. Biomed. Mater. *18*, 20–28. https://doi.org/10.1016/j.jmbbm.2012.09.015.

3. Oberdick, S.D., and Zabow, G. (2020). Patterned Surface Energy in Elastomeric Molds as a Generalized Approach to Polymer Particle Fabrication. ACS Appl. Polym. Mater. *2*, 846–852. https://doi.org/10.1021/acsapm.9b01109.

4. Shannon, C.E. (1948). A mathematical theory of communication. Bell Syst. Tech. J. *27*, 379–423. https://doi.org/10.1002/j.1538-7305.1948.tb01338.x.

5. Barker, E., Roginsky, A., and Davis, R. (2020). Recommendation for Cryptographic Key Generation (National Institute of Standards and Technology) https://doi.org/10.6028/NIST.SP.800-133r2.

6. Boudene, I., and Bougdid, Y. (2025). Two-photon polymerization-assisted 3D laser nanoprinting: from fundamentals to modern applications. J. Mater. Chem. C *13*, 18597–18630. https://doi.org/10.1039/D5TC02037A.

7. Nocentini, S., Rührmair, U., Barni, M., Wiersma, D.S., and Riboli, F. (2024). All-optical multilevel physical unclonable functions. Nat. Mater. *23*, 369–376. https://doi.org/10.1038/s41563-023-01734-7.

8. He, Z., Cao, B., Zhang, Y., Zhu, H., Wang, X., Li, C., and Zhang, W. (2025). Reversible and Reconfigurable Optical Physical Unclonable Functions with Elastic Liquid Crystal Scatterers. Nano Lett. *25*, 15542–15549. https://doi.org/10.1021/acs.nanolett.5c03711.

9. Kwak, J.-K., Moon, C., Yu, H.-B., Kim, S., and Kim, D.-H. All-Optical Reconfigurable Physical Unclonable Function for Sustainable Security. Adv. Mater. *n/a*, e21712. https://doi.org/10.1002/adma.202521712.

10. Salvestrini, S., Maestri, F., Lio, G.E., Krajinic, F., Savic-Sevic, S., Muric, B., Pantelic, D., Jelenkovic, B., Wiersma, D.S., Nocentini, S., et al. (2026). Speckle analysis of reconfigurable disordered photonic materials made of polymer dispersed liquid crystals. APL Photonics *11*, 020802. https://doi.org/10.1063/5.0282151.

11. Dodda, A., Subbulakshmi Radhakrishnan, S., Schranghamer, T.F., Buzzell, D., Sengupta, P., and Das, S. (2021). Graphene-based physically unclonable functions that are reconfigurable and resilient to machine learning attacks. Nat. Electron. *4*, 364–374. https://doi.org/10.1038/s41928-021-00569-x.

12. Yun, H.S., Wei, D., Yang, S., Park, G., Kim, M.S., Shin, T.J., Walba, D.M., Han, M.J., and Yoon, D.K. (2025). Reconfigurable Liquid Crystal-Based Physical Unclonable Function Integrating Optical and Electrical Responses. Adv. Mater. *37*, 2504288. https://doi.org/10.1002/adma.202504288.

13. Yang, Y., Hui, R., Lu, X., Tang, L., Gan, Z., Li, M., Zhang, J., Chen, K., Chen, F., and Huang, W. (2024). Optical True Random Number Generator Based on Reconfigurable Ge2Sb2Te5 PUFs. ACS Photonics *11*, 4691–4699. https://doi.org/10.1021/acsphotonics.4c01148.

14. Gan, Z., Chen, F., Li, Q., Li, M., Zhang, J., Lu, X., Tang, L., Wang, Z., Shi, Q., Zhang, W., et al. (2022). Reconfigurable Optical Physical Unclonable Functions Enabled by VO2 Nanocrystal Films. ACS Appl. Mater. Interfaces *14*, 5785–5796. https://doi.org/10.1021/acsami.1c20803.

15. Kim, Y., Lim, J., Lim, J.H., Hwang, E., Lee, H., Kim, M., Ha, I., Cho, H., Kwon, J., Oh, J., et al. (2023). Reconfigurable Multilevel Optical PUF by Spatiotemporally Programmed Crystallization of Supersaturated Solution. Adv. Mater. *35*, 2212294. https://doi.org/10.1002/adma.202212294.

16. Park, G., Choi, Y.-S., Kwon, S.J., and Yoon, D.K. (2023). Planar Spin Glass with Topologically Protected Mazes in the Liquid Crystal Targeting for Reconfigurable Micro Security Media. Adv. Mater. *35*, 2303077. https://doi.org/10.1002/adma.202303077.

17. Zhang, T., Wang, L., Wang, J., Wang, Z., Gupta, M., Guo, X., Zhu, Y., Yiu, Y.C., Hui, T.K.C., Zhou, Y., et al. (2023). Multimodal dynamic and unclonable anti-counterfeiting using robust diamond microparticles on heterogeneous substrate. Nat. Commun. *14*, 2507. https://doi.org/10.1038/s41467-023-38178-1.

18. Magni, A., Barrera, G., Celegato, F., Riboli, F., Wiersma, D.S., Nocentini, S., and Tiberto, P. (2025). Magnetic Unclonable Functions Leveraging Remanence and Anhysteretic States. Adv. Funct. Mater. *35*, e16376. https://doi.org/10.1002/adfm.202516376.

Notes on revision made to manuscript NCOMMS-26-015112A:

Solvent-Triggered Reconfiguration of Optical Physical Unclonable Functions

Ji Hoon Kim, [1,2,3] Jihee Kim, [2] Jung Gun Bae, [1] Junsik Choi, [2] Seongsu Cho, [3] Daeyeon Lee, [*3] Hyunsik Yoon, [*2] and Won Bo Lee [*1]

[1]Department of Chemical and Biological Engineering, Institute of Chemical Processes, Seoul National University, 08826, Republic of Korea
[2]Department of Chemical and Biomolecular Engineering, Seoul National University of Science and Technology, Seoul, 01811, Republic of Korea
[3]Department of Chemical and Biomolecular Engineering, University of Pennsylvania, Philadelphia, Pennsylvania, 19104, USA

*Corresponding authors

Daeyeon Lee: daeyeon@seas.upenn.edu
Hyunsik Yoon: hsyoon@seoultech.ac.kr
Won Bo Lee: wblee@snu.ac.kr

Point-by-point response to reviewers' comments

Reviewer #1

The authors have addressed all my comments. I recommend publication.

[Author reply]
We appreciate the reviewer's positive evaluation of our revised manuscript and their effort throughout the review process.

Reviewer #2

The authors have done an excellent job revising the paper which is now ready for publication.

[Author reply]
We are grateful for the reviewer's encouraging assessment of our revised manuscript and helpful comments during the review process.

Reviewer #3

The authors have addressed the reviewers' concerns by providing additional experiments and clarifications, significantly improving the manuscript. However, the proposed PUF does not demonstrate fully solvent reconfigurability.
In particular, although the authors revised the PUF state definition from a 7-ary to a 6-ary system and showed improvements in key PUF metrics such as bit uniformity and unclonability, the achieved reconfiguration performance remains not optimal because of the presence of residual correlations among the different responses. The stochasticity of the reconfiguration process reported in the article is not supported by the data analysis. The authors are invited to better discuss or revise this point.

[Author reply]
We appreciate the reviewer's careful and constructive comment. We agree that the reconfiguration should not be interpreted as a fully stochastic or statistically independent randomization process. Accordingly, we have removed the word "stochastic" from the title and revised the relevant descriptions throughout the manuscript to avoid overstatement. In addition, based on our mutual information (MI) analysis, we now explicitly acknowledge the residual statistical dependence between consecutive responses, while clarifying its implication for practical security.

(Page 13, line 278)

The cycle-to-cycle HD is lower than the ideal value of 5/6 for a fully random 6-ary system, and the measured MI indicates residual statistical dependence between consecutive responses. Nevertheless, the residual configurational space is estimated to be approximately 2^{381}, which exceeds the 256-bit security-strength level commonly referenced by the National Institute of Standards and Technology (NIST),[58] supporting practical resistance against exhaustive prediction-based attacks (see Methods for details).
